# A One Health Perspective on *Proteus mirabilis*: The Interaction of Virulence and Antimicrobial Resistance Across Human and Animal Reservoirs

**DOI:** 10.3390/microorganisms14020444

**Published:** 2026-02-12

**Authors:** Ibtisam Faeq Hasona, Amal Awad, Gamal Younis, Wafaa Farouk Mohamed

**Affiliations:** 1 Department of Bacteriology, Immunology, and Mycology, Faculty of Veterinary Medicine, Mansoura University, Mansoura 35516, Egypt; gamalyounis_2006@yahoo.com; 2 Ain-Shams University Specialized Hospital, Cairo 11588, Egypt; wafaa3010@yahoo.com

**Keywords:** *Proteus mirabilis*, antimicrobial resistance (AMR), one Health, virulence factors, urinary tract infection (UTI), multidrug resistance (MDR), zoonotic transmission, food safety

## Abstract

*Proteus mirabilis* (*P. mirabilis*), a common commensal and opportunistic pathogen, circulates freely across interconnected human, animal, and environmental reservoirs, embodying the One Health concept. Its key virulence factors—urease activity, swarming motility, and biofilm formation—drive severe urinary tract infections, particularly catheter-associated ones. These virulence traits concurrently facilitate the acquisition and dissemination of antimicrobial resistance (AMR) via mobile genetic elements, leading to extensively drug-resistant clones. Epidemiological and genomic evidence confirms that identical multidrug-resistant clones and resistance mechanisms (ESBLs, carbapenemases) are shared among human clinical isolates, livestock, food products, and environmental samples. This demonstrates continuous, multi-directional transmission through interconnected zoonotic, foodborne, and environmental pathways. The synergistic convergence of potent virulence and escalating AMR within shared reservoirs heightens public health risks. Effective containment therefore demands integrated One Health strategies: enhanced cross-sectorial surveillance, stringent antimicrobial stewardship, robust infection control, and the creation of novel treatments. A coordinated global response is crucial to curb the spread of resistant *P. mirabilis* and preserve antibiotic efficacy.

## 1. Introduction

Globally, the incidence of zoonoses is estimated worldwide to be about a billion cases per year [1], causing enormous pressure, especially in low- and middle-income countries (LMICs). One of the decisive factors here is foodborne disease. According to the World Health Organization (WHO), annually, foodborne illnesses amount to 600 million cases with 420,000 deaths [2,3]. This is due to the production of pathogenic bacteria of more than 50%, amounting to over 300 million cases of human infections every year, directly attributed to livestock production [2]. The ramifications extend beyond public health to substantial and global economic losses. This burden is profound across all income levels but disproportionately affects LMICs. The national economic toll is significant, with total annual costs estimated at $723 million in Ethiopia and $391 million in Burkina Faso [4,5]. Collectively, analyses indicate that the total annual economic cost of foodborne diseases across all LMICs reaches approximately $110 billion, comprising about $15 billion in direct costs and up to $95.2 billion in indirect costs annually [6,7].

**Figure 1:**
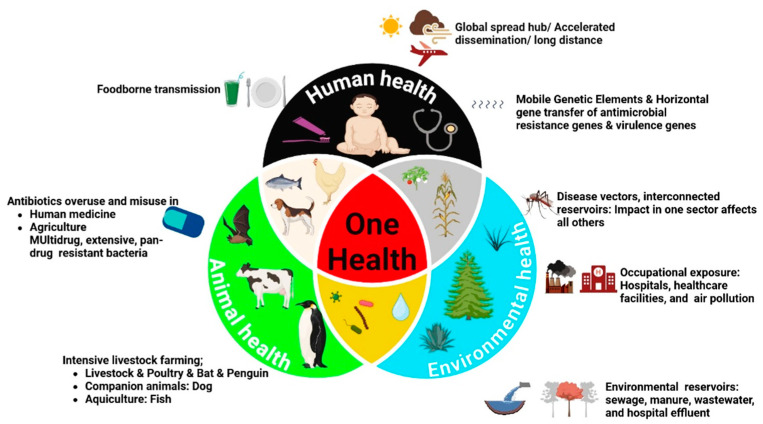
The figure illustrates the interconnected drivers and pathways facilitating the development and spread of multidrug-resistant (MDR) *P. mirabilis* across human, animal, and environmental reservoirs. Key drivers include the overuse and misuse of antibiotics in human medicine and agriculture. The global dissemination of MDR *P. mirabilis* is facilitated by mobile genetic elements, which enable the horizontal transfer of resistance genes across all sectors, underscoring the inextricable link between human, animal, and environmental health. Transmission occurs through foodborne routes, occupational exposure, environmental contamination (e.g., wastewater, manure), and contact with diverse animal hosts (livestock, companion animals, aquaculture). The schematic underscores the necessity of integrated “One Health” interventions to disrupt this continuous cycle. The schematic underscores the necessity of integrated “One Health” interventions to disrupt this continuous cycle. Visual Guide (Legend): For clarity, the schematic uses specific icons and connectors to represent key concepts: A globe or network hub denotes the “Global Spread Hub” enabling long-distance dissemination; A food item (e.g., fish) represents “Foodborne Transmission”; A DNA helix/plasmid symbol illustrates “Mobile Genetic Elements and Horizontal Gene Transfer”; A pill “Antibiotics Overuse and Misuse” in human and agricultural sectors; Icons of livestock (cow), poultry (chicken), bat, penguin, dog, and fish correspond to the diverse “Animal Reservoirs” (livestock, wildlife, companion animals, and aquaculture); Interlocking circles or a unified symbol embodies the “One Health” approach; An insect (e.g., mosquito/fly) depicts “Disease Vectors” and interconnected reservoirs; A hospital building or medical worker icon indicates “Occupational Exposure” in healthcare settings; Factory buildings and smokestacks represent contributing “Air Pollution” from industrial sources; Icons for water pipes, waste runoff, and manure symbolize “Environmental Reservoirs” (sewage, wastewater, manure, and hospital effluent).

This economic challenge is not confined to LMICs; even in high-income countries with advanced regulatory systems, the toll remains substantial, as illustrated by recent U.S. estimates of $17.6 billion annually for 15 key pathogens [8]. A forward-looking perspective must consider that these costs are projected to rise due to population growth, urbanization, and climate change, factors that may alter pathogen distribution and intensify pressures on global food systems. Therefore, robust estimation of this economic impact is foundational to informed policy-making, evidence-based resource allocation, and the development of effective intervention strategies that address both immediate health needs and long-term economic sustainability [9]. Future research should prioritize generating more localized and comprehensive cost estimates to guide targeted and cost-effective interventions worldwide. Of particular mention here among the opportunistic bacteria causing foodborne illnesses is the *Proteus* spp., a common bacteria observed in different body parts of humans and animals, like the gut, skin, and mouth. It is also a common bacteria observed in waste matter, soil, and plants [10]. *Proteus mirabilis* (*P. mirabilis*) exemplifies a prototype One Health challenge, owing to its capacity for cross-sectoral transmission and exchange of antimicrobial resistance across human, animal, and environmental reservoirs. *P. mirabilis*, a well-known Gram-negative (GN), rod-shape, facultative anaerobic bacterium, belongs to the genus *Proteus* within the family *Morganellaceae*. Traditionally classified in the *Enterobacteriaceae* family, the taxonomy of *P. mirabilis* was reassessed with advancements in genomic analysis, leading to its reclassification under *Morganellaceae* by Adeolu et al. in 2016 based on phylogenetic studies [11]. This organism is a facultative anaerobe, and it has cells that have a dimension of about 0.5 to 1.0 µm and a length of 1.0 to 3.0 µm [12]. The organism is a flexible species that has an optimal growth rate at a temperature of 34–37 °C, and therefore, it thrives very well in the human body because it is a perfect environment for the organism to survive [13]. The organism has a very distinct characteristic of producing a series of concentric rings during swarming motions on solid surfaces, an action that is controlled by a series of at least ten genetic factors for adhesion and peritrichous flagella responsible for the organism’s ability to swim and swarm effectively [14,15]. This multicellular system, whose mode of action involves the production of long swarmer cells by the conversion of the organism’s vegetative cells to elongated cells that help the organism to colonize and spread quickly through the human body, is an important virulence factor [12,15]. This system is very closely associated with the invasion of cells and the production of virulence-associated factors [16]. Furthermore, this unique mode of motion, as well as the ability of the organism to elongate and secrete polysaccharides upon coming into contact with a surface, increases the organism [17]. Given its wide distribution, significant pathogenicity, and escalating role in antibiotic-resistant infections across human and veterinary medicine, a deeper understanding of *P. mirabilis* is urgently needed. Through a One Health lens, this review examines virulence in human and animal hosts, analyzes AMR dynamics across interconnected reservoirs, and evaluates collaborative strategies for containment. By consolidating data on its key characteristics, pathogenic factors, and evolving resistance profile, this work provides a comprehensive analysis framed within the imperative of a unified One Health approach. Figure 1 below illustrates the interwoven process of spreading resistance to *P. mirabilis* among human, animal, as well as environmental segments. The important role of joint activities regarding the risk to health from this MDR pathogen, in both areas of health, is highlighted in this work.

## 2. Clinical Epidemiology and Pathogenic Profile of *P. mirabilis* in Human Infections

*P. mirabilis* has the highest isolation rate and geographical range among its kind and has been identified as a normal flora of the intestinal tract of both human beings and animals [12,13]. It has a remarkably versatile range, inhabiting soil, water, and sewage, where the breakdown of organic matter contributes to its metabolic activity [12,18].

The major clinical relevance is due to its existence as one of the leading opportunistic pathogens that cause urinary tract infections (UTIs). It is a leading cause of both community-acquired and healthcare-associated UTIs and is typically recognized as the second most prevalent *Enterobacterales* species isolated after *Escherichia coli* (*E. coli*) [19], account for 1–10% of all human UTI cases [20]. In complex circumstances, infection rates rise dramatically. Following urinary catheterization, the incidence of catheter-associated UTIs (CAUTIs) caused by *P. mirabilis* can increase to between 20% and 45% or more [12,17,21,22]. Although CAUTIs are typically polymicrobial, *P. mirabilis* is one of the most commonly detected pathogens [23] and constitutes the predominant causative agent (10–44%) in double-J stent-associated urinary tract Infections (DJUTIs) [24].

These infections are more prevalent in patients with long-term catheter usage, with the highest incidence in older patients and women [12,18,20]. *P. mirabilis* causes 1–2% of UTI infections in healthy adult women, but accounts for 5% of hospital-acquired UTI cases in hospitalized females [12]. CAUTIs that arise after UTIs have a substantial mortality rate [25]. Cystitis, bacteriuria, acute pyelonephritis, catheter blockage, and fever are some of the symptoms [20]. The underlying reasons for these demographic disparities are multifactorial. The heightened incidence among elderly patients is primarily driven by age-associated factors such as increased comorbidities (e.g., type 2 diabetes, neurological disorders), immunosenescence (the natural decline of immune function with age), and, most critically, a greater prevalence of instrumentation like long-term urinary catheterization, which provides a direct portal for infection and is a major risk factor for *P. mirabilis* CAUTI [17,18,26,27]. Conversely, the higher prevalence in women, especially during reproductive years (ages 20–50), is largely attributed to anatomical differences—namely, a shorter urethra that facilitates bacterial ascension into the bladder—and possibly hormonal influences [28].

*P. mirabilis*’ strong urease enzyme is a major contributor to UTI complications. This enzyme hydrolyzes urea into ammonia and carbon dioxide, increasing urine pH and precipitating struvite or apatite crystals [12]. These crystals consolidate into bladder stones, which operate as long-term bacterial reservoirs, making infections difficult to treat and likely to recur. This procedure can also result in catheter encrustation and blockage, which may lead to hydronephrosis and kidney injury [18,29]. Urolithiasis in the bladder and kidneys can cause irreversible kidney damage and frequently requires surgical intervention [18,30].

When *P. mirabilis* invades the host body, it releases endotoxins that enter the bloodstream, triggering the host’s inflammatory immune response and resulting in conditions such as sepsis or systemic inflammatory response syndrome (SIRS), which have a mortality rate of approximately 20–50% [17]. Infections can lead to more dangerous illnesses including bacteremia and sepsis, either through direct tissue damage or bacterial migration on catheter surfaces [12,17]. *P. mirabilis* is responsible for 5–18% of all Gram-negative bacteremia cases [12,31]. Bacteremia and sepsis induced by this bacterium are associated with a significant death rate, reaching up to 50% in older people, which is relatively greater than mortality from other infectious sources [18,25,30,32,33]. The progression to severe systemic infections such as septicemia typically occurs in vulnerable patient populations with compromised host defenses. Key predisposing conditions include advanced age, diabetes mellitus, malignancy, and other immunocompromised states, as well as exposure to healthcare settings and invasive devices such as urinary catheters [34,35].

*P. mirabilis* causes a variety of infections outside of the urinary tract, especially in immunocompromised people. Approximately 90% of clinical isolates arise from UTIs and CAUTIs, alongside the remaining 10% related to infections of the respiratory tract, eye, ear, nose, skin, burns, wounds, meningoencephalitis, and osteomyelitis [32]. Its swarming mobility promotes tissue colonization and spreads [12]. It has also been linked to diarrhea, infective endocarditis, rheumatoid arthritis, and hospital-acquired epidemics [14,22]. Additionally, *P. mirabilis* has been implicated as a cause of food poisoning incidents, accounting for 3.61% of reported cases in Datong, China between 2016 and 2017, with symptoms such as abdominal pain, diarrhea, nausea, and dizziness [36]. In a 2018 incident in Beijing, contamination of braised meatballs with *P. mirabilis* led to illness among customers, with the bacterium detected on the hands of the chef and waitstaff [37]. Proliferation is significantly easier in immunocompromised people or those getting antibiotic therapy [18,38]. According to one study, *P. mirabilis* was isolated from 21.6% of cutaneous abscesses, making it the second most prevalent isolate after methicillin-sensitive Staphylococcus aureus [39]. Potential linkages to Crohn’s disease (CD) and intestinal inflammation aggravated by oral inflammation or proton pump inhibitor use have also been reported [40]. Subsequent studies have strengthened this link; comparison of feces and inflamed colon samples from CD patients and healthy individuals revealed a significant increase in the abundance of *P. mirabilis* in CD patients. Experimental results showed signs of colon shortening, and liver and spleen enlargement, indicating that *P. mirabilis* plays a critical role in inducing CD inflammation [39]. Furthermore, it has been suggested that oral inflammation could exacerbate intestinal inflammation, and that the use of proton pump inhibitors may promote the proliferation of *P. mirabilis* and other microbes in the intestines, thus triggering intestinal inflammation [41]. Regarding diarrhea, another study isolated *P. mirabilis* from 49 out of 486 pediatric diarrheal samples, yielding a detection rate of 10.1% [42].

## 3. The Pathogenic Spectrum and Host Range of *P. mirabilis* in Animals

*P. mirabilis* has been detected in a variety of hosts, including pets, ruminants, and poultry, demonstrating its remarkable versatility in the field of veterinary medicine. Its host range extends far beyond domesticated animals, encompassing a wide array of wildlife, which underscores its broad ecological niche and zoonotic potential. It has been isolated from wildlife in undisturbed habitats, such as Egyptian vulture chicks in the Canary Islands, migratory bird feces in China, and a fruit bat in Indonesia [43]. In wild mammals, it has been found in wild boars in Tunisia and in gorillas, mandrills, and African buffaloes in a national park in Gabon [44]. Notably, its presence in raptors in Spain and in a juvenile sea lion in Uruguay indicates its reach across diverse species and ecosystems [45]. Furthermore, demonstrating its threat to even rare and protected species, a multidrug-resistant strain of *P. mirabilis* (PM2022) was identified as the cause of fatal lobar pneumonia and hepatic necrosis in a critically endangered Malayan pangolin. Genomic analysis of this strain revealed a concerning array of antibiotic resistance genes, including those encoding for extended-spectrum beta-lactamase (CTX-M-65), and confirmed the presence of key virulence factors [46]. The detection of *P. mirabilis* in ticks collected from wild mammals and cattle suggests ectoparasites may play a role in its transmission [47]. In controlled environments such as zoos and farms, it has been identified in species including giant pandas and red pandas in China, and farmed foxes, raccoons, and minks [48]. Notably, in zoo settings, *Proteus* spp. can cause severe systemic infections; a fatal case of septicemia in a Humboldt penguin was attributed to a concomitant infection by *P. mirabilis*, *P. penneri*, *P. vulgaris*, and *P. cibarius*, highlighting the potential for severe, multi-species Proteus infections in captive wildlife [49]. It is also present in research animal models like diarrheal rhesus macaques, ferrets [50], and tree shrews [51].

Farm animals constitute a significant reservoir for *P. mirabilis*, with implications for animal health and food safety. Studies have reported its isolation from pigs in various regions [52,53], and it has been found in boar semen, where it negatively impacts sperm quality [54]. Research indicates a notable presence of *P. mirabilis* in samples from diseased pigs, highlighting its potential pathogenic role [55]. The bacterium is also commonly isolated from healthy and diseased poultry, cattle, and sheep across different countries, with reports from chicken flocks, duck populations, and livestock manure underscoring its widespread occurrence in agricultural settings [52,56,57]. Its association with clinical disease, including diarrhea in poultry and cattle, further points to its importance as an agent affecting livestock health and productivity [55,56].

Companion animals, due to their close contact with humans, represent another critical reservoir and a potential source of zoonotic transmission. *P. mirabilis* is frequently detected in pets. It has been co-isolated from humans and dogs in shared households [58] and is commonly found in fecal samples from both household and stray dogs [59]. Research indicates that the frequency of intestinal colonization with *P. mirabilis* is significantly higher in dogs compared to their human cohabitants. Importantly, molecular studies have confirmed the sharing of genetically related *P. mirabilis* strains between humans and dogs living in the same household, with some of these shared fecal strains also showing genetic relatedness to clinical uropathogenic strains. This provides direct evidence of cross-species transmission and underscores the dog’s role as a potential reservoir for human infections [58]. A focused study on UTIs found a distinct prevalence pattern: while *P. mirabilis* was not isolated from dogs with UTIs in that cohort, it was identified in 4% of cats with UTIs, and at a much higher rate (26%) in human UTI cases, highlighting species-specific differences in urinary tract colonization [60]. Exotic pets, such as turtles, have also been shown to carry the bacterium [61]. A primary health concern is UTI. *P. mirabilis* is a notable cause of UTIs in dogs and, to a lesser extent, in cats, as reported in multiple studies across different continents [62,63,64]. In veterinary medicine, this association is particularly significant as *P. mirabilis* has been specifically linked to the formation of recurrent urinary stones in dogs with urinary system disorders [65]. Beyond UTIs, the bacterium is also associated with gastrointestinal issues like diarrhea in dogs [66] and has been identified in cases of co-infection with major viral pathogens [67].

In the aquaculture sector, *P. mirabilis* is emerging as a significant primary pathogen across key farmed fish species, characterized by severe disease. It causes high mortality (up to 68.8%) and systemic infections in African catfish (*Clarias gariepinus*) with clinical signs like skin hemorrhages and ulceration, leading to hepatic/renal damage and anemia [68,69]. Its prevalence in Nigerian catfish farms is significant (13–31%) [68]. In Nile tilapia (Oreochromis niloticus), infection induces renal–hepatic dysfunction, oxidative stress, and histopathological damage [70], though a separate tilapia strain showed probiotic potential against *Vibrio* spp. [71]. In Indian major carp (Labeo catla), it is a confirmed lethal pathogen [72]. This, coupled with its detection in freshwater fish sold for consumption [73], underscores a direct food safety threat. The ecological role appears complex, as co-infection with *Aeromonas hydrophila* (*A. hydrophila*) in catfish showed antagonistic interaction, resulting in lower mortality than a single *A. hydrophila* infection [74].

Animal-derived foods have become a major focus of public health concern due to their contamination with *P. mirabilis*. The prevalence of the bacterium in meat and other products varies significantly across different countries and regions, reflecting differences in hygiene practices and environmental conditions. Significant contamination has been reported in various meat types: in India [52], Iran [75], Brazil [76], Egypt [77,78,79], Belgium [80], and China [81,82,83,84]. Chicken meat often shows high contamination rates in these studies, followed by pork and beef. These findings indicate that poor hygiene at poultry and meat stalls may result in substantial contamination and cross-contamination, particularly affecting poultry products. A recent study in China also reported contamination of *P. mirabilis* in retail meat and aquatic products [82]. Insects such as flies associated with animal-derived food also appear to serve as potential vectors of transmission [85]. Furthermore, the presence of *P. mirabilis* in wildlife such as wild boars and African buffalo [86], and in freshwater fish sold in markets [73], which are sources of game meat and aquatic products, suggests a potential route of zoonotic transmission from wildlife to humans, although this possibility requires further investigation.

The pathogenicity of *P. mirabilis* in animals is well-documented across multiple organ systems. As a gastrointestinal pathogen, it has been responsible for severe outbreaks. Notably, it caused the deaths of 400 bamboo rats on a farm in Guangdong, China, presenting with vomiting and diarrhea [87]. Similarly, infections in rabbit farms in China have led to mass fatalities, with affected animals showing lethargy, yellow watery diarrhea, and multi-organ tissue damage by strain HN001 [88] and strain T2018 [89]. In non-human primates, *P. mirabilis* infection in rhesus monkeys resulted in diarrhea and bloody stools [50]. Furthermore, a specific strain (17f) was identified as the primary cause of diarrhea in lambs in Xinjiang, China [57]. Beyond the gastrointestinal tract, *P. mirabilis* can cause respiratory disease in animals; in pigs, specific strains have been associated with respiratory symptoms such as fever and difficulty breathing [90,91]. Alarmingly, studies in pigs also indicate that *P. mirabilis* can cross the placental barrier, leading to fetal death [90,91].

Aside from gastrointestinal difficulties, *Proteus* spp. Cause a variety of other disorders in chickens. These include embryonic death, yolk sac infections, and mortality among young chickens, turkeys, and ducks [92]. They also cause granulomatous inflammation in the salt glands of waterfowl, quails, and broilers, as well as disorders like arthritis, salpingitis, airsaculitis, and septicemia, which results in carcass condemnation and economic losses for the poultry sector [92]. The bacteria are also responsible for serious, less common illnesses in other animals; for instance, Abdollahi et al. and Najd et al. were the first to report cases of *P. mirabilis*-induced pyoderma and purulent pericarditis in sheep [93,94]. More generally, *P. mirabilis* is involved in different diseases in animals, which include UTIs, wound infections, and gastrointestinal disorders, with special significance in poultry and livestock [95].

*P. mirabilis* has pathogenicity in many types of tissues and organs, and very serious infections may lead to death. The most important role of *P. mirabilis* is that it is one of the common pathogens that cause UTIs in humans and in domesticated pets (e.g., cats and dogs) [96]. Herout et al. reported a high prevalence of *P. mirabilis* in a mouse model of CAUTI, demonstrating that *P. mirabilis* is perpetually involved in UTIs in broad range of species [97]. The risk of becoming infected with *P. mirabilis* is equally high for both animal and human populations. The continued rise of antibiotic-resistant strains of *P. mirabilis* in both animals and humans reinforces the importance of understanding how *P. mirabilis* causes disease and the mechanisms by which it acquires antibiotic resistance. The information presented in this review supports the conclusion that *P. mirabilis* is a significant contributor to the development of gastrointestinal disease and UTI in animals and therefore poses a serious threat to food safety and animal welfare and public health. In addition to infecting the gastrointestinal and urinary systems, many other diseases caused by *P. mirabilis* involve many other organ systems. Furthermore, the broad geographic and host range of *P. mirabilis*—now conclusively extended to include aquatic ecosystems where it acts as a primary pathogen with concerning multidrug-resistance profiles—along with its pervasive contamination of the food chain, highlights its dynamic ecology and formidable potential for cross-species transmission. This evidence base solidifies the imperative for a comprehensive “One Health” approach to combating the health problems associated with *P. mirabilis*. Finally, the array of environmental conditions that can sustain *P. mirabilis* indicates that this bacterium has a wide range of opportunities to infect new hosts and cause disease.

## 4. Geographic and Host Variations in Virulence-Associated Genes and Factors of *P. mirabilis*

The virulence arsenal of *P. mirabilis* is not only central to its pathogenicity in human infections but also plays a critical role in animal diseases, creating a common biological foundation for cross-species transmission and persistence within the One Health framework. The pathogenic potential of *P. mirabilis* is underpinned by a diverse repertoire of virulence-associated genes (VGs), whose distribution varies by both geography and host species. Epidemiological studies indicate notable variation in VGs profiles. For instance, isolates from North America (United States and Canada) and from poultry sources have been reported to harbor higher numbers of VGs compared to other regions and hosts, highlighting the role of specific reservoirs in shaping virulence [95].

**Figure 2:**
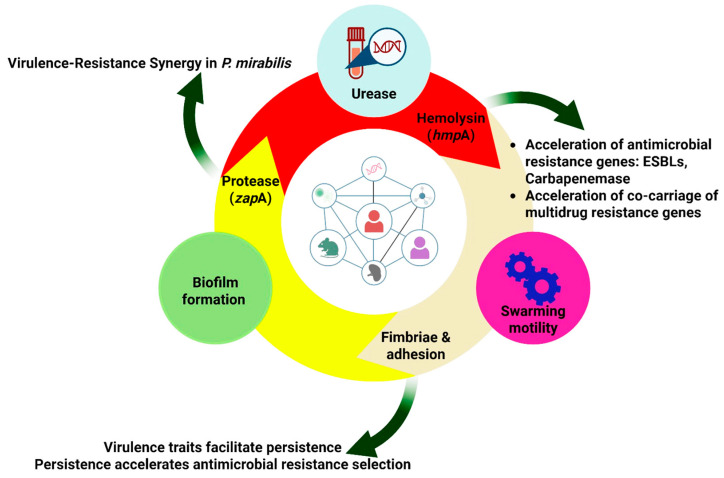
The synergistic cycle between virulence factors and antimicrobial resistance (AMR) selection in *P. mirabilis*. The diagram illustrates how key virulence factors (urease, hemolysin *hmp*A, protease *zap*A, biofilm formation, adhesion factors, and swarming motility) enhance bacterial persistence within the host and environment. This prolonged persistence increases the exposure time to antimicrobial agents, thereby accelerating the selection and co-carriage of MDR genes, including those encoding extended-spectrum beta-lactamases (ESBLs) and carbapenemases. The model depicts a self-reinforcing cycle wherein virulence traits promote survival under selective pressure, and the subsequent acquisition of resistance further stabilizes virulent, persistent lineages of *P. mirabilis*. In this schematic, specific icons are used for clarity: the glass tube represents urease activity, a key virulence factor involved in urinary alkalinization and struvite stone formation; gears symbolize swarming motility, which facilitates rapid surface translocation and biofilm dispersal; the DNA helix denotes the genetic co‑localization of virulence and antimicrobial resistance genes on mobile genetic elements, enabling their co‑transfer and co‑selection; the mouse and human icons represent animal and human hosts, respectively, highlighting zoonotic transmission and cross‑species interfaces; the overlapping arrangement of human, animal, and DNA icons within the central cycle visually expresses the “One Health” convergence at the molecular level, illustrating that virulence and resistance genes circulate together across human, animal, and environmental reservoirs; the large enclosing circle and its internal interwoven arrows and nodes depict a self‑perpetuating, multidimensional network wherein virulence enhances host persistence, persistence increases antimicrobial exposure, exposure selects for resistance, and resistance stabilizes virulent lineages, with all traits co‑transmitted from host to host and reservoir to reservoir; solid arrows indicate direct causal or stimulatory pathways (e.g., virulence factor → enhanced persistence → increased antimicrobial exposure → resistance selection), while curved or circular arrows represent self‑reinforcing feedback loops, demonstrating how the expression of virulence traits and the acquisition of resistance continuously reinforce one another in a closed, cyclical manner. This integrated visual framework emphasizes that the synergy between virulence and resistance is not linear but operates within a dynamic ecological and molecular continuum.

A complex collection of factors is critical for the pathogenicity, persistence and colonization of *P. mirabilis* (Figure 2), particularly for the urinary tract. Some of these virulence factors include the ability to adhere to tissue, form a biofilm, produce hemolysin and urease, exhibit flagellar motility, swarm and utilize fimbriae to attach themselves [13,18,27,33].

Initial attachment occurs through attachment pili, flagella, and fimbriae, while outer membrane proteins, such as adhesins and lectins, attach to surfaces of the host. There are specific mechanisms necessary to attach to uroepithelial cells that result in the production of UTIs where bacteria are able to grow within the bladder, overcome the effects of urine flow, and establish chronic infection [98]. In addition to adhesive factors, some of the virulent strains develop protective capsules that protect themselves from host defense mechanisms, such as phagocytes [99], while others utilize antigenic variation to evade immune response [100]. This coordinated interplay of virulence factors enables *P. mirabilis* to overcome host defenses, establish infection, and often lead to chronic, difficult-to-treat conditions. A concise overview of the core virulence machinery of *P. mirabilis* is presented in Table 1.

As summarized in Table 1, the pathogenicity and clinical severity of a *P. mirabilis* infection are not strictly dependent on the simultaneous presence of all virulence factors but are rather determined by a dynamic and often synergistic interplay among them. This sophisticated molecular interplay efficiently enhances its virulence and underscores its significance in disease development [18]. While basic colonization may be achieved through key adhesins (e.g., MR/P fimbriae) and motility, the progression to severe, complicated disease—such as catheter-associated pyelonephritis, struvite urolithiasis, or systemic sepsis—typically requires a coordinated expression of multiple factors [21]. For instance, urease activity is indispensable for stone formation and catheter encrustation, hemolysin and protease are critical for tissue invasion and immune evasion during systemic spread, and robust biofilm formation underpins chronicity and treatment failure. The swarming phenotype acts as a master regulator, often upregulating the expression of other virulence determinants (urease, protease, hemolysin), thereby linking motility directly to enhanced pathogenic potential.

From a practical and diagnostic perspective, the pathogenic risk posed by a clinical *P. mirabilis* isolate can be inferred by profiling its virulence gene repertoire. Molecular screening for key genes—such as those encoding urease (*ure*C), hemolysin (*hmp*A), MR/P fimbriae (*mrp*A), protease (*zap*A), and biofilm-associated functions—allows for stratification of isolates into risk categories [18]. Isolates harboring a full complement of these genes are classified as high-risk, particularly in settings involving indwelling devices or immunocompromised hosts, as they possess the genetic arsenal for persistent colonization, tissue damage, and antibiotic tolerance. Conversely, isolates lacking critical genes (e.g., *ure*C) may be considered lower-risk for causing complicated infections, though they retain the capacity for acute cystitis. This genotype-to-phenotype correlation underscores the potential of virulence factor profiling as a tool for prognostic assessment, guiding infection control measures, and personalizing therapeutic strategies in both human and veterinary medicine within the One Health framework. The subsequent Section 4.1, Section 4.2, Section 4.3, Section 4.4, Section 4.5 and Section 4.6 provide a detailed examination of each major factor, elucidating its specific role in pathogenesis and its contribution to the bacterium’s success across different hosts within the One Health paradigm.

### 4.1. Urease as a Central Virulence Factor in P. mirabilis Pathogenesis

*P. mirabilis* exploits a range of virulence agents, with the cytoplasmic nickel metalloenzyme urease being particularly important for pathogenesis. This enzyme, situated in the cytoplasm or outer membrane, catalyzes the hydrolysis of urea into ammonia and CO_2_, resulting in a large local pH increase [18,101]. The ammonia produced is extremely alkaline and immediately cytotoxic to mammalian cells, producing significant tissue damage and compromising the uroepithelium, facilitating bacterial invasion [102].

The relationship between urease activity and pathogenicity is well explained in the development of urinary stones [103]. These stones create a niche in which bacteria are protected from human immune response and drugs, thereby leading to persistent and recurrent infections [12].

Urease activity in medical devices precipitates minerals, which interact with bacteria on urinary catheters and produce a crystalline biofilm that obstructs urine flow [104], also to the development of acute pyelonephritis [12,27]. The crystalline biofilm is responsible for the encrustation and blockage of catheters, a common result from CAUTIs. The bacteria embedded in such crystalline formations are resistant to drugs as well as to the human immune response. In a polymicrobial environment, interactions may become synergistic, enhancing overall pathogenicity even when bacterial loads are equivalent to that of mono-species infections [104]. The understanding of these detailed virulence pathways offers promising options for the design of future therapies against UTIs [105]. This stone-forming capability, critical in human CAUTIs, poses similar risks in animals with urinary catheters or anatomical abnormalities, underscoring a shared pathological mechanism across species. The *P. mirabilis* urease gene cluster consists of structural genes such as *ure*ABC and the urea-induced regulator *ure*R, which can be detected by PCR techniques [106].

### 4.2. Adhesive Structures and Biofilm Formation in P. mirabilis: Key Fimbriae and Their Roles

Fimbriae are hair-like structures on the bacterial cell surface; they are essential for the adhesion of *P. mirabilis* bacteria to the bladder and renal epithelium and to other inert substances such as catheters [18,21]. Chaperone-usher fimbriae are particularly important for the molecularly precise adhesion and colonization of the urinary tract epithelium by bacteria [18]. These organelles are critical for biofilm production, assisting in immune evasion and persistent infection, and contribute to crystalline biofilm and catheter encrustation characteristic of *P. mirabilis* UTIs and infectious stone formation, highlighting their pathogenic relevance [18,107].

Important fimbrial adhesins are necessary for initial colonization and biofilm formation. These include *P. mirabilis* fimbriae (PMF, *pmf*A), Ambient-Temperature Fimbriae (ATF, *atf*A), Uroepithelial Cell Adhesin (UCA, *uca*A), Mannose-Resistant/*Proteus*-like (MR/P) fimbriae (encoded by *mrp*A), and Non-Agglutinating Fimbriae (NAF) [108]. The genes *mrp*A, *pmf*A, *uca*A, and *atf*A are key molecular targets detectable using PCR [109]. MR/P fimbriae are particularly important for bladder and kidney biofilm production and colonization and play a critical role in catheter biofilm establishment [21].

PMF fimbriae, first discovered in strain HI4320 [110], are made easier to assemble and locate in the urinary tract by a five-gene operon (*pmf*A, *pmf*C, *pmf*D, *pmf*E, and *pmf*F) [108,111]. Other fimbriae allow for niche-specific colonization: UCA improves adherence to uroepithelial cells, NAF boosts in vitro adhesion, and ATF promotes environmental survival through optimum expression at lower temperatures [108]. P-like pili (PMP) are another adherent agent that may help with urinary tract adhesion [112], and MR/K hemagglutinin, enabling catheter attachment [102].

Aside from chaperone-usher fimbriae, *P. mirabilis* has a possible type IV pilus system—a dynamic structure implicated in virulence, twitching motility, and biofilm generation, with functional similarities to MR/P fimbriae [18,113]. Genomic investigations reveal one or two probable type IV pilus loci [114]. This system acts by pilin polymerization/depolymerization and includes components including major/minor pilins, a pre-pilin peptidase, assembly/retraction ATPases, a secretin and auxiliary proteins [115]. The adhesive and biofilm-forming capabilities of *P. mirabilis* are equally critical in veterinary settings, facilitating persistent urinary tract and wound infections in companion and livestock animals, thereby contributing to the spread of resistant strains across species.

### 4.3. Swarming Motility: An Overview

*Proteus* spp. have a dimorphic life cycle, which is essential for their pathogenicity. They appear in liquid settings as short, motile swimmer cells with peritrichous flagella. When cells come into contact with a solid surface, they undergo complicated differentiation and become elongated (20–80 µm), hyperflagellated, multinucleated swarmer cells. This differentiation entails continuous DNA replication in the absence of cell division, resulting in polyploid filaments. These cells move together in coordinated rafts and form characteristic concentric rings on agar. The migration cycle of swarming cells is divided between periods of migration and consolidation, wherein cells temporarily readopt a shorter cell form prior to further differentiation or not. This process, important for the movement into new areas and host colonization [108,110,116]. Elaborating on this key characteristic, the regulatory dynamics of swarming involve the differentiation of *P. mirabilis* controlled by a network activated by specific signals. The primary trigger is surface contact, sensed via restricted flagellar rotation, which upregulates regulators like *umo*B and *lrp* [117]. Without this signal, cells remain as short “swimmers” [33]. These regulators drive genes for elongation, hyperflagellation, and polysaccharide production [117]. Chemical inducers include L-glutamine, other amino acids, and putrescine [33]. Zinc homeostasis is also crucial [118]. Conversely, high osmolarity represses swarming, and high urea may inhibit swarming in vivo [36]. Swarming upregulates virulence factors: urease, *zap*A protease, and hemolysin [117], promoting stone formation [119], host cell lysis [117], and protein degradation. Additionally, swarming initiates biofilm formation on catheters [120], leading to persistent infections.

Swarming has a closely regulated mechanism associated with higher pathogenicity. In the transition from swimming to swarming mode, there is a higher expression of flagella and a regulated expression controlled by a tier of genes, with the primary role associated with the master regulatory genes *flh*DC. Another key gene associated with the unique feature of cell elongation is *ccm*A. Beyond the swarming motility is the cell’s metabolic and pathogenic shift for differentiated swarmer cells. Compared with swimmers, there is a higher production of key virulence factors urease, protease, hemolysin (*hmp*A), and IgA-degrading metalloprotease (*zap*A) associated with swarmer cells. The association between motility and the production of virulence factors underlines the vital role of swarming in the infection process [33,117].

The swarming of *P. mirabilis* is extremely responsive to environmental stimuli, especially those presented by its host. Certain chemicals in human urine have been identified as effective stimulators of swarm motility, including arginine, glutamine, histidine, malate, and ornithine. Another significant stimulator is the polyamine putrescine, whose biochemical pathway is unusually connected with urea metabolism-one of the key processes underlying pathogenesis in the urinary tract. This indicates that the urinary system’s chemical environment imposes a direct influence on the invasive behavior of the bacterium. Additionally, physical mobility across surfaces by swarm rafts is aided by chemicals such as capsular polysaccharides and slime [12,108,113].

The swarming phenotype has significant clinical implications, especially in CAUTIs. *P. mirabilis* can quickly spread across the surfaces of silicone or latex urinary catheters, allowing for fast colonization and ascent into the bladder and kidneys. This surface movement is thought to be associated with increased expression of virulence genes, potentially beginning infection [121]. Swarmer cells are significantly more invasive to urothelial cells than vegetative swimmer cells. Flagella-driven motility has an important role in virulence, as evidenced by research demonstrating that flagella-negative mutants are much less harmful. As a result, the ability to swarm is an important contributor to the creation of strong biofilms, the spread of infection, and improved antibiotic resistance in chronic infections [12,18,120]. This swarming capability, while extensively studied in human CAUTIs, likely facilitates similar rapid colonization and biofilm formation in animal hosts with urinary catheters or anatomical susceptibilities, highlighting a conserved invasion strategy.

### 4.4. Hemolysin as a Critical Virulence Factor in P. mirabilis: Roles in Cytolysis, Iron Acquisition, and Pathogenesis

Hemolysin is an important multifunctional virulence factor in *P. mirabilis*, principally through the action of a calcium-independent, Serratia-like toxin produced by the *hmp*A gene. The production of this hemolysin is promoted by its specialized transporter, encoded by the highly conserved *hmp*B gene, indicating the importance of this system in pathogenesis [108,117]. These genetic markers (*hmp*A and *hmp*B) are successfully discovered using polymerase chain reaction [109]. Hemolysins, which have a wide range of cytotoxic effects, possess the capability to lyse not only nucleated host cells but also erythrocytes [122]. This membrane-disrupting capability serves two functions: it liberates important nutrients such as iron from red blood cells and thus supports bacterial survival; and it promotes the invasion of tissues and the evasion of immunity through the destruction of host defense cells and the creation of pathways for disseminating bacteria within the host [12,117]. The significant reduction in virulence observed with *hmp*A knockout mutants illustrates that the hemolysin contributes significantly to the process of infecting a host [18,123]. Thus, it can be concluded that hemolysins are a major factor in determining both the severity and persistence of infections caused by *P. mirabilis*, making the mechanisms by which they function an appropriate target for the development of potential therapeutic interventions [31,108]. The Iron-acquisition function of hemolysin is particularly vital for bacterial survival in the iron-limited environments of both human and animal hosts, highlighting a conserved virulence strategy.

### 4.5. Protease-Mediated Immune Evasion: A Key Virulence Strategy of P. mirabilis

*P. mirabilis* employs proteases as virulence factors to overcome the highly unfavorable urinary tract environment successfully [124]. Of great significance among these proteases is *zap*A (mirabilysin), a potent metalloprotease that directly damages the host’s immunity by selectively degrading vital host defense proteins like immunoglobulins—IgA and IgG—as well as complement factors [124]. When this *zap*A is overexpressed during swarming differentiation processes, its production is consistently high at the edge of a motility bacterial swarm [125]. These targeted actions of proteases and especially *zap*A’s degradation of vital host factors have benefited bacterial escape and persistence and are consequently important in the host’s defense against this pathogen and have been identified as a biotarget/drug target for potential treatment [12], whose development as a drug target is further aided by the discovery of targeted chemicals against this enzyme [124]. This immunoglobulin-degrading strategy, crucial for evading human immune defenses, likely supports bacterial persistence in animal hosts as well, underscoring a conserved immune evasion mechanism within the One Health context.

### 4.6. Biofilm Formation and Quorum-Sensing Regulation in P. mirabilis: A Foundation for Persistent Infection

The process begins when free-floating (planktonic) bacteria encounter a surface. This initial attachment is reversible and weak, driven by physical forces like van der Waals interactions, hydrophobic effects, or electrostatic charges. Bacteria use appendages such as flagella or pili to approach the surface [11]. Once in contact, specific adhesins (proteins on the bacterial surface) may bind to the substrate, making the attachment more secure and irreversible. Environmental factors, like nutrient availability or surface conditioning (e.g., a layer of organic molecules), can influence this step. After attaching, bacteria begin to proliferate and form microcolonies. They divide and produce extracellular polymeric substances (EPSs), a sticky matrix of polysaccharides, proteins, and DNA that anchors them to the surface and to each other. This EPS layer not only provides structural support but also traps nutrients and protects the growing community from external threats like antibiotics or immune responses.

As the biofilm grows, it develops into a complex, three-dimensional structure. This stage involves the formation of water channels within the EPS matrix, which facilitate nutrient and oxygen distribution while removing waste. Genetic regulation fine-tunes the community, with some cells differentiating into distinct roles (e.g., persister cells that resist stress). At this point, the biofilm becomes highly resistant to antibiotics, up to 1000 times more so than planktonic cells, due to the protective EPS and slower metabolic rates of deeper layers [12]. EPS plays a central role in this biological process, and it contributes significantly to biofilm formation as it plays a role in cell and surface attachment and protects against antimicrobials, host immunological defense, and oxidative stress [126].

EPSs mask pathogen-associated molecular patterns (PAMPs), reduce phagocytosis, and neutralize antimicrobial peptides [127]. However, biofilms are most feared in indwelling medical devices such as urinary catheters and implants, which act as a protective barrier referred to as CAUTIs [12,18,127]. Act as reservoirs for chronic infections, necessitating device removal due to resistance to Treatment [128]. Biofilm-mediated UTIs demonstrate high recurrence rates and elevate risks of ureteral injury, drainage dysfunction, and renal failure [129]. Polymicrobial Interactions and functional redundancy further stabilize biofilm signaling under environmental stressors, though disruptions in microbial diversity can impair QS-mediated Cross-kingdom communication [36,113].

Biofilm Antibiotic Tolerance (BAT). As a thumb rule, bacteria in biofilms are really more resistant to antimicrobial intervention than their counterparts in planktonic habitats [22]. Therefore, BAT is expected to incorporate alternate paths to bacterial antimicrobial resistance [25]. Additionally, reduced metabolic activity in deeper biofilm layers diminishes antibiotic Efficacy, while efflux pumps and persister cells enhance survival [122,123,124]. Persister cells further reinforce biofilm resilience, which enters dormancy via a stringent response (ppGpp) and toxin–antitoxin systems (e.g., HipBA). These cells evade antibiotics and repopulate biofilms post-treatment [110,111]. Agr-Mediated dispersal via phenol-soluble modulins (PSMs) contrasts with SarA’s promotion of polysaccharide intercellular adhesin (PIA) [112].

Biofilm regulation is a multifaceted process governed by genetic, biochemical, and Environmental mechanisms, enabling microbial communities to adapt to diverse conditions [92]. Central to this regulation are QS systems and second-messenger molecules, which coordinate the development of biofilms across bacterial species [93]. Cyclic di-GMP (c-di-GMP) is a pivotal second messenger that promotes biofilm stability by upregulating adhesins, pili, and EPS production while suppressing motility [98].

In *P. mirabilis*, the QS system is not fully characterized compared to other GNB. While classic acyl-homoserine lactone (AHL)-based LuxI/LuxR systems are absent [13], the bacterium possesses the *lux*S gene responsible for producing autoinducer-2 (AI-2) [130]. However, disruption of *lux*S does not significantly affect swarming motility or virulence in murine models, suggesting functional redundancy or the presence of alternative signaling pathways [33,130]. Notably, *P. mirabilis* can respond to exogenous AHLs (e.g., N-butanoyl homoserine lactone), which influence biofilm architecture, indicating cross-species communication capabilities [128]. In *P. mirabilis*, QS governs essential characteristics such as the production of virulence factors (e.g., urease, protease, hemolysins) and the growth of the biofilm itself [12,131]. Beyond these roles, QS is implicated in broader physiological processes such as metabolic adaptation and stress responses. Critically for clinical outcomes, QS also contributes to the challenge of antimicrobial resistance through QS-mediated biofilm formation, which promotes antibiotic tolerance by limiting drug penetration and fostering resistant subpopulations. Furthermore, the dispersal phase triggered by QS can facilitate the spread of resistance genes via horizontal gene transfer, as observed in other biofilm-forming pathogens [132,133]. Reverse correlation of biofilm formation ability and swarming motility was estimated. Based on the study findings It is hypothesized that *P. mirabilis* benefited from adhesins such as MR/P fimbriae for production of biofilm and successful colonization and then they shift from biofilm formers to strong swarmers in order to reach deeper urinary organs and *hly*A toxin is used to overcome the immune system cells [134].

In response to these challenges, the scientific community has shifted its focus toward exploring alternative antimicrobial agents that can target biofilms through multifaceted mechanisms [135,136]. Plant-derived essential oils (Eos) have emerged as a promising frontier. These complex phytochemical mixtures exhibit broad-spectrum antimicrobial activity, disrupting biofilms through multiple pathways: destabilizing microbial membranes; inhibiting QS—a critical communication system that regulates biofilm development; and degrading Extracellular matrix components [137,138]. Furthermore, Eos often exhibit synergistic effects When combined with conventional antimicrobials, thereby enhancing drug efficacy while minimizing the development of resistance [139]. Curcumin, which is also derived from Curcuma longa (turmeric), is an anti-quorum Sensing agent that inhibits *P. mirabilis* [140]. Other phytochemicals, such as linalool, derived from floral plants, also inhibit Motility and reduce biofilm-associated extracellular polysaccharides. These compounds could potentially enhance the effectiveness of conventional antibiotics and offer a promising alternative or complementary strategy for controlling *P. mirabilis* infections and biofilm-related Complications [141].

## 5. Antimicrobial Resistance in *P. mirabilis*: Mechanisms, Drivers, Epidemiology, and Transmission from a One Health Perspective

### 5.1. Introduction: The Global and One Health Burden of AMR

The global spread of antimicrobial resistance (AMR) in *P. mirabilis* is a quintessential One Health issue, driven by interconnected human, animal, and environmental reservoirs. AMR poses a serious global challenge, with approximately 60% of all human pathogens and 75% of emerging human infectious diseases having zoonotic origins, and it is noted that most life-threatening human diseases originate from animals [142,143]. The global burden of AMR is staggering, causing an estimated 4.95 million deaths in 2019, of which 1.27 million were directly attributable to resistant infections [144]. Projections suggest AMR could lead to 10 million deaths annually by 2050 if unchecked, with associated economic losses of up to $100 trillion annually [145]. The implications involve the loss of $60–$100 trillion in global output and threaten the achievement of the Sustainable Development Goals [146]. The World Health Organization ranks AMR among the top ten global public health threats [3,147]. In the United States alone, about 2.8 million antibiotic-resistant infections occur each year, resulting in 35,000 deaths and $20 billion in direct healthcare costs [148]. Emerging hotspots for AMR include China, India, Brazil, and Kenya [149,150]. Furthermore, high rates of resistant bacteria in humans are compounded by challenges related to antibiotic use in both animal and human healthcare [150].

The development of AMR represents a significant worldwide public health concern that transcends healthcare and agricultural boundaries [151]. The One Health approach—an integrated strategy considering the interconnected health of humans, animals, and ecosystems—is crucial for tackling infectious diseases like those caused by multi-resistant *P. mirabilis*, which proliferates across hospitals, farms, and the environment [12].

The factors contributing to AMR are diverse and rising exponentially. A primary driver of this crisis is the inappropriate or excessive use of antimicrobials in human healthcare, including over-prescription and self-medication. Inappropriate antibiotic use in primary care settings is highly prevalent, ranging from 15.4% in Canada to 88% in Pakistan [149]. A multi-hospital cohort study found that nearly two-thirds of COVID-19 patients received empirical antibiotics, despite only 3.5% having a confirmed bacterial co-infection [152].

Concurrently, the intensive use of antimicrobials in animal husbandry for therapeutic, prophylactic, and growth-promotion purposes is a critical driver [146]. Globally, animals consume more antibiotics than humans, with livestock accounting for over 73% of total antimicrobial use [149,153]. Notably, major antibiotic classes like tetracyclines, sulfonamides, and fluoroquinolones are excreted in significant proportions unchanged in manure and urine [154,155]. Due to their environmental persistence and low bioavailability in animal guts [156], they maintain biologically active concentrations in soil and water. These pharmacological properties allow them to exert sustained selective pressure for extended periods, thereby promoting the development and maintenance of environmental reservoirs of resistance genes [157]. A major concern is that a considerable portion of administered antibiotics is excreted and can persist in the environment, continuously exerting this selection pressure [155]. While some antimicrobial classes, such as carbapenems, are used exclusively in humans, and others like flavophospholipol and ionophores are exclusively for animals [158,159], most drug classes used in humans, including critical ones like quinolones and broad-spectrum beta-lactams, are also administered to animals [153].

Global antimicrobial consumption is soaring, largely in the human health sector and animal farming. Between 2000 and 2015, global antibiotic usage climbed by 65%, with a 39% rise in consumption rates [160]. The most significant increases were observed in low-income nations, which showed a 56% growth in use, with massive jumps in the consumption of cephalosporins (399%), quinolones (125%), and macrolides (119%) [161]. Estimates also reveal that antimicrobial use in BRICS countries will go up by an astounding 99%, driven by rapid population growth [158]. Current total use stands at about 131,000 tons annually and is set to rise by over 67% to nearly 200,000 tons by 2030 [162]. In 2013, an estimated 131,109 tons of antimicrobials were used in food animals, with the figure expected to climb to 200,235 tons by 2030 [163]. By 2030, antimicrobial use in livestock is anticipated to increase by more than 67% from 2010 levels (around 63,000 tons), exceeding 105,000 tons [158]. The average global annual consumption per kilogram of animal produced is estimated to reach 45 mg/kg for cattle, 148 mg/kg for chickens, and 172 mg/kg for pigs [158]. It is noteworthy that antimicrobial use intensity (mg per kg of animal produced) in cattle was estimated to have decreased by 34% in 2021 [164], although rising overall consumption continues to drive resistance rates.

This widespread antimicrobial selection pressure has a direct and measurable consequence: the proliferation of multidrug-resistant bacteria across the food chain. In aquaculture, a critical unifying concern is multidrug resistance. Isolates from African catfish (*Clarias gariepinus*) have shown high phenotypic resistance indices, with a MAR index of 0.60 [69]. Genomic analysis has confirmed this threat at the molecular level, with whole-genome sequencing of a pathogenic strain from Indian major carp (Labeo catla) revealing it to harbor multiple antimicrobial resistance genes [72]. The contamination of the food supply is equally alarming. A genomic characterization of *P. mirabilis* strains from retail meat and aquatic products in China revealed high genetic diversity. Alarmingly, 91% of the strains exhibited multidrug resistance profiles, carrying a wide array of clinically important resistance genes, including *bla*_CTX-M_, *cfr*, and even genes conferring resistance to tigecycline (*tmexCD3-toprJ1*) and carbapenems (*bla*_NDM-1_) [82].

AMR poses a severe threat to global economic growth. Projections indicate it could reduce global Gross Domestic Product (GDP) by up to 3% by 2030 and by 1.1% to 3.8% by 2050 [82,146]. Another study suggests the global GDP decline by 2050 could range between 2% and 3.5% [165]. This near-term impact would coincide with an additional $700 billion in global healthcare expenditures that year, a burden disproportionately borne by low-income countries [82,165,166]. Long-term cumulative costs could reach up to $100 trillion [165]. The financial repercussions are significant at regional levels as well, as highlighted by a European Centre for Disease Prevention and Control (ECDC) report citing approximately €1.5 billion in annual additional patient care expenses due to AMR-related infections [167]. The financial strain arises from the complexities of managing drug-resistant infections, which necessitate longer hospital stays, more expensive alternative drugs, increased morbidity, and reduced productivity [168]. Concerns are mounting regarding the escalating financial burden of treating MDR infections. This escalating burden risks exacerbating poverty and hindering progress toward the Sustainable Development Goals. Addressing this complex threat requires an urgent and effective implementation of the One Health approach, recognizing the inextricable links between human, animal, and ecosystem health [169].

### 5.2. The Resistance Profile of P. mirabilis: An Overview

The global state of antimicrobial resistance (AMR) in *P. mirabilis* is both dynamic and alarming, with nearly 48% of strains exhibiting antibiotic resistance [170]. This pathogen accumulates additional antimicrobial resistance genes (ARGs) via horizontal gene transfer, driving the development of multidrug-resistant (MDR) and extensively drug-resistant (XDR) strains [95]. Key ARGs facilitating this propagation include *bla*_TEM_, *bla*_CTX-M_, *bla*_KPC_, *bla*_NDM_, *bla*_VIM_, and *mcr*-1 [171]. *P. mirabilis* possesses a formidable resistance profile encompassing both innate and acquired mechanisms. It is intrinsically resistant to nitrofurans, polymyxins (including colistin), tigecycline, and tetracycline [172], and has developed acquired resistance to numerous other classes including trimethoprim/sulfamethoxazole, aminoglycosides, carbapenems, fluoroquinolones, β-lactams, imipenem, cephalosporins, penicillins, and aztreonam [30,170]. This adaptability is bolstered by its role as an effective ARG reservoir [173].

Crucially, β-lactam resistance is mediated by the synthesis of ESBLs and ampC β-lactamases, which hydrolyze penicillins, cephalosporins, and aztreonam [174]. ESBL-producing isolates frequently exhibit co-resistance to other classes, such as quinolones and aminoglycosides [175]. Furthermore, chromosomal mutations can lead to phenotypic resistance to all β-lactam drugs [170]. Genomic studies confirm a wide array of resistance genes [176], with increasing reports of strains harboring multiple ARGs, including those for quinolones (e.g., *qnr*, *aac(6′)-Ib*) and aminoglycosides (e.g., *APH*, *AAC*, *AAD*) [170].

Surveillance data reveal a severe and complex resistance landscape with significant geographical and host-based variation. Isolates from China harbor the highest ARG counts, while those from the United States contain the fewest [95]. Animal-derived isolates consistently possess more ARGs than human clinical isolates, with urine isolates showing the greatest ARG diversity among human samples [95]. This interconnectivity is highlighted by the presence of similar ARGs in raw meat, migratory birds, and human clinical samples [177]. The rate of amoxicillin-resistant *P. mirabilis* (38–48.5%) in medical institutions parallels that of *E. coli* [170,178], and it ranks as the second most prevalent ESBL-producing *Enterobacteriaceae* in poultry [179]. This widespread resistance stems from the organism’s high genomic diversity, driven by mobile genetic elements, mutations, and genomic rearrangements, which yield highly diverse virulence and resistance factors even among isolates from the same source [180,181,182]. Such diversity poses a significant challenge to diagnosis, infection control, and treatment [183,184]. The genetic basis of this resistance is further elucidated by the wide array of antibiotic resistance genes (ARGs) identified in *P. mirabilis* isolates from diverse animal reservoirs. Surveillance studies have mapped specific ARGs to their animal sources, revealing a complex transmission network [86]. More concerning is the identification of genes conferring resistance to last-resort or critically important antibiotics. The polymyxin resistance gene *mcr*-1 has been reported specifically in poultry isolates [57]. The carbapenemase gene *bla*_KPC_ has been found in farm animals [52,55]. The fosfomycin resistance gene *fos*A3 has been identified in livestock and food products [53,61,185], though its distribution shows geographical variation; for instance, recent surveillance in Egyptian livestock (buffaloes and broiler chickens) reported an absence (*fos*A3-negative) of this gene in these populations [79]. This detailed genotypic landscape underscores how animal reservoirs serve as melting pots for the accumulation and dissemination of ARGs, with clear implications for zoonotic transmission and food safety.

The emergence and circulation of resistant *P. mirabilis* in animals and the food chain pose a major zoonotic and foodborne threat, epitomizing a One Health challenge. Zoonotic transfer is facilitated by the inseparable interconnectivity of humans, animals, and shared environments, enabling constant exchange of resistant organisms and their genetic elements [186]. While zoonotic pathogens are linked to nearly two-thirds of major recent infectious disease outbreaks, successful cross-species transmission depends on a complex interplay of ecological and genetic factors [186].

Transmission occurs via interconnected environmental and direct routes. Resistant strains from livestock operations enter the environment through wastewater effluent and agricultural runoff, contaminating water and crops and creating secondary exposure routes that hinder containment [187]. Supporting this, a scoping review that analyzed 70 out of 3367 identified studies on transmission pathways concluded that antibiotic residues, heavy metals, and microbial interactions in wastewater are key drivers of AMR. It also noted that wastewater treatment plants (WWTPs), while designed to reduce contaminants, can create conditions favoring horizontal gene transfer, thereby amplifying resistance genes [188]. This environmental pathway, often involving untreated waste, plays a significant role in accelerating the AMR pandemic and underscores the critical need for enhanced vigilance regarding the environmental dimension of infectious diseases, a lesson reinforced by the COVID-19 pandemic [189].

Human infection occurs through multiple pathways, including direct contact with infected animals (a major occupational risk for farm workers), contact with contaminated surfaces, water, or soil, and—importantly—ingestion of contaminated animal products [80]. The contamination of poultry and meat products is a particularly effective route for introducing virulent, resistant strains into the human food chain [80]. An alarming scenario is the potential for meat from broiler chickens or buffalo carrying PDR *P. mirabilis*—non-susceptible to all antimicrobial categories—to act as a direct reservoir for transmitting untreatable strains to humans, causing severe, therapy-limited infections like complicated urinary tract infections [79]. The emergence of PDR strains represents the final, most critical stage in the resistance continuum (MDR → XDR → PDR). Genomic evidence showing strong relatedness between resistant animal and human clinical isolates confirms a common origin and transmission mechanism [79].

Surveillance has identified major livestock as key reservoirs of resistant *P. mirabilis*, including cattle [190,191,192,193], buffalo [79,194,195,196,197], pigs [198,199], poultry [78,79,81,200,201,202], ducks [78,202], and dogs [58,59,203,204,205]. The risk to human populations is disproportionately higher in low- and middle-income countries due to inequities in food safety standards, regulatory enforcement, and healthcare access. The persistent detection of clinically relevant resistant *P. mirabilis* in food animals and retail meat underscores its potent zoonotic threat and the non-negotiable need for a unified, cross-sectoral One Health strategy to manage its spread [95].

The rising tide of MDR strains necessitates a coordinated One Health response. Despite advances, significant gaps remain in data on AMR from animal and environmental sources and their transmission mechanisms [206]. The predominance of MDR strains, which outcompete first-line therapies, highlights the urgent need for novel antibiotic targets and illustrates the profound clinical and public health consequences of AMR in *P. mirabilis*. Modelling suggests that reducing human antibiotic consumption could decrease resistant colonization by 65.7–99.7% over two decades [207], underscoring the need for improved infection control in both healthcare and agriculture [208]. Effective AMR management requires interdisciplinary collaboration, integrated surveillance, and antimicrobial stewardship across all sectors.

Recent large-scale genomic surveillance of 3403 high-quality genomes from 58 countries confirms that human clinical isolates (especially from UTIs) are the primary resistance reservoir [95]. This study identified a vast repertoire of 239 distinct ARGs, with β-lactamase and carbapenemase genes being exceptionally widespread, and reaffirmed the highest ARG burden in Chinese isolates [95]. Phylogenetic analysis grouped global isolates into 17 clusters, with U.S. isolates showing the widest spread. The minimal genetic variation between isolates from different countries and hosts strongly suggests transnational and cross-host clonal spread, reinforcing the interconnected nature of AMR within the One Health framework [95]. A comprehensive understanding of this escalating threat necessitates a deeper examination of the specific molecular mechanisms empowering *P. mirabilis* across human, animal, and environmental settings.

### 5.3. Molecular Mechanisms of Antimicrobial Resistance in P. mirabilis: A Unifying Framework

**Figure 3:**
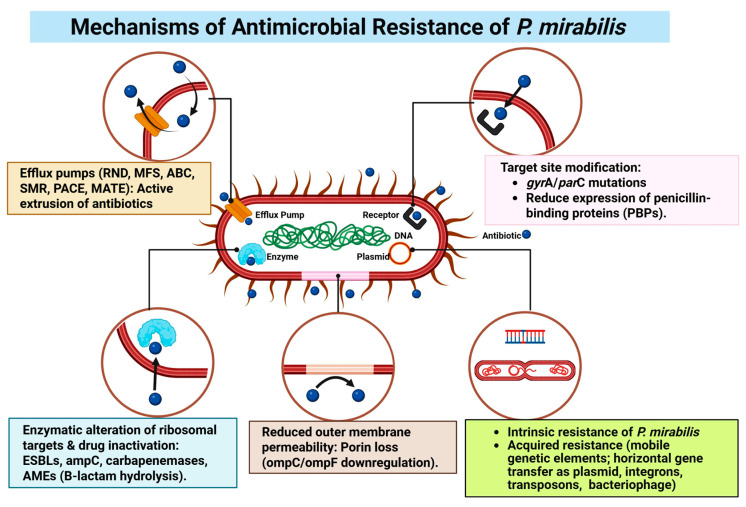
Key mechanisms of antimicrobial resistance in *P. mirabilis*. The schematic summarizes the multifactorial resistance strategies employed by *P. mirabilis*, encompassing both intrinsic traits and acquired genetic elements. Major mechanisms include: active antibiotic extrusion via efflux pumps (e.g., RND, MFS); enzymatic drug inactivation or modification through β-lactamases (ESBLs, ampC, carbapenemases) and aminoglycoside-modifying enzymes (AMEs); reduced outer membrane permeability via porin loss (*omp*C/*omp*F downregulation); and target site modifications, such as mutations in *gyr*A/*par*C or altered expression of penicillin-binding proteins (PBPs). Acquired resistance is frequently mediated by mobile genetic elements (plasmids, integrons, transposons) through horizontal gene transfer, amplifying the spread of multidrug-resistant phenotypes. The schematic integrates intrinsic and acquired resistance strategies. Large rectangles represent major mechanistic categories (efflux pumps, enzymatic alteration, reduced outer membrane permeability, target site modification). Smaller nested rectangles specify sub‑mechanisms. Solid black arrows denote direct mechanistic flow or causal relationships; dashed arrows indicate downregulation or reduced expression (e.g., porin loss, PBP reduction). Abbreviations: RND, Resistance‑Nodulation‑Division; MFS, Major Facilitator Superfamily; ABC, ATP‑Binding Cassette; SMR, Small Multidrug Resistance; PACE, Proteobacterial Antimicrobial Compound Efflux; MATE, Multidrug and Toxic Compound Extrusion; ESBL, Extended‑Spectrum β‑Lactamase; AME, Aminoglycoside‑Modifying Enzyme; PBP, Penicillin‑Binding Protein.

To overcome antimicrobial compounds, bacteria employ several fundamental molecular strategies. These include modifying the target site to prevent the antimicrobial from binding, inactivating or reducing the compound to ineffective levels through enzymatic degradation, actively expelling the antimicrobial via efflux pumps, and reducing cellular uptake of the compound, typically achieved through an impermeable outer membrane [209,210,211] (Figure 3). *P. mirabilis* belongs to GNB, has an intricate cell envelope structure composed of a thin layer of peptidoglycan. More importantly, it has an outer membrane rich in lipopolysaccharides. This renders it strong biological armor, making it difficult for antibiotics to penetrate [212].

Horizontal gene transfer (HGT) through mobile genetic elements (MGEs) helps in the dissemination of resistance gene traits among and within bacterial populations after they emerge. These MGEs, which include plasmids, transposons, and integrons, essentially serve as vectors carrying the swift dissemination of ARGs among various species of bacteria in the hospital and community settings [40,170,213,214]. This transfer can occur via mutational alterations or direct interbacterial contact, resulting in treatment failure and resistance growth [215]. Alternatively, the effective resistant strains’ expanding clones propagates these determinants [216]. Interestingly, MGE mobilization and resistance co-selection can be caused by various environmental stresses in addition to antimicrobials. Exposure to heavy metals, oxidative stress, or ultraviolet light might cause the transfer of genetic elements, leading in the selection of ARGs alongside genes giving resistance to these non-antibiotic hazards [217,218]. Antimicrobial resistance essentially occurs when a bacterium develops a physiological adaptation to counteract the effects of a medication, either by structural alterations, enzyme inactivation, or other mechanisms [212]. These skills fall into two main categories that are relevant to *P. mirabilis*: acquired resistance, which is obtained by genetic mutation or the horizontal acquisition of foreign resistance genes, and intrinsic (inherited) resistance, which is a natural trait of the species or genus.

#### 5.3.1. Intrinsic Resistance in *P. mirabilis*

Intrinsic resistance is the most fundamental and intrinsic kind of bacterial resistance to antimicrobial agents. In *P. mirabilis*, non-acquired resistance is a species-specific feature resulting from spontaneous mutations or innate physiological traits rather than horizontal gene transfer. It originates from processes such as the absence of a drug’s target, low-affinity target locations, an impermeable cellular envelope, or the natural generation of inactivating enzymes and can emerge even in the absence of direct antimicrobial pressure [219]. One distinguishing feature of *P. mirabilis* is its innate resistance to specific antibiotic groups. Most importantly, the species is naturally resistant to polymyxin drugs like colistin [170,220]. The fundamental mechanism of resistance is structural changes to the lipopolysaccharide (LPS) in the outer membrane. These changes diminish the net negative charge on the bacterial cell surface, preventing positively charged polymyxin molecules from initially attaching electrostatically. This intrinsic characteristic substantially limits the treatment choices for infections caused by multidrug-resistant *P. mirabilis*.

Furthermore, *P. mirabilis* frequently shows decreased susceptibility to the carbapenem antibiotic imipenem [18,220]. While not completely resistant, more significant resistance can emerge due to the loss of outer membrane porins (e.g., *omp*C, *omp*F) or reduced expression of certain penicillin-binding proteins (PBPs), especially PBP1a and PBP2. These modifications restrict medication inflow or lower target binding affinity. *P. mirabilis* is missing natively manufactured chromosomal β-lactamases; hence, any resistance to β-lactam antibiotics is acquired rather than innate. Aside from these specific examples, intrinsic resistance in *P. mirabilis* and kindred species can be linked to a variety of constitutive variables, such as efflux pump activity that is always low and the presence of protective proteins. Natural resistance to antimicrobial classes, including β-lactams, aminoglycosides, and fluoroquinolones, is linked to the function of various genes, such as ampC, *bla*_SHV_, *trx*A (thioredoxin), and *trx*B (thioredoxin reductase), among others [221]. A thorough understanding of these internal mechanisms is essential for establishing effective therapeutic options and countermeasures to combat the growing threat of resistant *P. mirabilis* strains.

#### 5.3.2. Acquired Resistance and Horizontal Gene Transfer in *P. mirabilis*

Acquired resistance is the key clinical difficulty in managing *P. mirabilis* infections, owing to the bacterium’s remarkable ability to get resistance genes against a wide range of essential antibiotics, including beta-lactams, fluoroquinolones, and aminoglycosides. Acquired resistance occurs when previously sensitive bacteria gain the ability to resist an antimicrobial agent.

In prokaryotes, lateral gene transfer, or more recently, lateral genetic transfer (LGT), is a crucial mechanism for transferring and rearranging DNA [222]. Based on reports, up to 25% of some bacterial genomes may have originated from LGT during evolutionary periods [223], demonstrating the scope of LGT. The implications of LGT for human health are significant. In fact, some argue that humans are losing the battle against antibiotics and antibiotic resistance [224]. This happens through the acquisition of ARGs via two major pathways: vertical gene transfer (inheritance from a parent cell to its offspring) and, more importantly, HGT. These processes can occur concurrently, but HGT is particularly important because it allows for the direct exchange of genetic material between modern cells, introducing entirely new resistance genes and mechanisms into a bacterial population and leading to improved collective resistance profiles. HGT is a major driver of antimicrobial resistance spread and operates through three primary mechanisms, each contributing differently to resistance dissemination across the human–animal–environment interface, as summarized in Table 2. HGT is a main driver of AMR spread and can occur through three primary mechanisms: transformation (free DNA uptake), transduction (bacteriophage transfer), and conjugation (direct cell-to-cell transfer via plasmids or other conjugative components).

The ability to transfer genes between species accelerates the spread of resistance. Furthermore, it is worth noting that exposure to various physical or chemical stimuli has the potential to cause selected mutations in bacterial DNA, which contribute to resistance development. Different types of bacteria possess different potential applications of acquired resistance mechanisms due to their intrinsic structural differences. For example, GNB like *P. mirabilis* can possess all four major types of acquired mechanisms: target-site alterations, enzyme inactivation of drugs, active pumping of out-drugging, and prevention of drug uptake. The complex nature of the outer cell envelope, which includes an outer membrane composed of LPS, and also contributes to the ability of GNB to adapt. On the other hand, Gram-positive bacteria do not have this outer membrane, and therefore, they do not rely on the same mechanisms to restrict drug uptake [212]. To comprehend the spread of both currently known and future discovered resistance genes, it is essential to comprehend the genetic components of each type of resistance mechanism, the nature of their life cycle, the dynamics of LTG, and the ecological context in which these mechanisms develop [225]. The next sections explore acquired resistance mechanisms of *P. mirabilis*.

#### 5.3.3. Key Vehicles of Horizontal Gene Transfer

##### Mobile Genetic Elements as Vectors of Antimicrobial Resistance in *P. mirabilis*

MGEs are critical factors in the acquisition and spread of ARGs in *P. mirabilis* populations. These mobile DNA segments serve as excellent transporters for the horizontal transmission of resistance determinants across bacterial cells, contributing significantly to the rapid development and extensive dispersion of MDR strains. The primary kinds of MGEs involved in this process include many major sorts.

##### Plasmids and Integrative Conjugative Elements in AMR Dissemination

An important discovery is that *P. mirabilis* has integrative and conjugative elements (ICEs), such as ICEPm1, which contains both virulence and antibiotic resistance genes. These elements can replicate independently and self-transfer to other strains and even different bacterial species, making them effective agents of genetic exchange [18]. *P. mirabilis* has become a serious public health problem due to its strong combination of virulence and high resistance levels. Plasmids are a type of MGEs. They are self-replicating, circular pieces of DNA that exist outside the chromosome and can hold many different types of antibiotic resistance genes. Plasmids allow the rapid transfer of these resistance genes between bacterial cells by conjugation (sending genetic material from one cell to another). Conjugation not only allows for the rapid spread of resistance genes among bacteria of the same species, but also requires very little time to occur; therefore, plasmid-based resistance can spread quickly from one species to another. In the case of *P. mirabilis*, the plasmid-carrying resistance genes include ESBLs, ampC β-lactamases and carbapenemases, thus allowing for a rapid and widespread dissemination of these resistance types [220].

On plasmids, key resistant genes including *bla*_CTX-M-65_, *bla*_CTX-M-2_ and *bla*_CMY-2_ have been detected in a range of sources, including meat products and clinical samples [95]. These resistant genes frequently co-occur within certain recognized plasmid incompatibility groups (e.g., IncT, IncW, IncFIA, IncFIB and IncK) [170]. Recent field evidence powerfully illustrates this dynamic: a study of pig-derived *P. mirabilis* in China found that 50% of multidrug-resistant isolates carried the IncQ1α plasmid, identified by the *rep*C gene. Whole-genome sequencing revealed these IncQ1α plasmids carried between 33 and 38 diverse resistance genes. Notably, these IncQ1α-positive isolates also co-harbored structural genes from F-type plasmids (e.g., *tra* operon genes), exhibiting 48 distinct structural patterns with no apparent regularity, highlighting the complex and adaptive nature of plasmid interactions in animal reservoirs [85].

In addition to the aforementioned plasmid types, some resistance genes have been observed in variations (for example, CTX-M-65) within Tn7-like composite transposons on plasmids associated with highly drug-resistant phenotypes [226]. Plasmids and ICEs are two separate means by which ARGs are generally found in *P. mirabilis* in comparison with the alternate method of dissemination of AMRs via vertical genetic transmission (i.e., through cell division) [95]. In addition, the original discovery of an ICE containing a significant number of ARGs was made in *P. mirabilis* [227]. A very large distribution of STX/R391 ICE families contribute primarily to the resistance profile of this pathogen. As the recent spread of the *tmexCD-toprJ* gene complex encoding the efflux pump via ICEs indicates, these elements should be closely monitored [228].

The predominant form of MGEs found in *P. mirabilis* is the Insertion Sequence (IS) family—the predominant ISs that are detected most frequently are members of the IS200/IS605 family. In comparison with isolates from humans, animal isolates (e.g., from chickens and dairy cattle) contain a much greater variety and a much more diverse array of MGEs, including ISPpu12, IS26, Tn7, and Tn2 in animal isolates. It seems likely, therefore, that the increased frequency of drug resistance among *P. mirabilis* is ascribed primarily to the presence of ISs, plasmids, and ICEs, and future research is warranted [229]. *P. mirabilis* exhibits a clonal propagation distribution for ICEs/ISs/plasmid replicons vs. horizontal transfer of prophages, according to the results of ancestral state reconstruction. This distribution is similar to that observed in *Klebsiella pneumoniae (K. pneumoniae)* [229]. Therefore, the increased prevalence of multidrug resistance in *P. mirabilis* is largely attributable to the dynamic interplay of IS elements, plasmids, and ICEs, warranting continued research within a One Health framework.

##### Integrons as Key Mediators of Antimicrobial Resistance Gene Acquisition in *P. mirabilis*

Integrons are specialized genetic elements that act as natural systems for collecting, integrating, and expressing gene cassettes, notably those containing antimicrobial resistance genes (ARGs). They constitute a highly efficient mechanism enabling bacteria to rapidly acquire new resistance traits [19]. Functionally, integrons enhance bacterial adaptability by capturing short, mobile gene cassettes and incorporating them at a specific integration site (*att*L) via a site-specific recombinase (integrase, *int*I), ensuring the efficient expression and dissemination of acquired genes [230,231,232]. Although integrons themselves are not directly mobile, they are frequently located on plasmids and transposons; this association allows them to assemble multiple resistance genes into a single, easily transferable unit, thereby playing a crucial role in horizontal gene transfer and bacterial evolution [19].

Integrons are commonly found in the *Proteobacteria* phylum and can be chromosomal or mobile [233,234]. Their importance in clinical settings stems from their ability to associate with other MGEs, such as plasmids and transposons, facilitating the widespread dissemination of resistance determinants [19]. Transposons, for instance, are MGEs that move genes between DNA molecules. They often carry single or multiple ARGs—such as tetracycline (Tn10), ampicillin (Tn3), or chloramphenicol (Tn9) resistance—and can combine with integrons to form complex genetic structures (e.g., Tn21) that further amplify resistance potential [235,236]. In *P. mirabilis*, the acquisition of ARGs is heavily mediated by these mobile genetic platforms. Integrons specifically assist in transferring cassettes harboring genes responsible for resistance to β-lactams, aminoglycosides, and plasmid-mediated quinolone resistance (PMQR) into recipient cells, significantly contributing to the emergence of MDR phenotypes [170].

Integrons are categorized into several classes based on integrase gene sequences, with Class 1 integrons being most strongly associated with MDR in clinical isolates [181,237]. While other classes (e.g., Class 2 and Class 3) have been identified on mobile elements, their impact appears more restricted [238,239]. These integron systems are key vectors for numerous antibiotic resistance determinants found in *Enterobacteriaceae*, including *P. mirabilis* [240]. Notably, complex integrons can serve as genetic platforms for specific plasmid-mediated beta-lactamases and PMQR genes. These structures may incorporate elements like insertion sequence common region 1 (ISCR1) and amplify resistance by duplicating the 3′-conserved segment (3′-CS), thereby expanding the variable repertoire of resistance gene cassettes located between the 5′ and 3′ conserved regions [241]. The presence and activity of integrons, therefore, are strongly correlated with elevated antibiotic resistance rates in *P. mirabilis*, underscoring their pivotal role in the global AMR crisis [19].

##### Role of Transposons and Bacteriophages in AMR Gene Mobility

Transposons, sometimes known as “jumping genes,” are mobile DNA sequences that can relocate genetic material, including ARGs, inside a bacterial genome or between various genetic units like plasmids and the chromosome. These elements usually contain integrons or individual resistance genes, which play an important role in mobilizing and distributing resistance determinants [220]. Transposases are considered the most common genes in nature [205].

Another, but more complicated, method of gene transfer is bacteriophage-mediated transduction. According to recent studies, prophage sequences incorporated into bacterial genomes can contain a considerable number of ARGs by phage transduction, a process by which bacterial viruses (bacteriophages) transfer genetic material [242]. *P. mirabilis* is a host for a variety of bacteriophages as it belongs to the *Enterobacterales* order. However, the precise function and epidemiological impact of these *P. mirabilis*-infecting phages in the active spread of ARGs are still unclear; available data indicates that their role may be less significant than that of plasmids or transposons in this particular pathogen [95]. Despite this possible secondary role, the presence of ARGs among prophages shows that *P. mirabilis* could act as a reservoir, with phages contributing to gene pool mobilization throughout microbial communities [95]. In parallel to their potential role in gene transfer, contemporary research in phage therapy highlights the therapeutic potential of phages to combat the same resistant infections. Despite the wide host range of *Proteus* for phages, the overall diversity of characterized *Proteus*-infecting phages remains remarkably low, with only 61 isolates submitted to public databases such as GenBank as of 2025, indicating critical environmental under sampling [243]. This gap limits our full understanding of their epidemiological and therapeutic roles. The newly discovered podovirus Premi, belonging to the Autographiviridae family, represents a valuable addition to this limited repertoire [244]. It is characterized by a lytic cycle and a genome devoid of virulence or toxin genes, which makes it a promising candidate for therapeutic applications, particularly against urinary tract infections [244]. However, its narrow host range—demonstrating activity against only 3 out of 30 tested clinical isolates—underscores the necessity for broader discovery efforts to develop comprehensive therapeutic cocktails [244]. Consequently, there is a pressing need for a larger collection of characterized *Proteus* phages to improve our capability to develop effective phage-based therapeutics [244]. The wide diversity of MGEs such as bacteriophages and transposons, among others, found in populations of *P. mirabilis* allows for the rapid movement and sharing of ARGs. The genetic similarity of *P. mirabilis* from different sources, such as chicken meat and humans with urinary tract infections, also proves that MGEs are important in allowing resistant clones to be spread between hosts and around the world [95].

### 5.4. Core Resistance Mechanisms

#### 5.4.1. The Role of Efflux Pumps in Multidrug Resistance of *P. mirabilis*

Within *P. mirabilis*, the efflux pump is an integral membrane transporter that play a significant mechanism of bacterial pathogenesis, metabolism, and multidrug resistance [182,245,246]. The active removal of many types of antibiotic agents from the bacterial cell, through specialized proteins located across the plasma membrane, decreases the internal concentration of the antibiotic to sub-therapeutic levels [183]. Consequently, the efflux pump prevents the antimicrobial agent from exerting any bactericidal or bacteriostatic action, by removing the agent rapidly and maintaining the intracellular level of the agent below the minimum inhibitory concentration (MIC). As a result, efflux transporters have become promising targets for the development of new inhibitors to combat MDR-associated infectious diseases [247].

Several antibiotic families, including fluoroquinolone, tetracycline, aminoglycosides and some beta-lactams, show a link between efflux pump activity and resistance in *P. mirabilis* [248]. Overexpression or hyperactivation of the efflux pumps leads to a significant increase in the MDR phenotype of clinical isolates. While efflux pumps in *P. mirabilis* may not have been studied as extensively as beta-lactamase enzymes [220], they play a critical role in conferring resistance to antibiotics. Interestingly, some antimicrobials can function as specific substrates for these specialized efflux mechanisms, thus confirming their focused role in drug resistance [209].

These efflux systems are categorized into several major superfamilies based on their sequence homology, substrate specificity, structural features, energy sources, and efflux transporters. The most clinically significant families include the Resistance-Nodulation-Division (RND), Major Facilitator Superfamily (MFS), ATP-Binding Cassette (ABC), Small Multidrug Resistance (SMR), Proteobacterial Antimicrobial Compound Efflux (PACE), and Multidrug and Toxic Compound Extrusion (MATE) families [249,250,251].

ABC transporters play a dual role in antibiotic resistance and virulence. Export systems actively pump out antibiotics, as seen in the MacAB-TolC system. A tripartite efflux system consists of MacA (membrane fusion protein), MacB (ATP binding cassette transporter), and TolC (outer membrane channel), which contributes to macrolide resistance and virulence factor secretion in *Enterobacteriaceae* [252,253]. Current research aims to develop ABC transporter inhibitors as antibiotic adjuvants, though challenges remain due to the redundancy and complex regulation of bacterial efflux systems [254].

The major facilitator superfamily (MFS) is the largest and most extensively characterized group of transmembrane secondary transport proteins in both prokaryotic and eukaryotic systems [255]. This efflux mechanism reduces intracellular antibiotic concentrations, thereby preventing the drug from reaching its biological targets [256].

Functionally, SMR proteins play a critical role in the synthesis and efflux of various lipophilic compounds, including antiseptics, detergents, antibiotics, and other drugs. The SMR family encompasses a diverse group of proteins encoded by genes located on both plasmids and bacterial chromosomes, demonstrating substantial structural and functional heterogeneity. This diversity enables SMR proteins to resist different classes of antibiotics, including β-lactams such as cephalosporins. The genetic basis for this resistance is at tributed to the close association between SMR genes and antimicrobial resistance (AMR) genes within bacterial genomes, thereby enhancing the multidrug resistance capabilities of bacterial cells [257].

MATE transporters are secondary active transporters, primarily involved in the efflux of cationic substrates, and play a critical role in reducing bacterial susceptibility to a range of antimicrobial agents. These include ethidium bromide, berberine, acriflavin, norfloxacin, and tetraphenylphosphonium, all of which are substrates that can accumulate to toxic levels within bacterial cells if not extruded by these pumps [258].

First identified in 2013, PACE family transporters have been found predominantly in *Proteobacteria*, particularly in clinically relevant pathogens [259], transport proteins that contribute to antimicrobial resistance. The discovery of PACE family transporters has significant implications for public health and clinical practice. Their ability to confer resistance to commonly used biocides like chlorhexidine poses challenges for infection control in healthcare settings [260]. Understanding the structure and function of PACE transporters could lead to the development of novel inhibitors, potentially restoring the efficacy of certain antimicrobials [261].

Resistance Nodulation Cell Division (RND) Superfamily pumps are associated with outer membrane proteins (OMPs) and are facilitated by periplasmic adaptor proteins (PAPs) [250,252].

#### 5.4.2. Target Site Modification as a Mechanism of Antimicrobial Resistance

##### General Principles of Target Site Alteration

Apart from mechanisms such as enzymatic drug inactivation and efflux pumps, *P. mirabilis* can develop resistance due to structural changes in the cellular targets that antibiotics are designed to block [262]. Spontaneous chromosomal mutations can affect the genes encoding these essential target proteins, which are typically involved in important processes, including cell wall synthesis and DNA replication. Such genetic changes can greatly reduce the antibacterial agent’s binding affinity and thus its therapeutic effectiveness [262]. Because the mode of action for many antibacterial agents relies heavily on very specific molecular interactions, even small structural alterations to the target site may cause large disruptions to this binding [209]. An example of how mutations can lead to fluoroquinolone resistance would be shown in the bacteria *P. mirabilis* where mutations commonly occur in the genes for the two main targets of fluoroquinolones which are the enzymes termed DNA gyrase (*gyr*A and *gyr*B), and topoisomerase IV (*par*C and *par*E). These 2 types of enzymes are the principal sites of action for fluoroquinolones. Therefore, when a mutation occurs in either of these genes, the structure of the enzyme at the point where the fluoroquinolone binds will change which results in an inability of the medication to bind to its target or, in turn, to neutralize the antimicrobial properties of the fluoroquinolone [179].

Strains of *P. mirabilis* exhibit a lower susceptibility to other antibiotic families, specifically to β-lactams [262], that demonstrates a level of target-based resistance that extends beyond fluoroquinolones. This accumulation of target site mutations results in greater antimicrobial resistance challenges presented by this opportunistic pathogen, complicating potential treatments, and encouraging the emergence of multidrug-resistant strains that are increasingly difficult to manage.

#### 5.4.3. Enzymatic Alteration of Ribosomal Targets and Drug Inactivation

*P. mirabilis* can develop resistance to aminoglycoside drugs by modifying its bacterial target enzymatically. Modifications to ribosomal RNA (rRNA) or ribosomal proteins can inhibit aminoglycoside molecules from binding effectively, resulting in resistance [262]. This technique is one component of enzymatic drug inactivation, which is carried out by numerous major enzyme families. β-lactamases, aminoglycoside-modifying enzymes (AMEs), and chloramphenicol acetyltransferases are the three main categories of these inactivating enzymes. By hydrolyzing the core β-lactam ring of penicillins, cephalosporins, monobactams, and carbapenems, β-lactamases impart resistance. AMEs, on the other hand, offer broad-spectrum resistance by chemically altering the aminoglycoside drug molecule itself, which lowers the drug’s overall affinity for the target and stops it from binding to the 30S ribosomal subunit [209].

#### 5.4.4. Reduced Outer Membrane Permeability and Porin Loss

The outer membrane of GNB, such as *P. mirabilis*, serves as a main defense against a variety of antimicrobial drugs. Porins, specialized outer membrane proteins that create channels filled with water, help break through this barrier. Numerous antibiotics and other hydrophilic compounds can passively diffuse into the periplasmic region through these channels. A crucial resistance mechanism is the loss or reduction in expression of these porins. This decrease in porin abundance lowers outer membrane permeability, effectively inhibiting antibiotic entrance and preventing medicines from reaching their intracellular targets [220]. This technique is especially important in cases of β-lactam resistance. For carbapenems, the loss of key porins such as *omp*C and *omp*F can result in markedly reduced susceptibility or even clearly apparent clinical resistance [220].

### 5.5. Epidemiology of Resistance to Key Drug Classes in P. mirabilis

#### 5.5.1. β-Lactam and Cephalosporin Resistance: The Dominant Threat

Cephalosporins are commonly used in clinical settings to treat respiratory, urinary tract, and gastrointestinal infections. This extensive use places significant selection pressure on members of the *Enterobacteriaceae* family, resulting in resistance [240]. The 2019 surveillance research demonstrated this concern, showing that 8.4% of *P. mirabilis* isolates were resistant to a variety of medicines, including ciprofloxacin, amoxicillin, gentamicin, amoxicillin/clavulanate, and cefotaxime. Within this resistant subset, genotypic investigation revealed that 28.6% carried the ESBL gene *bla*_CTX-M-2_, whereas the majority—71.4%—had a combination of the ampC gene *bla*_CMY-2_ and the ESBL gene *bla*_TEM-1_ [263]. Despite these resistance trends, several antibiotics remain highly effective. For example, ceftazidime, piperacillin-tazobactam, and meropenem inhibited more than 98.0% of clinical *P. mirabilis* strains collected from US hospitals between 2011 and 2013 [264]. The detection of identical ESBL and plasmid-mediated ampC (pAmpC) genes in human clinical isolates, livestock, and retail meat underscores the interconnectedness of resistance dissemination, reinforcing the need for integrated surveillance across the One Health spectrum.

Resistance is caused by either overproduction of chromosomal ampC β-lactamases or the acquisition of transferable ESBLs [265]. Other contributing processes include variations in outer membrane porins and proteins that impair antibiotic permeability [266].

*Proteus* spp. Have acquired virtually the entire arsenal of β-lactamases known in the *Enterobacterales* order. A principal resistance mechanism in *P. mirabilis* is the production of these enzymes, especially ESBLs, which hydrolyze the β-lactam ring and inactivate penicillins and cephalosporins [18,180,267]. Although the chromosome of *P. mirabilis* does not harbor intrinsic β-lactamase genes, the bacterium can produce a wide variety of enzymes, including narrow spectrum penicillinases, ESBLs, acquired ampC cephalosporinases, and carbapenemases [170,268]. ESBLs are plasmid mediated enzymes that confer resistance to penicillins, first through third generation cephalosporins, and aztreonam, although their activity is typically inhibited by clavulanic acid. The location of ESBL genes on mobile genetic elements has facilitated their rapid spread within *P. mirabilis* populations and to other species, posing a major clinical challenge [19,184]. For instance, there have been two comprehensive reviews on genes for β-lactamases, dealing with their diversity, mobility and epidemiology [269,270].

The range of β-lactamases detected in *P. mirabilis* includes narrow spectrum enzymes (e.g., TEM, SHV, CARB, and IRT derivatives), acquired cephalosporinases (e.g., DHA, CMY, ACC-1), ESBL types (e.g., TEM/SHV, CTX-M, VEB, PER), and carbapenemases [174]. ESBL production typically results in high level resistance to ceftazidime, cefotaxime, and aztreonam [170]. The first documented case of ESBL mediated resistance in *Proteus* spp. Dates to 1987 [271]. A 2020 study found that 37% of strains produced ESBLs, all carrying the *bla*_TEM_ gene, although these isolates remained susceptible to cefotaxime/clavulanic acid, cefoxitin, and imipenem [272]. However, recent data from high-risk reservoirs challenge this notion. A 2025 study revealed a widespread β-lactamase genotype among extensively drug-resistant *P. mirabilis* isolates, with the *bla*_TEM_ gene detected in 97.06% of poultry and 81.82% of buffalo isolates. The ESBL gene *bla*_CTX-M_ was present in 26.47% and 18.18%, and the ampC gene *bla*_CMY-2_ in 41.18% and 63.64% of isolates, respectively. Critically, these genotypic patterns were linked to a phenotypic profile of carbapenem non-susceptibility, indicating that the pervasive carriage of such genes in livestock production environments can be associated with the emergence of resistance to last-resort agents, even in the absence of traditional carbapenemase genes [79].

Class A broad spectrum β-lactamases in Proteus species include TEM-1, TEM-2, and SHV-1, which are inhibited by clavulanic acid and confer resistance to ampicillin and amoxicillin [170]. Oxacillinases (OXA) such as OXA-1, OXA-9, OXA-10, and the more recent OXA-320 (associated with integrons and the aminoglycoside resistance gene *aad*A1) have been reported, primarily from Turkey; these are not effectively inhibited by clavulanic acid, sulbactam, or tazobactam [273].

Early studies identified TEM-52 as the predominant ESBL conferring resistance to extended spectrum cephalosporins in *P. mirabilis* [274,275]. Most TEM variants are ceftazidimases, while SHV variants are less frequent than TEM and CTX-M types [276]. A shift from TEM/SHV to CTX-M variants became apparent in the early 2010s. Predominant CTX-M enzymes in *P. mirabilis* include CTX-M-2, -3, -14, and -27 [170,277]. CTX-M-8 is rare, having been found only in Brazil, while CTX-M-65 has been identified in Russia [226]. The *bla*_CTX-M-15_ gene is often preceded by ISEcp1, which mobilizes the gene and elevates its expression, leading to high level cephalosporin resistance [278]. CTX-M-14 is the most widely distributed variant in *Proteus* spp., first reported in South Korea [178] and later in China alongside its reduced activity derivative CTX-M-140 [279]. Notably, the presence of ESBL genes does not always translate to a phenotypic ESBL profile [280]. Initially, *bla*_CTX-M_ often appears together with *bla*_TEM_, but it tends to become the dominant gene in a population over time [240].

#### 5.5.2. Fluoroquinolone Resistance in *P. mirabilis*

Fluoroquinolones are one of the most common classes of antibiotics that are used to treat infections in many countries throughout Western Europe, North America, and Japan. The spectrum of treatment with fluoroquinolones is extensive and includes infections such as UTIs [281]. Resistance in the bacterium *P. mirabilis* occurs via multiple mechanisms. The mechanism for high level resistance to fluoroquinolone antibiotics stems primarily from chromosomal alterations in the genes that encode the target enzyme, DNA gyrase (*gyr*A and *gyr*B) and topoisomerase IV (*par*C and *par*E) [282]. A recent study found that *gyr*A mutations were not significantly present in their sample groups [79], providing evidence that *gyr*A mutations may not be an important method for selecting for resistance in both broiler chickens and buffalo bacteria. Specific mutations within the conserved quinolone resistance-determining regions (QRDRs), such as S80 in *par*C and S83 in *gyr*A, are strongly linked to resistance, with a mutation at S464 in *gyr*B leading to an even higher resistance level [283]. Other contributing chromosomal mechanisms include alterations in the outer membrane and the action of efflux pumps, which reduce intracellular drug accumulation [284].

In addition to chromosomal mutations, plasmid-mediated mechanisms play a significant role. These include (i) protection of drug targets by *qnr* proteins (e.g., *qnr*A, *qnr*D, *qnr*S), (ii) drug modification by enzymes like *AAC(6′)-Ib-cr*, and (iii) active drug efflux by specific pumps such as *qep*A or *oqx*AB [285]. Data from Hasona et al. [79] revealed a variable prevalence of these plasmid-mediated genes in Egyptian isolates: the *qnr*A gene was detected in 47.06% of broiler chicken isolates but only 9.09% of buffalo isolates, while the *qnr*S gene was not found (0% in both sample types). These resistance genes are often located near β-lactamase genes (e.g., *bla*_TEM_, *bla*_CTX-M_) on mobile genetic elements like class 1 and class 2 integrons [286]. While the *qnr*A6 gene has been found to be chromosomally encoded in *P. mirabilis* [287], plasmid-borne *qnr* genes have achieved a global distribution. *Proteus* spp. Carrying plasmids with the *qnr*D gene have been reported in numerous countries [284,285,288]. The *qnr*C gene has been found on a plasmid also carrying resistance genes for ampicillin, sulfamethoxazole, and trimethoprim [289]. The *qnr*A and *qnr*S genes are reported less frequently [290,291]. From a One Health perspective, *qnr*A and *qnr*D genes have been detected in isolates from companion animals, while only *qnr*D has been identified in isolates from food products [58,59,82,185,204].

#### 5.5.3. Tetracycline Resistance in *P. mirabilis*

Tetracycline antibiotics are used to treat several GNB infections; however, high rates of resistance to these drugs have been documented within the *Enterobacteriaceae* family [292]. This resistance is primarily attributed to an efflux mechanism, where the antibiotic is actively pumped out of the bacterial cell. The genes responsible for this efflux are frequently located on mobile genetic elements; for instance, the class A tetracycline resistance (*tet*) determinant was the first to be identified from the RP1/Tn1721 transposon system [293]. In *Enterobacteriaceae*, tetracycline resistance is most commonly associated with the *tet*(A) to *tet*(E) gene determinants [294].

*P. mirabilis* exhibits a natural, intrinsic resistance to classical tetracycline, which is a key factor in its increasing tolerance to this drug class [295]. Tigecycline, a 9-t-butylglycylamido derivative of minocycline, is a newer tetracycline-class antibiotic developed to treat resistant GNB [296]. While the classical tetracycline resistance determinants do not affect tigecycline, the identification of the *acr*AB efflux pump in *P. mirabilis* provides an explanation for its reduced susceptibility to this last-resort drug.

A variety of tetracycline resistance genes, including *tet*(O), *cat*I, *tet*(J), *tet*(A48), *tet*(A), *tet*(B), *tet*(C), and *tet*(M), have been detected in isolates from farm animals, companion animals, and food products, though they have not been found in isolates from wild animals [34,59,78,82,204]. Supporting this, a recent study by Hasona et al. [79] reported a high prevalence of the *tet*(M) gene in *P. mirabilis* from Egyptian animals, found in 81.82% of broiler chicken isolates and 70.59% of buffalo isolates. The same study detected the macrolide resistance gene *erm*B in 23.53% of broiler isolates and 18.18% of buffalo isolates, indicating the co-circulation of resistance genes to multiple antibiotic classes within these populations. Supporting the global prevalence of tetracycline resistance mechanisms, a recent whole-genome sequencing study of 18 Indian *P. mirabilis* strains reported universal phenotypic resistance to tetracycline. Genomic analysis revealed that all but one isolate carried the intrinsic resistance gene *tet*(J), alongside other common determinants such as *tet*(D) and amphenicol resistance genes (*cat*, *cat*A4). These genes were frequently embedded within MGEs, underscoring the role of horizontal gene transfer in their maintenance and spread within this regional population [297].

#### 5.5.4. Aminoglycoside Resistance in *P. mirabilis*

Aminoglycosides are classified as broad-spectrum antibiotics; they are primarily produced from Actinomyces species and are indicated for use against both Gram-negative and Gram-positive pathogens [298]. While effective, the general use of aminoglycosides has been severely limited due to bacterial resistance and toxicity to humans and animals [299]. However, they remain one of the most available means of treating infections caused by MDR organisms [298]. The most frequently observed mechanism of aminoglycoside resistance involves enzymatic modification of the drug by AMEs. The second significant mechanism for aminoglycoside resistance is via 16S rRNA methylation, a process that gives rise to a high level of resistance to gentamicin, tobramycin and amikacin [300]. AMEs confer aminoglycoside resistance through the introduction of chemical modifications to the aminoglycoside molecule, and these enzymes are categorized by the type of modification they introduce acetyltransferases (AACs), phosphotransferases (APHs), nucleotidyltransferases, or adenyltransferases (ANTs) [301]. The genes encoding these enzymes are typically located on MGE, e.g., plasmids, integrons, transposons), and most often they co-occur with other genes that confer antimicrobial resistance; this facilitates their co-dissemination [298].

In *P. mirabilis*, the genetic determinants of aminoglycoside resistance most often occur in the form of gene cassettes located in integrons. Frequently identified genes include *aad*A1 and *aad*A2 (aminoglycoside adenyltransferases), *aad*B (aminoglycoside (2″) adenyltransferase), *aac(6′)-Ib* (also known as *aac*A4, an acetyltransferase), and *sat*2 (streptothricin acetyltransferase) [291]. A recent study by Hasona et al. [79] underscores the high prevalence of one such gene, reporting *aad*A1 in 97.06% of broiler chicken isolates and 100% of buffalo isolates. Furthermore, several variants of 16S rRNA methyltransferases (such as *rmt*A, B, C, D, and *arm*A) have been identified in *Enterobacterales*, including *P. mirabilis* [302]. These enzymes modify the aminoglycoside binding site on the ribosome, conferring high-level resistance to virtually all clinically available aminoglycosides [303]. Other potential resistance strategies include alteration of membrane proteins or the ribosome and increased drug efflux, but these are not as widespread as AME-mediated resistance [304].

From an epidemiological perspective, the distribution of specific resistance genes varies by source. Isolates from farm animals have been found to carry *aac(6′)-Ib-cr, aph(3′)-IIa, rmt*B, *aac*C1, and *aac*C2 [30]. In companion animals, detected genes include *aphAI-I*AB, *aac(3′)-*IV, *aac(6′)-Ib*, and *aad*A1 [58,59,204]. Meanwhile, foodborne isolates have been shown to harbor *aac(6′)-Ib-cr*, *aph(4)-Ia*, *aad*A1, *aad*A2, *aac(3′)-Ia*, *aac(3)-*IV, and *aac(3)-Iva* [79,82,185,305].

### 5.6. Co-Carriage of Multidrug Resistance Genes: The Convergence of Threats

Antibiotic resistance in *P. mirabilis* strains from animals and food is a major public health concern. The threat of this pathogen is exacerbated not only by the commonality of ESBL genes but by their frequent co-existence with a diverse array of other ARGs. These co-carried genes include those conferring resistance to tetracycline (*tet*(M)), sulfonamide (*sul*2), macrolide (*erm*B), quinolone (*qnr*A), chloramphenicol (*cat*A1), aminoglycoside (*aad*A1), trimethoprim (*dfr*A1), and colistin (*mcr*-1). The descriptions surrounding this co-carried profile have been documented in different reservoirs across many models [79].

Isolated strains from arm-Animal species; swine, avian, and duck species, contained numerous antimicrobial resistance genes, including *nor*A, *acr*B, *bla*_OXA_, *bla*_TEM_, *bla*_CTX-M_, *bla*_NDM_, *bla*_DHA_, and *bla*_KPC_ [32,52,57,78]. Isolated strains from pets; dog and cat, had *bla*_OXA-1_, *bla*_TEM_, *bla*_CTX-M_, and *bla*_DHA_ [58,59,204] while those from foods had *bla*_CTX_, *bla*_OXA_, *bla*_DHA_, *bla*_CMY-2_, *bla*_NDM_, *bla*_TEM_, *bla*_SHV_, *bla*_FOX_, *bla*_CIT_, *bla*_EBC_, and *bla*_MBL_ [52,82,185]. In addition, farm animal isolates also carry quinolone resistance genes, including *qnr*S, *par*C, *qnr*D, and *oqx*A [306].

This multidrug-resistant nature is exemplified by strains like XH983, which carries ARGs for aminoglycosides (*aph(3′)-Ia, aph(3″)-Ib, aph(6)-Id, aac(3)-IId, aad*A5, *aad*A1), β-lactams (*bla*_KPC-2_, *bla*_TEM-1B_), phenicols (*cat*, *cat*A1), sulfonamides/trimethoprim (*drf*A1, *drf*A17, *sul*1, *sul*2), and tetracycline (*tet(*J)) [307]. The emerging genes include *bla*_CTX-M-65_, identified in a Russian isolate with *bla*_VEB_, *aac6-Ib,* and *qnr*A1 [224] and show an increase in numbers of different resistance mechanisms for aminoglycosides (*aac*A4, *aad*B, *aph*A6), β-lactams with expanded spectrum (*bla*_VEB-6_), and carbapenem-resistant strains (*bla*_NDM-1_) [308].

ESBL production is strongly associated with fluoroquinolone resistance in clinical settings. This linkage is not merely epidemiological but is mechanistically explained by the frequent co-localization of their respective genetic determinants on the same mobile genetic elements. Specifically, plasmids harboring ESBL genes (especially *bla*_CTX-M_ variants) often also carry PMQR genes, such as *qnr* alleles (e.g., *qnr*A, *qnr*D, *qnr*S) and *aac(6′)-Ib-cr*, alongside determinants for sulfonamides, aminoglycosides, and tetracyclines [170,287]. Alabi This genetic linkage on conjugative plasmids (e.g., IncF, IncI types) facilitates the co-transfer and co-selection of multidrug resistance profiles during horizontal gene transfer events. Consequently, exposure to a single antibiotic class (e.g., β-lactams) can select for and maintain resistance to other unrelated classes (e.g., fluoroquinolones), severely limiting therapeutic options. These include aminoglycoside resistance genes (*aad*1, *aad*2, *aac3Ia*, *aac6-Ib*, *aph(3)-Ia*, *aph(6)-Id*), as well as genes for sulfonamide (*sul*1, *sul*2), trimethoprim (*dfr*A1, *drf*A32), chloramphenicol (*cat*, *cat*1), tetracycline (*tet*), and macrolide (*msr*) resistance, particularly alongside CTX-M-type ESBLs [170]. Studies have shown that a very high level of co-production of ESBLs with ampC and carbapenemase enzymes exists in India, as reported by Datta et al. [309]. The increasing trend of co-producing ampC with ESBLs raises the concern of increasing the area of β-lactam resistant. A study in India identified that co-production of an ampC enzyme among 19.4% of the ESBL-producer sample was noted [220].

Current monitoring studies demonstrate the vast prevalence of the co-production of these genes, especially concerning key livestock reservoirs in Egypt. Among sets of broiler chicken isolates, there were high levels of prevalence with *int*1 (97.06%), *dfr*A1 (100%), *sul*2 (97.06%), *aad*A1 (97.06%), *tet*(M) (81.82%), *qnr*A (47.06%), and *mcr*-1 (11.76%). A similar presence was documented for the β-lactamase gene, with *bla*_TEM_ being nearly ubiquitous (97.06%) while *bla*_CTX-M_, *bla*_OXA-10_, and *bla*_CMY-2_ had respective detection frequencies of 26.47%, 2.94%, and 41.18%. The buffalo isolates also demonstrated similar patterns of prevalence for *int*1, *dfr*A1, and *aad*A1 (all 100%), *sul*2 (90.91%), *bla*_TEM_ (81.82%), and *bla*_CMY-2_ (63.64%). In summary, 97.06% of the broiler isolates and 81.82% of the buffalo isolates contained β-lactamase genes [79]. The ability to produce this broad, extensive, and genetically linked resistance profile supports the assertion of *P. mirabilis*’s complex, co-selected nature as a multidrug resistant organism. The conjunction of β-lactam resistance with determinants for the other antimicrobial classifications creates an extremely limited number of therapeutic options for treating patients with these infections, making it imperative that all sectors of animal, food, and human health begin to develop an integrated surveillance and antimicrobial stewardship infrastructure to combat this situation. Most importantly, we must adopt a wider evolutionary and ecological viewpoint of the issue [310]. When reviewing the molecular epidemiology of AMR in *P. mirabilis*, it is clear that this pathogen is exceptionally well-adapted. It can continually acquire and disseminate many resistance genes through its extensive mobile genome (mobilome). The presence of strong genetic evidence indicating transmission from animal to human through food certainly depicts a severe One Health crisis (Table 3). The overall impressive transition of this pathogen from a traditional opportunistic pathogen to a means of spreading pan-resistant infections beyond recognized therapeutic limits will represent a healthcare crisis for both humankind and the animal kingdom. The widespread distribution of identical resistance genes across human, animal, and food reservoirs, as detailed above, makes it clear that isolated interventions in any single sector are insufficient. The following section outlines integrated, cross-sectorial strategies essential for containing the spread of resistant *P. mirabilis*.

## 6. The One Health Approach to Combat *P. mirabilis* AMR: Collaborative Strategies

**Figure 4:**
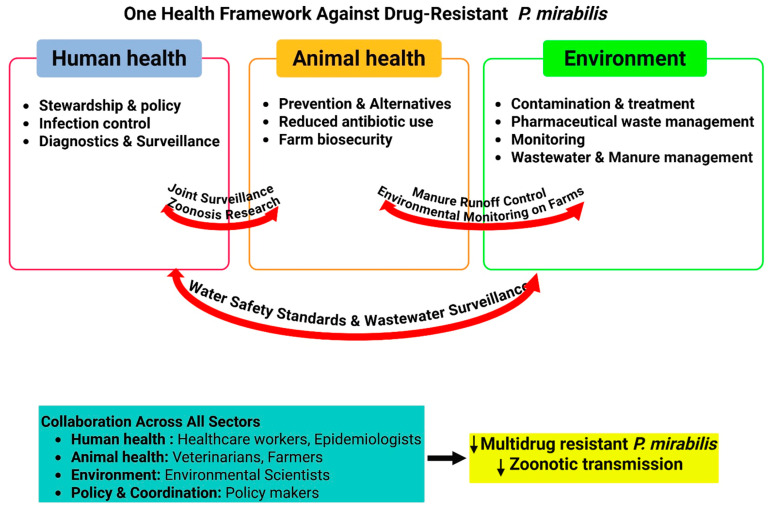
A proposed One Health intervention framework to combat MDR *P. mirabilis*. The schematic outlines an integrated, cross-sectoral strategy required to mitigate the emergence and spread of drug-resistant *P. mirabilis*. The framework is structured around three core pillars—Human Health, Animal Health, and Environmental Health—each with targeted interventions. The bidirectional arrows represent critical inter-sectorial linkages: between human and animal health, they indicate joint zoonotic surveillance, antimicrobial resistance monitoring, and translational research at the human–animal interface; between animal and environmental health, they reflect coordinated actions to control manure runoff, implement farm-level environmental monitoring, and manage pharmaceutical waste from livestock; and between environmental and human health, they denote a continuous feedback loop through wastewater surveillance, water safety standards, and tracking of environmental contamination routes to human populations. These arrows signify continuous feedback, data sharing, and synchronized interventions across all domains, reinforcing that no sector operates in isolation and that effective containment of MDR pathogens requires simultaneous, cross-cutting actions involving all stakeholders. Collaboration across all sectors is operationalized through the engagement of healthcare workers, epidemiologists, veterinarians, farmers, environmental scientists, and policymakers. Collectively, this coordinated One Health approach aims to disrupt zoonotic transmission cycles and reduce the burden of drug-resistant *P. mirabilis *in human, animal, and environmental reservoirs.

According to WHO [311] and Franklin et al. [312], the One Health approach is a cooperative, multidisciplinary method that aims to achieve optimal health outcomes for people, animals, and ecosystems while acknowledging their interconnection (Figure 4). Given that the same antimicrobial medications are used in human medicine, veterinary care, and agriculture and that both humans and animals can carry and spread the same resistant pathogens, this framework has been widely adopted to address the complex problem of AMR [311]. A One Health approach to AMR recognizes the need for an all-encompassing system that incorporates plant, animal, and human health and takes into account the environment’s crucial role in promoting the spread of bacteria and resistance genes [311]. Understanding the fundamentals of evolutionary biology and how they relate to prokaryotes is essential for long-term management of infectious diseases [313,314]. An understanding of this complex issue is reflected in the global strategy for AMR surveillance, which is in line with the One Health concept [315,316]. The shift to integrated One Health surveillance systems is presently taking place in a number of different parts of the world.

Addressing AMR effectively requires insights from multiple disciplines, framing it squarely within the One Health approach [317,318,319]. Central to this approach is antibiotic stewardship, which is recognized as vital for directly reducing the selection pressure that drives resistance [320,321]. The implementation of One Health principles is therefore pivotal in combating antibiotic resistance effectively [322]. This approach underscores the interdependence of human, animal, and environmental health and emphasizes the importance of collaboration across scientific, social, and political sectors [323] to effectively monitor the emergence and movement of resistance genes and resistant bacteria within and between these different compartments. A major driver of AMR within this context is the overuse and misuse of antimicrobials in animal agriculture, a practice intimately connected to bacterial zoonosis that has significantly accelerated the emergence of resistance [95].

One Health is characterized by an integrated, unifying effort that operates at local, national, and global levels to achieve the best possible health for people, animals, and our environment [324]. Placing AMR on the international political agenda and tackling it through a One Health lens is crucial for driving meaningful change. In a key step, the WHO adopted the Global Action Plan on AMR in 2015, urging countries to establish national surveillance systems for bacteria from both humans and animals and explicitly advocating for a One Health strategy [325]. AMR is a global health issue that perfectly exemplifies the need for a One Health approach. Because bacteria and their mobile genetic elements readily cross species and environmental boundaries, it is critical to understand the connections between the microbiota of humans, animals, and ecosystems—the core of the One Health concept [326,327]. This interconnectedness demands a unified approach that acknowledges the inextricable links between human, animal, and environmental health [322].

National and international bodies have embraced this framework. The U.S. National Action Plan for Combating Antibiotic-Resistant Bacteria (2020–2025) explicitly includes the One Health approach as an effective strategy [328]. A primary objective is to promote antibiotic stewardship in animals, eliminate the use of medically important antibiotics for growth promotion, and bring all other uses of these drugs under veterinary oversight [323]. Globally, the quadripartite organizations—the Food and Agriculture Organization (FAO), the World Organization for Animal Health (OIE), the WHO, and the United Nations Environment Program (UNEP)—have established a joint One Health action plan and a strategic framework for collaborative work on AMR [329]. Assessing the true extent of AMR in LMICs remains challenging due to inadequate surveillance, though initiatives like the WHO’s Global Antimicrobial Resistance and Use Surveillance System (GLASS), launched in 2015, are important steps forward, to fill knowledge gaps and guide strategies at all levels. GLASS was created to progressively integrate surveillance data on antimicrobials used in humans, track antimicrobial use, and understand the role of AMR in the food chain and the environment [330].

Other major strategies highlighting the importance of a multisectoral One Health response include the United States National Action Plan for combating antibiotic-resistant bacteria [331] and the WHO Global Action Plan [325], the 2016 United Nations General Assembly Declaration on AMR [332], and the FAO/OIE/WHO Tripartite Collaboration [333,334]. The threat of AMR is profound, with the potential to hinder infectious disease control, increase healthcare costs, and reverse decades of progress in medicine [335]. Through its comprehensive and collaborative framework, the One Health approach provides a powerful and necessary means to limit the spread of AMR and safeguard modern medicine from the threat of a post-antibiotic era.

### 6.1. Integrated Surveillance and Monitoring Programs for Antimicrobial Resistance in P. mirabilis

Implementing continuous surveillance programs to monitor antimicrobial resistance patterns in *P. mirabilis* is crucial for developing effective treatment guidelines and shaping public health policies [33,336]. These surveillance systems collect and analyze data on resistance trends and antimicrobial consumption from the human health, animal health, and environmental sectors, thereby offering a holistic One Health perspective on the AMR situation.

Conducting extensive monitoring of resistance trends in diverse geographical areas and various healthcare environments yields critical intelligence on the dynamically changing resistance profile of this pathogen [18,297]. The use of standardized protocols for data collection, coupled with advanced molecular techniques like whole-genome sequencing, is instrumental for identifying the origins of resistance genes and tracking the dissemination of resistant clones. The critical need for integrated, genomics-driven surveillance is powerfully illustrated by recent studies. Global genomic analyses have mapped the phylogeographic spread of resistant clones and quantified the carriage of ARGs and MGEs across continents [95]. Concurrently, national-level sequencing efforts, such as those analyzing strains from India, provide granular insights into local resistance patterns and virulence gene repertoires, demonstrating the utility of WGS in outbreak investigation and resistance tracking [297]. These studies collectively advocate for the establishment of *P. mirabilis* as a key sentinel organism for AMR surveillance within the One Health paradigm. Fostering collaboration and sharing data across different sectors enriches the overall understanding of AMR epidemiology and facilitates a swift, coordinated response to emerging threats. Simultaneously, monitoring how antimicrobials are used helps identify areas of misuse and provides a solid foundation for stewardship programs.

The evidence generated by this integrated surveillance is pivotal; it enables healthcare institutions and public health authorities to design and implement focused antimicrobial stewardship initiatives and robust infection control practices to curb the spread of resistant *P. mirabilis* strains [181,337]. In summary, a unified, cross-sectoral surveillance system produces the essential evidence needed to direct precise interventions and bolster the global effort against AMR.

### 6.2. Antimicrobial Stewardship: Promoting Responsible Use in Human and Veterinary Medicine

Antimicrobial stewardship is a systematic approach designed to encourage appropriate use of antimicrobials, which is one of the key components in addressing antimicrobial resistance for *P. mirabilis*. This holistic method includes unique but interrelated actions in both the veterinary and human healthcare settings. In the area of human healthcare, comprehensive stewardship programs emphasize appropriate prescribing based on scientific evidence, use of accurate and prompt diagnostic tools to promote targeted therapy rather than empirical therapy, and educate patients on appropriate use of antibiotics [330]. In the veterinary agrarian sectors, antimicrobial stewardship mandates that all forms of antibiotic applications be under the direction of qualified health professionals. In this case, there is a ban against using antibiotics for the enhancement of growth, instead promoting alternative prophylactic measures such as vaccinations and better husbandry practices; and strictly adhering to through prudent-use guidelines for using antibiotics, including bans on antibiotics that are classified as critically important for treating humans [3].

It is essential that stewardship principles are consistently and coordinately applied in all sectors of the world. Reducing the total volume and inappropriate use of antimicrobials decreases the selective pressure for the development of resistance. Decreasing the overall volume of antimicrobials and limiting inappropriate use reduces the risk for development and transmission of resistant *P. mirabilis* strains and is critical for ensuring the continued effectiveness of current antimicrobials in the treatment of infections caused by *P. mirabilis* for decades to come. The partnership plans to develop and implement five new treatments for drug resistant bacteria, identified by WHO as the highest priority for action by 2025 [325].

### 6.3. Integrated Surveillance and Cross-Sectoral Infection Control Strategies

Employing a robust surveillance system wherein data on *P. mirabilis* resistance patterns is collected, analyzed systematically and disseminated to provide clinicians with essential information that will aid in administering optimal/effective treatment approaches [263,338]. Additionally, implementing strict biosecurity/infection control measures to contain the spread of antimicrobial resistant *P. mirabilis* between humans and animals and the environment. In health care settings, this includes adhering to sound hand hygiene practices, ensuring thorough cleaning/disinfection of the environment, isolating infected/colonized patients when indicated, and conducting proper management of urinary catheters to prevent the spread of healthcare acquired infections. In agriculture, it involves ensuring good sanitary conditions on farms, controlling movement of livestock, implementing proper manure/waste management systems and measures to control the spread of resistant bacteria. In addition to the above, it is important to consider safe food handling, improving wastewater treatment to eliminate biological contaminants, and monitoring the quality of water regularly at the community/environmental levels. In order to achieve the goal of interrupting/transmitting resistant *P. mirabilis* through all sectors by implementing a multi-faceted approach to prevention/control, it would help greatly in the global fight against antimicrobial resistance.

### 6.4. Innovative Therapeutic Strategies: Development of New Antimicrobials and Alternative Therapies

Antimicrobial stewardship and infection control are essential components in the fight against *P. mirabilis* AMR, but they will only be successful if new and innovative antimicrobial agents and treatment options that can help manage infections caused by this pathogen are developed [184,339]. The increased prevalence of AMR is largely due to the presence of intrinsic factors, as well as the acquisition of resistance genes from other organisms via horizontal gene transfer. As such, it is critical that an extensive and diverse assortment of drugs is available to effectively deal with the growing incidence of AMR infections [340,341].

Given the history of limited antibiotic development, the emergence of new resistance mechanisms is a major concern. Therefore, there is an urgent need for the identification and clinical testing of new antibiotics containing unique mechanisms of action that can be used to treat multidrug-resistant *P. mirabilis* [182]. The study of potential antibiotic adjuvants, including β-lactamase inhibitors and efflux pump inhibitors, to assist in restoring the efficacy of current antibiotics against resistant strains of *P. mirabilis* is an exciting area of research [183,342]. Beyond this line of research, continued exploration of new and alternative sources, as well as new classes of antimicrobial compounds, will provide additional support in overcoming existing resistance. Such innovative approaches not only focus on the destruction of bacteria cells but also on rendering them ineffective, less selective for resistance development, and exploring unconventional mechanisms. Alternative methods to the conventional usage of antibiotics have been explored for their potential in the improvement of treatment outcomes by developing innovative treatment modalities. The major areas of focus for this purpose are as follows:

### 6.5. Alternative Mechanisms to Treat Resistant Bacteria

The development of alternative mechanisms represents a critical frontier in overcoming antimicrobial resistance. Various alternative therapies, including vaccines, antibodies, pattern recognition receptors, probiotics, bacteriophages, antimicrobial peptides (amps), phytochemicals, metals, and antimicrobial enzymes, all offer approaches to combat microbial Infections [343]. Key strategies include:

#### 6.5.1. Fecal Microbiota Transplantation (FMT)

Antibiotic-mediated disruption of the colonic flora compromises colonization resistance, a key host defense. FMT corrects this dysbiosis by reintroducing a healthy donor microbiota, thereby restoring ecological balance and intestinal function [344].

#### 6.5.2. Fusion Protein Technology

This technology addresses significant challenges in the recombinant expression and stability of Bacteriologically Active Antimicrobial Peptides (BAMPs). The approach is successfully utilized in transgenic expression systems to produce functional peptides. According to Statistical data from the Antimicrobial Peptide Database 3 (APD3, https://aps.unmc.edu/) (accessed on 10 September 2025), there are 3940 known AMPs from the six kingdoms of life. This includes 383 bacteriocins from bacteria; 5 from archaea; 8 from protists, 29 from fungi; 250 from plants; and 2463 from mammals, including some Synthetic peptides [345]. AMPs from a range of species, including amphibians, insects, mammals, and fish, account for 75.65% Of the total number of AMPs. 13.5% of AMPs originate from plants, and 8.53% come from bacteria [346]. AMPs offer a promising avenue for combating infectious diseases and ameliorating the impacts of AMR [347]. A significant number of AMPs have demonstrated antibacterial properties against specific pathogenic microbes [348], through either membrane or non-membrane mediated mechanisms, these peptides, encompassing both Gram-positive and Gram-negative types, have demonstrated robust antibacterial activity against a broad range of both Gram-positive and Gram-negative bacteria [345].

Importantly, the antibacterial property of AMPs is thermostable, and the producer strain Offers a certain degree of self-protection against its own antibacterial peptide [349]. The mechanism of AMPs is to attack bacterial membranes by forming pores in the outer membrane [345]. Additionally, some AMPs kill pathogens by inhibiting intracellular pathways such as cell division [350], DNA migration [351], RNA polymerase [352], cell envelope [353], or cell wall biosynthesis [354,355]. Additionally, certain AMPs are effective against some membrane-enveloped viruses that can disrupt specific membranes in the host. This dual function is thought to arise due to the interdependent relationship between viruses and hosts, as viruses rely on the host replication machinery. Consequently, AMPs may offer the beneficial side effect of protecting against certain viral infections [356]. Additionally, certain AMPs are effective against some membrane-enveloped viruses that can disrupt specific membranes in the host. This dual function is thought to arise due to the interdependent relationship between viruses and hosts, as viruses rely on the host replication machinery. Consequently, AMPs may offer the beneficial side effect of protecting against certain viral infections [356]. Due to their broad-spectrum activity and low likelihood of inducing resistance, ABPs have emerged as promising alternatives to conventional Antibiotics in the fight against drug-resistant pathogens.

#### 6.5.3. CRISPR-Cas Systems

CRISPR-Cas systems, which originated as a bacterial adaptive immune mechanism, have emerged as a powerful and precise tool in addressing the global challenge of AMR. The versatility of this system is further exemplified by the expanding arsenal of Cas proteins [357] including Cas9, Cas12, Cas13, and Cas14 each offering distinct targeting capabilities (e.g., DNA or RNA) sequences, enabling versatile strategies to combat resistant pathogens [358]. These programmable systems are being developed as next-generation antimicrobials due to their ability to target bacterial DNA or antibiotic resistance genes with sequence-specific accuracy. This strategy represents a novel approach that bypasses traditional resistance mechanisms and enhances therapeutic specificity [359,360]. Furthermore, they offer a transformative pathway to combat AMR, with potential applications extending beyond conventional strategies. Beyond this application, the CRISPR-Cas9 platform has facilitated substantial advancements across medicine, enabling innovative therapeutic strategies for a range of intricate ailments including genetic disorders and malignancies, while also serving as a powerful diagnostic and therapeutic tool in microbiology [357]. Integrating these technologies with existing methods could significantly improve the management and potential reversal of the growing AMR crisis. Ongoing research continues to evaluate their effectiveness, address delivery and specificity challenges, and facilitate clinical translation [361,362]. Future progress is contingent upon overcoming technical and ethical barriers to enable safe and effective application. Key specific concerns that necessitate meticulous deliberation include off-target effects, poor delivery efficiency to target tissues, and potential unwanted side effects [357]. Therefore, underscoring the necessity for interdisciplinary collaboration and international cooperation to fully harness their potential against drug-resistant pathogens, more rigorous preclinical and clinical experiments are essential before broad human application [357].

Building on precision genetic tools, micro-/nanomotors (MNMs) are autonomously moving particles that actively penetrate biofilms. This capability enables localized antimicrobial delivery and physical biofilm disruption. A promising forward-looking strategy involves merging this active delivery with CRISPR precision by loading CRISPR payloads onto functionalized MNMs. Such a hybrid system could navigate biofilms to deliver genetic cargo directly to bacterial cells, potentially overcoming the critical delivery barriers that limit standalone CRISPR therapies [363].

#### 6.5.4. Antimicrobial Peptides (AMPs)

Antimicrobial peptides (AMPs), naturally occurring molecules derived from animal and microbial sources, represent a promising new class of antibiotics that primarily exert their effect by targeting the bacterial cell membrane [298]. These peptide-based antimicrobials have garnered significant interest due to their unique amphipathic structure, which is conferred by a combination of positively charged amino acids (such as lysine, arginine, and histidine) and a high proportion (often 50%) of hydrophobic residues [301,302]. Typically comprising fewer than 100 amino acids, this structure enables potent interaction with and disruption of microbial membranes.

A key advantage of many AMPs is their capacity for multi-mechanistic action, which enhances their overall antimicrobial efficacy and can reduce the potential for resistance development. For instance, the human cathelicidin LL-37 demonstrates not only direct bactericidal activity but also immune-modulatory and anti-biofilm properties [364]. Aires Host-derived AMPs such as lipocalin, hepcidin, and LL-37—isolated from sources like human bone marrow and umbilical cord mesenchymal stem cells (MSCs)—exhibit potent, broad-spectrum antibacterial properties [365].

However, some therapeutic challenges remain. Certain peptides have been associated with notable nephrotoxicity, primarily linked to the high doses required for clinical efficacy [210]. Furthermore, other significant limitations hinder their clinical translation, including susceptibility to proteolytic degradation, potential host cytotoxicity, and high production costs. Several peptide-based antibacterials are successfully used in medicine. Commercially available examples include dalbavancin, daptomycin, colistin (polymyxin E), oritavancin, polymyxin B, teicoplanin, vancomycin, and telavancin [366].

#### 6.5.5. Efflux Pump Inhibitor (EPIs)

Another strategic approach to combat antimicrobial resistance is the utilization of Efflux Pump Inhibitors (EPIs). These compounds function by obstructing the activity of bacterial efflux pumps, which are key mediators of multidrug resistance. When integrated into combination therapies with existing antimicrobials, EPIs can significantly restore and enhance drug efficacy [367].

EPIs are generally characterized as simple, stable, and affordable molecules with a favorable safety profile for human use [368]. A notable secondary benefit of certain EPIs is their additional capability to inhibit bacterial biofilm formation, thereby targeting another major resistance and virulence mechanism. Clinically relevant examples of EPI compounds include thioridazine, phenylalanine-arginine β-naphthylamide (PaβN), and the arylpiperazine derivative 1-(1-naphthylmethyl)-piperazine (NMP).

#### 6.5.6. Bacteriophage (Phage) Therapy

Bacteriophage (phage) therapy involves the use of viruses that specifically infect and lyse bacterial hosts, representing a promising alternative for combating MDR pathogens. Often described as “bacteria eaters,” phages offer several distinct advantages over conventional antibiotics, including high host specificity, natural self-replication at the infection site, low inherent toxicity, minimal environmental impact, and the absence of cross-resistance with antibiotics. Their diversity and relative ease of isolation contribute to their therapeutic potential.

Clinically, phage therapy has demonstrated notable efficacy in treating localized and topical infections where conventional antibiotics have failed. Promisingly, combination strategies employing both phages and antibiotics have shown synergistic effects, particularly in eradicating challenging multidrug-resistant biofilms [369].

Engineered lysins and bacteriophage therapy: Modified lysins with enhanced therapeutic profiles and carefully selected bacteriophages offer highly targeted and potent alternatives to conventional antibiotics. These biological agents show significant promise against multidrug-resistant bacteria, including specific resistant strains [370,371]. The high specificity of phages can restrict their broad applicability. Additionally, bacteria may evolve resistance to phage infection. The clinical translation also faces complex regulatory and manufacturing hurdles.

#### 6.5.7. Immunotherapy

Immunotherapy encompasses therapeutic strategies designed to enhance, modulate, or harness the body’s immune system to combat infections more effectively (e.g., immunotherapy, procurement of lymphoid tissue stem cells). By leveraging the inherent specificity and adaptability of the immune response, these strategies offer a promising avenue for addressing antibiotic-resistant infections, potentially overcoming key limitations of traditional antibiotics. These include the risk of triggering autoimmune reactions, variability in patient responses, and the typically lengthy and complex development and regulatory pathways.

#### 6.5.8. Antivirulence Therapy

Antivirulence therapy represents a novel therapeutic approach that focuses on suppressing or eradicating the virulence factors secreted by a pathogen, rather than directly killing the bacterium itself. The primary intention is to disarm the pathogen and mitigate infection severity while reducing the selective pressure that drives the development of traditional antimicrobial resistance. However, this strategy presents certain challenges. It may not achieve complete pathogen eradication, necessitating a precise and thorough understanding of intricate virulence mechanisms. Additionally, there remains the potential for pathogens to evolve compensatory virulence pathways. Another innovative method combines antibacterial sonodynamic therapy with antivirulence immunotherapy by using engineered nanovesicles to capture bacterial toxins and generate reactive oxygen species upon ultrasound activation [372].

#### 6.5.9. Probiotic and Prebiotic Supplementation

This strategy involves modulating the host microbiome to enhance its ability to manage and prevent pathogen colonization, thereby competitively excluding potential invaders. This strategy shows particular promise in managing infections caused by Proteus mirabilis. Specific strains of *Lactobacillus*, particularly *Lactobacillus gasseri* (*L. gasseri*), have demonstrated the ability to help prevent *P. mirabilis*-induced urinary stones. The primary mechanism involves the production of lactic acid, which directly inhibits the growth of *P. mirabilis* and critically blocks its urease enzyme activity. Lactic acid competes with urea for binding to urease, thereby reducing the production of ammonia and subsequent crystallization. This results in diminished biofilm formation and fewer urinary stones [373]. The efficacy of this approach can be constrained by its strain-specific nature, variable colonization success in the host, and potentially limited impact against severe, established infections.

#### 6.5.10. Drug Repositioning

Drug repositioning is a strategy that involves identifying new antimicrobial applications for existing drugs already approved for other clinical indications. This approach, which includes combining non-antibiotic drugs with traditional antibiotics to enhance their effectiveness, offers a pathway to accelerate the development of new anti-infective therapies. A key advantage is the potential to achieve therapeutic effects with lower doses of the antibacterial agent, thereby minimizing adverse impacts on healthy cells and tissues [374]. In addition, reactive nitric oxide (NO) and reactive oxygen species (ROS) produced by metallic NPs harm bacterial cell components [375]. Repositioned agents often exhibit more modest antimicrobial potency compared to purpose-developed antibiotics, necessitating extensive clinical validation for their new indication. Furthermore, the potential for unexpected off-target side effects remains a significant consideration during development.

#### 6.5.11. Machine Learning and Artificial Intelligence for Predicting Antimicrobial Resistance

The application of machine learning (ML) and artificial intelligence (AI) offers a transformative, data-driven alternative for combating antimicrobial resistance by enabling early prediction and informed decision-making, thereby optimizing therapy and stewardship. A pivotal challenge in managing bloodstream infections is the 24–72 h delay in obtaining culture and susceptibility results [376], a period during which clinicians often rely on empirical broad-spectrum antibiotics that can inadvertently exacerbate AMR if inappropriate [377]. Developing tools to guide more targeted treatment during this window is essential for improving outcomes and curbing resistance [378]. Toward this goal, researchers have developed supervised ML models to predict the presence of resistant organisms in blood cultures from data available at the patient’s first encounter. Utilizing algorithms such as penalized logistic regression, random forest, and XGBoost to classify key resistant pathogens (including ESBL, CRE, ampC, MRSA, and VRE), studies have demonstrated high predictive performance [379]. This approach can reduce unnecessary broad-spectrum use and associated costs by providing early, evidence-based guidance.

The promise of ML extends to other infections, such as UTIs, where predictive tools using electronic health record data have shown potential for forecasting bacterial growth and resistance patterns before culture results are available [380]. Furthermore, recent advancements include integrating socio-economic deprivation indices into AMR modeling, combining demographics, infection history, and comorbidities to offer real-time decision support for infections like sepsis [380]. Beyond clinical predictors, AI and ML are revolutionizing the analysis of genomic data for AMR. AI-driven tools can automate the identification of resistance genes from sequencing data, predict phenotypic resistance patterns, and help optimize personalized treatment strategies, significantly accelerating the interpretation of complex genomic datasets [381]. This represents a powerful future direction for resistance surveillance and precision infectious disease medicine.

#### 6.5.12. Plant-Derived Essential Oils (Eos)

Despite the promising therapeutic potential of plant-derived essential oils (Eos) as anti-biofilm agents, their practical application faces substantial challenges. The bioactive components of Eos may exhibit dose-dependent toxicity, pose organ-specific risks, and present administration-route difficulties. Current literature underscores critical gaps, including a lack of standardized toxicological assessments, divergent safety profiles among different Eos, and insufficient in vivo validation of efficacy and safety. Collectively, these hurdles significantly impede the translation of Eos from laboratory research to broad clinical or industrial applications [382,383].

#### 6.5.13. The Role of Nanotechnology and Biopolymers: A Case Study in *P. mirabilis*

Nanotechnology represents a significant innovative frontier in combating resistant pathogens. Nano-formulations offer advantages such as improved drug delivery, enhanced bioavailability, and intrinsic antimicrobial activity. A key biopolymer in this field is chitosan (CS), a naturally occurring, biodegradable, and biocompatible positively charged polysaccharide derived from chitin. Its antimicrobial activity stems from electrostatic attraction to negatively charged bacterial cell surfaces, leading to membrane damage and disruption of cellular processes [384].

The true potential of chitosan is unlocked through nanoformulation. Chitosan nanoparticles (CSNPs) exhibit enhanced efficacy due to their high surface area-to-volume ratio, increased cellular uptake, and ability to overcome bacterial resistance mechanisms [385]. This makes them promising candidates against XDR and PDR bacteria, including *P. mirabilis*. Applied research demonstrates this potential against *P. mirabilis*. For instance, a trimethoprim nanoemulsion showed an eight-fold reduction in MIC compared to the plain drug [386]. Synergistic effects have also been observed with combinations like curcumin-silver nanoparticles (Cur-AgNPs) [387].

The arsenal of effective nanomaterials is expanding. Biosynthesized ZnO-CuO nanocomposites have shown significant promise against MDR *P. mirabilis* [388], these nanoparticles inhibited biofilm formation in robust biofilm-producing strains over 24–48 h. Furthermore, treatment with the ZnO-CuO nanocomposite led to the downregulation of the *lux*S gene, a key regulator of quorum sensing [388].

A recent translational study evaluated CS, CSNPs, and CSNPs combined with ciprofloxacin (CIP) against XDR/PDR *P. mirabilis* isolates from livestock [79]. The key findings were (Table 4):

1. Synergistic enhancement: The CSNPs + CIP combination showed a 50–58% synergistic increase in the inhibition zone area compared to CIP alone.
2. Activity against extreme resistance: CSNPs were highly active (MICs: 0.067–0.081 mg/mL) against all isolates, irrespective of their XDR/PDR status or the presence of resistance genes (*bla*_TEM_, *mcr-*1, *qnr*A). This suggests CSNPs’ membrane-disruptive, multi-targeted action bypasses common resistance mechanisms.
3. Comparative advantage: The efficacy of CSNPs was competitive or superior to other nanomaterials like AgNPs, CuNPs, ZnNPs, and Se-based composites tested against *P. mirabilis* [386,387,388,389,390,391].
4. Anti-virulence activity: Sub-MICs of both CS and CSNPs significantly reduced the swarming motility of *P. mirabilis*, a key virulence factor for colonization [79]. This aligns with antivirulence strategies, where CS-based materials can attenuate bacterial pathogenicity, potentially impeding biofilm formation and increasing susceptibility to host defenses and antibiotics.

The objective of these nano-strategies is twofold: to re-sensitize MDR isolates to conventional antibiotics and to provide alternative therapeutic approaches [248]. The use of antimicrobial nanomaterials has garnered considerable attention in managing infections caused by MDR pathogens like *Proteus* [79,392]. Accelerating the development and regulatory approval of such novel tools is crucial to ensure effective treatment options for highly resistant strains. While antimicrobial stewardship to prevent resistance emergence is paramount, the parallel development of novel agents and alternative strategies—exemplified by CSNPs, phage therapy, and antivirulence compounds—is a vital long-term solution to this public health crisis [79,392].

#### 6.5.14. Public Awareness and Education as Cornerstones of AMR Mitigation

Public engagement and educational efforts are basic building blocks of the struggle against AMR in a One Health approach worldwide. Such efforts are the backbone of the broader approach used to address issues of antibiotic resistance [393]. It is important to conduct targeted educational efforts aimed at various segments of society such as the general population as well as healthcare professionals and agricultural workers regarding the basics of rational use and the grave risks associated with irrational use of antibiotics.

The major learning objectives include understanding what AMR is and what its health-related meaning and significance are, promoting prudent usage in both human and veterinary health, and stressing the significance of hygiene- and food-related practices, as well as effective vaccination practices, and making one aware of how everything is interlinked in human, animal, and environmental health. The above-mentioned integrated approach to education helps bring about prudent behavior and, in this way, decreases irresponsible usage and, in this manner, establishes an anti-microbial culture in society in this context [394]. Such initiatives are further supplemented by government policies, some of which proclaim veterinary monitoring and prescription necessary in animal farming for using antibiotics [394]. Moreover, targeted policies are in place that do not allow the use of certain classes of antibiotics defined as critically important for human use, including fluoroquinolones and third-generation cephalosporins, in agricultural facilities with the aim of conserving them for human use [395,396]. Raising public consciousness and encouraging positive changes in behavior, public awareness campaigns could make a significant contribution to limiting the spread and effects of AMR, including the development of *P. mirabilis* resistance.

### 6.6. Challenges and Opportunities in Combating P. mirabilis Antimicrobial Resistance

Despite extensive worldwide efforts, there are several factors that impede the progress being made against AMR in *P. mirabilis*. First among these is the increased antibiotic resistance *P. mirabilis* exhibits upon biofilm formation, which drastically reduces the efficacy of typical antibiotic treatment schemes [141]. Second, the promising alternative bacteriophage therapy also faces severe obstacles such as the development of bacterial resistance to phages themselves and intricate regulatory pathways, which further delay its clinical use [243]. The pathogen exhibits an astonishing ability to acquire and disseminate novel resistance mechanisms rapidly, especially by means of MGEs, thereby allowing new resistance patterns to emerge and spread more rapidly than the pace of developing new therapeutic drugs. This remains a significant bottleneck in the intrinsically slow and expensive process of antibiotic discovery and development, leaving an increasingly narrow arsenal to fight MDR infections.

Adding to such treatment challenges are the systemic and surveillance gaps. There are also major gaps in integrated, One Health models of surveillance that cover the human, animal, and environmental contexts. This is because of the lack of a comprehensive approach to AMR epidemiology and the tracking of resistant strains. Moreover, a cultural change, which is a long-term and hard-won approach to counter the deeply ingrained practice of using antibiotics ineffectively in human and veterinary settings, requires an even greater approach to improve and enhance personal and environmental cleanliness and standards of infection control. The environmental release of resistant bacteria and ARGs continues to drive the cycle of AMR, which is a never-ending cycle. It is critical to tackle these complex problems in order to improve patient outcomes. The future of research needs to focus on developing innovative combination regimens involving the combination of traditional antibiotics with new strategies, including nanoparticles and phage therapy. Additionally, there is an urgent need for clinical trials aimed at validating the safety and efficacy of such emerging therapies.

However, there are significant opportunities to move forward in these challenges:

1. Help the One Health approach: Interdisciplinary collaboration and a flow of data maybe the starting point for a more coordinated approach to AMR.
2. Leveraging technological innovations: Advances in the areas of genomics, bioinformatics, and rapid diagnostics offer immense possibilities in identifying resistance genes quickly, tracing the path of their transmission, and elucidating their evolution.
3. Funding novel therapeutic approaches: Ongoing efforts in the development of novel therapeutic modalities, such as phage therapies, antivirulence agents, and immunotherapeutic drugs, could potentially offer much in the way of novel
4. Enactment of strong policy frameworks: The formulation and launch of effective international and national policies on antimicrobial stewardship, comprehensive surveillance, and the environment can help bring about major shifts. The areas of intervention are the development of “super” methods of surveillance and diagnosis that understand the zoonotic disease transmission to help shape the policy, development of infrastructure and education resources and availability of drugs in LMICs, development of guidelines based on “best practices” of existing treatments available, and investment directed at the development of “novel” therapies.

## 7. Concluding Remarks, One Health Implications, and Future Directions

*P. mirabilis* exemplifies the complex nexus of sophisticated virulence, rapid AMR acquisition, and cross-sectorial transmission that defines contemporary global health threats. This review underscores its evolution from a common uropathogen to a formidable multidrug-resistant adversary, whose clinical impact is amplified by its seamless circulation across human, animal, and environmental reservoirs. The evidence compels a unified perspective, crystallized in four key interconnected messages:

1. Shared reservoirs and transmission pathways: *P. mirabilis* thrives in interconnected ecosystems. Genetically similar, multidrug-resistant strains circulate among humans, food-producing and companion animals, and environmental matrices. Transmission via food, direct contact, and environmental contamination creates a continuous cross-sectoral challenge.
2. Convergence of virulence and resistance: The pathogen’s potent virulence factors (e.g., urease, swarming motility, robust biofilm) facilitate both severe infection and the persistence and spread of acquired AMR genes (e.g., ESBLs, carbapenemases) across all hosts.
3. Unified genetic pool of resistance: Mobile genetic elements carrying identical resistance determinants (e.g., *bla*_CTX-M_, *bla*_NDM_) are found in isolates from hospitals, farms, and food products, confirming continuous gene flow across the One Health spectrum.
4. Imperative for integrated solutions: Controlling resistant *P. mirabilis* demands coherent, cross-sectorial strategies that break down institutional silos through combined surveillance, unified antimicrobial stewardship, and innovation in therapies targeting shared vulnerabilities.

The staggering projections of AMR—including an estimated 92 million associated deaths worldwide by 2050 [397]—underscore the critical urgency of translating this knowledge into decisive, integrated action. While legislative models like the PASTEUR Act aim to stimulate antibiotic development [397,398], concerns persist regarding the sustainability of a strategy focused primarily on new drug discovery. Therefore, durable defense must be anchored in the full implementation of foundational One Health measures: eliminating antibiotics for growth promotion in livestock; reducing environmental contamination; and enforcing regulations to curb non-prescription antibiotic use [398,399,400]—a policy proven effective in settings like Saudi Arabia and Kerala, India [399,400]. The lag in adopting these evidence-based stewardship interventions remains a critical implementation gap.

Ultimately, achieving sustainable mitigation requires a multi-pronged strategy that strategically marries innovation with foundational stewardship. This involves fostering the development of alternative therapies and precision diagnostics—while diligently addressing translational challenges [401]—alongside robust, preemptive policies. Looking ahead, the path forward must be channeled into three concrete, integrated fronts:

1. Advancing Integrated, Genomic-Driven Surveillance: Establishing active, real-time genomic surveillance networks across human, veterinary, and environmental sectors is paramount for detecting emerging resistance and tracking high-risk clones across the One Health continuum.
2. Accelerating Targeted and Innovative Therapies: Prioritizing the development and translation of precision interventions, including anti-virulence compounds, phage-antibiotic combinations, novel anti-biofilm biomaterials, and nano-formulations (e.g., CSNPs), to bridge the gap between preclinical promise and clinical application.
3. Forging Enforceable, Integrated Policy Frameworks: Implementing unified international regulations on antibiotic use in agriculture, harmonized AMR data standards, and stringent environmental controls to limit the discharge of resistant bacteria, recognizing that the broad host range of *P. mirabilis* makes this integration non-negotiable.

In conclusion, *P. mirabilis* serves as a critical test for the practical implementation of the One Health paradigm. Our collective, coordinated response across these fronts will determine our ability to safeguard antimicrobial efficacy and secure the health of interconnected human, animal, and environmental ecosystems.

## Figures and Tables

**Table 1:** Key virulence factors in *P. mirabilis* and their pathogenic significance.

Key Virulence Factor	Primary Function/Role in Pathogenesis	Clinical/Pathogenic Significance
Urease	Hydrolyzes urea to ammonia and carbon dioxide, leading to local pH increase.	Promotes struvite stone formation, catheter encrustation, and tissue damage, and provides a protected niche for persistent infection.
Hemolysin (*hmp*A)	Pore-forming toxin that lyses erythrocytes and other host cells.	Facilitates iron acquisition, tissue invasion, and evasion of cellular immune defenses.
Swarming motility	Differentiation into elongated, hyperflagellated swarmer cells for rapid, coordinated surface migration.	Enhances rapid urinary tract and catheter colonization and is coupled with increased expression of other virulence factors.
Fimbriae and adhesion	MR/P and PMF fimbriae mediate specific adhesion to uroepithelial cells and abiotic surfaces.	Critical for colonization, biofilm initiation, and establishment of persistent UTI.
Biofilm formation	Production of extracellular polymeric substances (EPS) forming structured, surface-attached communities.	Confers antibiotic tolerance and protection from host immunity; hallmark of device-associated and chronic infections.
Protease (*zap*A)	Zinc-dependent metalloprotease degrading immunoglobulins (IgA, IgG) and complement factors.	Weakens humoral immunity and supports bacterial survival and persistence within the host.

**Table 2:** Mechanisms of horizontal gene transfer driving antimicrobial resistance dissemination in *P. mirabilis* and related bacteria within a One Health framework.

Mechanism	Brief Description	Key Genetic Elements Transferred	Primary Role in AMR Spread and Notes for *P. mirabilis*	One Health Relevance (Human–Animal–Environment Interface)
Conjugation	Direct, contact-dependent transfer of DNA via a conjugative pilus or other cell-surface machinery.	Plasmids (e.g., IncF, IncA/C, IncL/M types) Integrative Conjugative Elements (ICEs) Conjugative transposons	The most clinically significant mechanism. Enables rapid transfer of MGEs carrying multiple ARGs, facilitating co-resistance.	Facilitates rapid dissemination of multidrug resistance plasmids across human, animal, and environmental bacterial populations, particularly in livestock, food chains, and wastewater systems.
Transformation	Uptake and genomic integration of free extracellular DNA (eDNA) from the environment.	Naked DNA fragments (may contain gene cassettes or single genes).	Allows acquisition of resistance traits from lysed neighboring cells; efficiency varies among species and environmental conditions.	Enables environmental bacteria to act as reservoirs of resistance genes originating from human or animal sources, especially in soil, aquatic ecosystems, and biofilms.
Transduction	Bacteriophage-mediated transfer of bacterial DNA packaged within a viral capsid.	DNA packaged in phage capsids; may carry chromosomal or plasmid segments.	Supports persistence and circulation of resistance genes within microbial communities; less prominent than plasmid-mediated transfer.	Contributes to the circulation and long-term maintenance of resistance genes across diverse ecological niches, including animal microbiota and environmental reservoirs.

**Table 3:** Comprehensive overview of antimicrobial resistance mechanisms in *P. mirabilis*: Molecular determinants, genetic epidemiology, and One Health implications.

Antibiotic Class	Primary Resistance Mechanism(s)	Associated Genes/Determinants	Key Reporting Studies/Sources (in *P. mirabilis*)	Common Reservoirs/Sources (One Health)
β-Lactams	Enzymatic hydrolysis by ESBLs and pAmpC	*bla*_CTX-M_, *bla*_TEM_, *bla*_SHV_, *bla*_CMY_, *bla*_DHA_	[19,79,170,174,175,178,184,267,268]	Human hospitals, community infections, food-producing animals
Carbapenems	Enzymatic hydrolysis by Carbapenemases. Combined mechanisms: Porin loss combined with ESBL/ampC production.	*bla*_NDM_, *bla*_KPC_, *bla*_OXA-48_, *bla*_VIM_; mutations/loss of *omp*C, *omp*F	[79,170,171,220,268,308]	Hospital outbreaks; emerging in livestock and poultry. A major global health threat.
Fluoroquinolones	Target modification (QRDR). Target plasmid-mediated protection (*qnr* proteins). efflux pumps	*gyr*A, *par*C, *qnr*A/*qnr*D/*qnr*S, *aac(6′)-Ib-cr*	[79,282,283,284,285,286,287,288,291]	Human and veterinary clinics; food chain
Aminoglycosides	Enzymatic modification by AMEs enzymes	*aad*A1, *aad*A2, *aac(6′)-Ib*, *aph(3′)-Ia*, *rmt*B	[79,291,298,299,300,301,302,303,304]	Human and animal isolates via mobile genetic elements
Tetracyclines/Tigecycline	Active efflux systems	*tet*(A), *tet*(B), *tet*(M), *AcrAB-TolC*	[79,292,293,294,295,296,297]	Found across all sectors. Human, veterinary, and environmental reservoirs
Polymyxins (Colistin)	Intrinsic resistance via lipid A modification target in LPS.	Intrinsic trait; rare *mcr* genes	[57,79,170,171,220]	Natural resistance limits clinical use

**Table 4:** Examples of nanoformulations and nanocomposites evaluated against MDR *P. mirabilis*.

Nanomaterial/Formulations	Key Findings Against *P. mirabilis*	Mechanism/Synergy	Reference
CS 1% and 2%	Direct antimicrobial activity at high concentrations (MIC: 3.25–4.5 mg/mL). Moderate inhibition of swarming motility	Electrostatic binding to cell wall; membrane disruption; inhibition of DNA/RNA synthesis.	[79]
CSNPs	Exceptional potency: very low MIC (0.067–0.081 mg/mL) against extensively- and pan-drug resistant strains. Strong inhibition of swarming motility at low doses.	Enhanced membrane disruption via high surface charge/area; intracellular targeting; efflux pump inhibition	[79]
CSNPs + CIP	MIC: 0.067–0.081 mg/mL. Synergistic increase in zone of inhibition	Model of revitalizing critical antibiotics, membrane permeabilization by CSNPs facilities CIP uptakes; synergistic disruption of DNA gyrase and cell integrity Membrane disruption + anti-virulence (Swarming inhibition)	[79]
Trimethoprim nanoemulsion	8-fold reduction in MIC compared to pure drug	Enhanced drug bioavailability/penetration	[386]
Curcumin-Silver NPs	MIC range: 0.024–0.049 mg/mL	Synergistic reactive oxygen species production + metal toxicity. Protein/cell wall disruption	[387]
Silver/Copper NPs	Inhibitory activity at 250–500 µL	Oxidative stress and enzymatic inactivation	[389]
Zinc Nanoparticles	Zone of inhibition: 12 ± 1.2 mm at 200 µg/disk	Induction of intracellular reactive oxygen species Enzyme inhibition	[390]
Se/CS/AMP composite	MIC of enhanced activity: 0.1 mg/mL Vs. MIC of SeNPs alone: 0.15 mg/mL	Synergistic cationic surface interaction	[391]

## Data Availability

The original contributions presented in this study are included in the article. Further inquiries can be directed to the corresponding authors.

## References

[1] Yong T.S.M., Panting A.J., Juatan N., Perialathan K., Ahmad M., Ahmad Sanusi N.H., Hassan L., Jahis R., Shamsudin N., Yap S.L. (2021). Development and validation of a cognitive, affective and behaviour questionnaire on pet-associated zoonotic diseases (CAB-ZDQ). Vet. Med. Sci..

[2] Kirk M.D., Pires S.M., Black R.E., Caipo M., Crump J.A., Devleesschauwer B., Döpfer D., Fazil A., Fischer-Walker C.L., Hald T. (2015). World Health Organization estimates of the global and regional disease burden of 22 foodborne bacterial, protozoal, and viral diseases, 2010: A data synthesis. PLoS Med..

[3] WHO (2020). Antibiotic Resistance. https://www.who.int/news-room/fact-sheets/detail/antibiotic-resistance.

[4] van Wagenberg C.P., Havelaar A.H. (2023). Economic costs related to foodborne disease in Burkina Faso and Ethiopia in 2017. Front. Sustain. Food Syst..

[5] van Wagenberg C.P., Delele T.G., Havelaar A.H. (2022). Patient-related healthcare costs for diarrhoea, Guillain Barré syndrome and invasive non-typhoidal salmonellosis in Gondar, Ethiopia, 2020. BMC Public Health.

[6] World Bank Food-Borne Illnesses Cost US$ 110 Billion per Year in Low- and Middle-Income Countries (World Bank, 2018). https://www.worldbank.org/en/news/press-release/2018/10/23/food-borne-illnesses-cost-us-110-billion-per-year-in-low-and-middle-income-countries.

[7] Ergocun G. Unsafe Food Costs $110B Annually (World Bank. AaComTr, 2018). https://share.google/XZNcjEMf7ujwTXzB4..

[8] Hoffmann S., Ahn J.-W. (2021). Updating Economic Burden of Foodborne Disease Estimates for Inflation and Income Growth.

[9] WHO Estimating the Burden of Foodborne Diseases: A Practical Handbook for Countries: A Guide for Planning, Implementing and Reporting Country-Level Burden of Foodborne Disease (WHO, 2021). https://iris.who.int/bitstream/handle/10665/341634/9789240012264-eng.pdf?sequence=1.

[10] Najim H.T., Farhan A.A., Athab A.M. (2018). Bacteriological Study of the Bacteria Cause Urinary Tract Infection of Patients Admitted to Cardiac Care Unite a Baqubah General Teaching Hospital. DJM.

[11] Adeolu M., Alnajar S., Naushad S., Gupta R.S. (2016). Genome-based phylogeny and taxonomy of the ‘*Enterobacteriales’*: Proposal for *Enterobacterales* ord. nov. divided into the families *Enterobacteriaceae*, *Erwiniaceae* fam. Nov., *Pectobacteriaceae* fam. Nov., *Yersiniaceae* fam. Nov., *Hafniaceae* fam. Nov., *Morganellaceae* fam. Nov., and *Budviciaceae* fam. *Nov*. Int. J. Syst. Evol. Microbiol..

[12] Chakkour M., Hammoud Z., Farhat S., El Roz A., Ezzeddine Z., Ghssein G. (2024). Overview of *Proteus mirabilis* pathogenicity and virulence. Insights into the role of metals. Front. Microbiol..

[13] Armbruster C., Mobley H. (2012). Merging mythology and morphology: The multifaceted lifestyle of Proteus mirabilis. Nat. Rev. Microbiol..

[14] O’Hara C.M., Brenner F.W., Miller J.M. (2000). Classification, identification, and clinical significance of Proteus, Providencia, and Morganella. Clin. Microbiol. Rev..

[15] Kearns D.B., Losick R. (2003). Swarming motility in undomesticated *Bacillus subtilis*. Mol. Microbiol..

[16] Rather P.N. (2005). Swarmer cell differentiation in Proteus mirabilis. Environ. Microbiol..

[17] Jamil R.T., Foris L.A., Snowden J. (2024). Proteus mirabilis infections. StatPearls [Internet].

[18] Armbruster C.E., Mobley H.L., Pearson M.M. (2018). Pathogenesis of Proteus mirabilis infection. EcoSal Plus.

[19] Hassuna N.A., Kotb D.N., Lami M., Abdelrahim S.S. (2025). Characterization of antimicrobial resistance among Proteus mirabilis isolates from catheter-associated urinary tract infections and non-catheter-associated urinary tract infections in Egypt. BMC Infect. Dis..

[20] Schaffer J.N., Pearson M.M., Mulvey M.A., Klummp D.J., Stapleton A.E. (2017). Proteus mirabilis and urinary tract infections. Urinary Tract Infections: Molecular Pathogenesis and Clinical Management.

[21] Gonz’alez M.J., Navarro N., Cruz E., S’anchez S., Morales J.O., Zunino P., Robino L., Lima A., Scavone P. (2024). First report on the physicochemical and proteomic characterization of Proteus mirabilis outer membrane vesicles under urine-mimicking growth conditions. Front. Microbiol..

[22] Drzewiecka D. (2016). Significance and roles of *Proteus* spp. Bacteria in natural environments. Microb. Ecol..

[23] Armbruster C.E., Prenovost K., Mobley H.L.T., Mody L. (2016). How Often Do Clinically Diagnosed Catheter-Associated Urinary Tract Infections in Nursing Home Residents Meet Stan dardized Criteria?. J. Am. Geriatr. Soc..

[24] Hunt B.C., Brix V., Vath J., Guterman L.B., Taddei S.M., Learman B.S., Brauer A.L., Shen S., Qu J., Armbruster C.E. (2024). Metabolic interplay between *Proteus mirabilis* and *Enterococcus faecalis* facilitates polymicrobial biofilm formation and invasive disease. mBio.

[25] Daniels K.R., Lee G.C., Frei C.R. (2014). Trends in catheter-associated urinary tract infections among a national cohort of hospitalized adults, 2001-2010. Am. J. Infect. Control..

[26] Schaffer J., Pearson M. (2015). Proteus mirabilis and urinary tract infections. Microbiol. Spectr..

[27] Yang A., Tian Y., Li X. (2024). Unveiling the hidden arsenal: New insights into *Proteus mirabilis* virulence in UTIs. Front. Cell. Infect. Microbiol..

[28] Medina M., Castillo-Pino E. (2019). An introduction to the epidemiology and burden of urinary tract infections. Ther. Adv. Urol..

[29] Foxman B., Brown P. (2003). Epidemiology of urinary tract infections: Transmission and risk factors, incidence, and costs. Infect. Dis. Clin. N. Am..

[30] Norsworthy A., Pearson M. (2017). From catheter to kidney stone: The uropathogenic lifestyle of Proteus mirabilis. Trends Microbiol..

[31] Lubart E., Segal R., Haimov E., Dan M., Baumoehl Y., Leibovitz A. (2011). Bacteremia in a multilevel geriatric hospital. J. Am. Med. Dir. Assoc..

[32] Chen S.L., Kang Y.T., Liang Y.H., Qiu X.T., Li Z.J. (2023). A core genome multilocus sequence typing scheme for Proteus mirabilis. Br. Ecol. Soc..

[33] Fitzgerald M.J., Pearson M.M., Mobley H.L. (2024). Proteus mirabilis UreR coordinates cellular functions required for urease activity. J. Bacteriol..

[34] Acharya S., Kushwaha P., Desai S., Biswas S., Longjam L.A., Chatterjee B., Ruwali M., De S., Krishnaswamy L., Suravajhala P. (2025). Proteus mirabilis entails a switch from commensal to pathogen-Genomic insights from a blaTEM-1B-harboring novel isolate from India. bioRxiv.

[35] Kushwaha K., Babu D., Juneja V.K. (2014). Proteus. Encyclopedia of Food Microbiology.

[36] Gong Z., Shi X., Bai F., He X., Zhang H., Li Y., Wan Y., Lin Y., Qiu Y., Chen Q. (2019). Characterization of a Novel Diarrheagenic Strain of Proteus mirabilis Associated with Food Poisoning in China. Front. Microbiol..

[37] Wang Y., Zhang S., Yu J., Zhang H., Yuan Z., Sun Y., Zhang L., Zhu Y., Song H. (2010). An outbreak of Proteus mirabilis food poisoning associated with eating stewed pork balls in brown sauce, Beijing. Food Control.

[38] Mistry R.D., Scott H.F., Alpern E.R., Zaoutis T.E. (2010). Prevalence of Proteus Mirabilis in Skin Abscesses of the Axilla. J. Pediatr..

[39] Zhang J., Hoedt E.C., Liu Q., Berendsen E., Teh J.J., Hamilton A., O’ Brien A.W., Ching J.Y.L., Wei H., Yang K. (2021). Elucidation of Proteus Mirabilis as a Key Bacterium in Crohn’s Disease Inflammation. Gastroenterology.

[40] Dong G., Yang G., Jiang X., Zhang S., Wang Y., Hr J., Tao D. (2022). Isolation and Identification of Proteus Mirabilis from Diarrhea Lamb in Xinjiang and Its Pathogenicity in Mice. J. Anim. Sci. Vet. Med..

[41] Kitamoto S., Nagao-Kitamoto H., Jiao Y., Gillilland M.G., Hayashi A., Imai J., Sugihara K., Miyoshi M., Brazil J.C., Kuffa P. (2020). The Intermucosal Connection between the Mouth and Gut in Commensal Pathobiont-Driven Colitis. Cell.

[42] Fan C., Li X., Wu X., Ni K. (2021). Etiological characteristics and clinical features analysis of 486 pediatric diarrhea cases caused by Proteus mirabilis. Chin. J. Rural. Med. Pharm..

[43] Gao J., Liu S., Bano S., Xia X., Baloch Z. (2024). First Report of Complete Genome Analysis of Multiple Drug Resistance Proteus mirabilis KUST-1312 Isolate from Migratory Birds in China: A Public Health Threat. Transbound. Emerg. Dis..

[44] Selmi R., Tayh G., Srairi S., Mamlouk A., Ben Chehida F., Lahmar S., Bouslama M., Daaloul-Jedidi M., Messadi L. (2022). Prevalence, risk factors and emergence of extended-spectrum β-lactamase producing-, carbapenem- and colistin-resistant Enterobacterales isolated from wild boar (*Sus scrofa*) in Tunisia. Microb. Pathog..

[45] Eliopulos N., Alsina L., Diana L., Brandl S. (2022). Multiple antibiotic resistance in Enterobacteriaceae isolated from a Sea Lion (*Otaria f lavescens*) specimen from Isla de Lobos, Uruguay: A case report. Braz. J. Anim. Environ. Res..

[46] Chen Z., Wang J., Wang K., An F., Liu S., Yan H., Hua Y. (2025). Multidrug-resistant Proteus mirabilis in a critically endangered Malayan pangolin: Clinical and genomic insights. Front. Vet. Sci..

[47] Ergunay K., Mutinda M., Bourke B., Justi S.A., Caicedo-Quiroga L., Kamau J., Mutura S., Akunda I.K., Cook E., Gakuya F. (2022). Metagenomic Investigation of Ticks from Kenyan Wildlife Reveals Diverse Microbial Pathogens and New Country Pathogen Records. Front. Microbiol..

[48] Liu S., Zheng W., Huang H., Liu S., Yang M., Yan X., Li Y., Yue C., Hou R., Zhang D. (2023). Establishment and application of multiplex PCR detection method for *Proteus mirabilis*, *Klebsiella pneumoniae* and *Escherichia coli* from giant panda. Chin. J. Prev. Vet. Med..

[49] Baek S.M., Lee S.W., Lee A.R., Bang J.S., Seo M.M., Oh T., Choi S.K., Park S.J., Hong I.H., Kim T.H. (2019). Septicaemia due to a Proteus infection in a Humboldt penguin (*Spheniscus humboldti*). Vet. Med..

[50] Yu W., He Z., Huang F. (2015). Multidrug-Resistant Proteus mirabilis Isolated from Newly Weaned Infant Rhesus Monkeys and Ferrets. Jundishapur J. Microbiol..

[51] Gu W., Wang W., Tong P., Liu C., Jia J., Lu C., Han Y., Sun X., Kuang D., Li N. (2020). Comparative Genomic Analysis of Proteus Spp. Isolated from Tree Shrews Indicated Unexpectedly High Genetic Diversity. PLoS ONE.

[52] Chinnam B.K., Nelapati S., Tumati S.R., Bobbadi S., Peddada V.C., Bodempudi B. (2021). Detection of β-Lactamase-Producing Proteus mirabilis Strains of Animal Origin in Andhra Pradesh, India and Their Genetic Diversity. J. Food Prot..

[53] Qu X., Zhou J., Huang H., Wang W., Xiao Y., Tang B., Liu H., Xu C., Xiao X. (2022). Genomic Investigation of Proteus mirabilis Isolates Recovered from Pig Farms in Zhejiang Province, China. Front. Microbiol..

[54] Costinar L., Herman V., Pitoiu E., Iancu I., Degi J., Hulea A., Pascu C. (2022). Boar Semen Contamination: Identification of Gram-Negative Bacteria and antibiotic Resistance Profile. Animals.

[55] Ge Q., Ma D., Zhou Y., Chen H., Yuan J., Li X., Wang X. (2021). Isolation, Identification and Biological Characteristics of Proteus mirabilis of Swine. China Anim. Husb. Vet. Med..

[56] Sun Y., Wen S., Zhao L., Xia Q., Pan Y., Liu H., Wei C., Chen H., Ge J., Wang H. (2020). Association among biofilm formation, virulence gene expression, and antibiotic resistance in Proteus mirabilis isolates from diarrhetic animals in Northeast China. BMC Vet. Res..

[57] Ramatla T., Ramaili T., Lekota K., Mileng K., Ndou R., Mphuthi M., Khasapane N., Syakalima M., Thekisoe O. (2024). Antibiotic resistance and virulence profiles of Proteus mirabilis isolated from broiler chickens at abattoir in South Africa. Vet. Med. Sci..

[58] Marques C., Belas A., Aboim C., Trigueiro G., Cavaco-Silva P., Gama L.T., Pomba C. (2019). Clonal Relatedness of Proteus Mirabilis Strains Causing Urinary Tract Infections in Companion Animals and Humans. Vet. Microbiol..

[59] Liu L., Dong Z., Ai S., Chen S., Dong M., Li Q., Zhou Z., Liu H., Zhong Z., Ma X. (2023). Virulence-related factors and antibiotic resistance in Proteus mirabilis isolated from domestic and stray dogs. Front. Microbiol..

[60] Nasser H.A., Abbas B.A., Al-Deewan A.B. (2025). Isolation and Identification of Proteus mirabilis from human and pets urine. Microbe Infect. Dis..

[61] Pathirana H.N.K.S., De Silva B.C.J., Wimalasena S.H.M.P., Hossain S., Heo G.J. (2018). Comparison of virulence genes in Proteus species Isolated from human and pet turtle. Iran. J. Vet. Res..

[62] Fonseca J.D., Mavrides D.E., Graham P.A., McHugh T.D. (2021). Results of urinary bacterial cultures and antibiotic susceptibility testing of dogs and cats in the UK. J. Small Anim. Pract..

[63] Moyaert H., Morrissey I., De Jong A., El Garch F., Klein U., Ludwig C., Thiry J., Youala M. (2017). Antibiotic Susceptibility Monitoring of Bacterial Pathogens Isolated from Urinary Tract Infections in Dogs and Cats across Europe: ComPath Results. Microb. Drug Resist..

[64] Amphaiphan C., Yano T., Som-in M., Kungwong P., Wongsawan K., Pusoonthornthum R., Salman M.D., Tangtrongsup S. (2021). Antibiotic drug resistance profile of isolated bacteria in dogs and cats with urologic problems at Chiang Mai University Veterinary Teaching Hospital, Thailand (2012–2016). Zoonoses Public Health.

[65] Song J., Ruan H., Yang H., Mu H., Jin Y., Wang Z., Ge Y., Zheng J. (2021). Study on identification, biological characteristic of Proteus mirabilis isolated from canine and its ability to induced calculus formation. Heilongjiang Anim. Sci. Vet. Med..

[66] Wang D., Kong L., Dong W., Liu S., Gao Y., Ma H., Luan W. (2020). Isolation identification and biological characteristics of Proteus mirabilis of canine. Heilongjiang Anim. Sci. Vet. Med..

[67] Sui Z. (2022). Isolation, Identification and Drug Susceptibility of Proteus mirabilis Isolates from Dogs in Weifang City. Chin. J. Anim. Infect. Dis..

[68] Anifowose O.R., Oladosu G.A., Omotosho O.O. (2024). Occurrence and characterization of Proteus mirabilis from infected farmed African catfish in Ogun State, Nigeria. Mol. Biol. Rep..

[69] Anifowose O.R., Obisesan O.M., Adeoye B.O. (2024). Antimicrobial resistance and pathological impacts of Proteus mirabilis Infection in African Catfish (*Clarias gariepinus*) Juveniles. SVU Int. J. Vet. Sci..

[70] Rahman A.N.A., Elshopakey G.E., Alsaqufi A.S., Mansour A.T., Alkhamis Y., Hassanien H.A., Abbas A., El-Murr A., Ibrahim R.E., Mansour M.F. (2025). Naringenin alleviates Proteus mirabilis-triggered biochemical disruptions, histopathological shifts, and oxidative stress in Nile tilapia. Aquac. Rep..

[71] Apines-Amar M.J.S., Caipang C.M.A., Lopez J.D.M., Murillo M.N.A., Amar E.C., Piñosa L.A.G., Pedroso F.L. (2022). *Proteus mirabilis* (MJA 2. 6S) from saline-tolerant tilapia exhibits potent antagonistic activity against *Vibrio* spp., enhances immunity, controls NH3 levels and improves growth and survival in juvenile giant tiger shrimp, *Penaeus monodon*. Aquac. Res..

[72] Tripathy P.S., Parida S.N., Tyagi A., Rout A.K., Behera B.K., Pandey P.K. (2025). Whole-genome sequencing and assembly of fish pathogenic Proteus mirabilis (COFI-RLBCAU-II) possessing multiple antimicrobial resistance genes. Microbiol. Resour. Announc..

[73] Shammah V.B., Mailafia S., Ameh J.A., Audu B.J., Jagab H., Gideon J. (2024). Molecular detection of virulence genes and Extended Spectrum Beta-lactam producing enzymes of Proteus mirabilis isolates from fishes in Federal Capital Territory (FCT) Abuja, Nigeria. Mansoura Vet. Med. J..

[74] Oghenochuko M.O., Ola E.I., Thomas M.R., Daodu O.G., Oguntuase G.A., Aluko O.I., Irokanulo E., Akpor B.O. (2024). Effects of Single and Co-infections of Proteus Mirabilis and Aeromonas Hydrophila on Baseline Hematological, Serological, and Histological Data in Cultured Clarias Gariepinus. Open Agric. J..

[75] Mohammadi M.R., Kiaheyrati N., Khakpour M., Fardsanei F., Hoseini F.N., Sobhani S., Zavaraki M.F., Ejlali G.K., Alijani N., Nikkhahi F. (2025). Virulence and Antibiotic Resistance Profiles of Proteus mirabilis Strains Isolated From Broiler Chickens: Implications for Poultry and Public Health. Vet. Med. Sci..

[76] Sanches M.S., Rodrigues da Silva C., Silva L.C., Montini V.H., Lopes Barboza M.G., Migliorini Guidone G.H., Dias de Oliva B.H., Nishio E.K., Faccin Galhardi L.C., Vespero E.C. (2021). Proteus mirabilis from community-acquired urinary tract infections (UTI-CA) shares genetic similarity and virulence factors with isolates from chicken, beef and pork meat. Microb. Pathog..

[77] Edris S.N., Hamad A., Awad D.A.B., Sabeq I.I. (2023). Prevalence, antibiotic resistance patterns, and biofilm formation ability of Enterobacterales recovered from food of animal origin in Egypt. Vet. World.

[78] Algammal A.M., Hashem H.R., Alfifi K.J., Hetta H.F., Sheraba N.S., Ramadan H., El-Tarabili R.M. (2021). AtpD Gene Sequencing, Multidrug Resistance Traits, Virulence-Determinants, and Antimicrobial Resistance Genes of Emerging XDR and MDR-Proteus Mirabilis. Sci. Rep..

[79] Hasona I.F., Awad A., Younis G., Mohamed W.F. (2025). Effectiveness of Chitosan and Its Nanoparticles Against ampC- and ESBL-Producing Pan-Drug-Resistant *Proteus mirabilis* in Egyptian Livestock. Pathogens.

[80] Yu Z., Joossens M., Van den Abeele A.M., Kerkhof P.J., Houf K. (2021). Isolation, characterization and antibiotic resistance of Proteus mirabilis from Belgian broiler carcasses at retail and human stool. Food Microbiol..

[81] Zhu X., Zhang Y., Shen Z., Xia L., Wang J., Zhao L., Wang K., Wang W., Hao Z., Liu Z. (2021). Characterization of NDM-1-producing carbapenemase in *Proteus mirabilis* among broilers in China. Microorganisms.

[82] Ma W.Q., Han Y.Y., Zhou L., Peng W.Q., Mao L.Y., Yang X., Wang Q., Zhang T.J., Wang H.N., Lei C.W. (2022). Contamination of Proteus mirabilis harbouring various clinically important antibiotic resistance genes in retail meat and aquatic products from food markets in China. Front. Microbiol..

[83] Lan C., Hu Y., Wang M., Li C., Bi S. (2023). Prevalence and Antibiotics Resistance of Proteus mirabilis in Raw Meat. Sci. Technol. Food Ind..

[84] Liu Y., Liu L., Li H., Liu N., Liu Y., Du J. (2022). Quantitative PCR Detection and Drug Resistance Analysis of Proteus mirabilis in Fresh Chicken meat. Agric. Prod. Process.

[85] Zaher A.H., Kabadaia M.M., Hammad K.M., Mekky A.E., Salem S.S. (2023). Forensic flies as carries of pathogenic bacteria associated with a pig carcass in Egypt. Al-Azhar Bull. Sci..

[86] Liu X.-L., Wu S.-Y., Yu Z. (2025). Zoonotic Risks of Proteus mirabilis: Detection, Pathogenicity, and Antibiotic Resistance in Animals and Animal-Derived Foods. Microorganisms.

[87] Zhai Q., Zhai S., Lv Y., Wen X., Wu G., Huo W., Tu D., Wei W., Jia C., Zhou X. (2021). Isolation, Identification and Drug Resistance of Proteus mirabilis from Bamboo Rats. Chin. J. Anim. Infect. Dis..

[88] Wang J., Li S., Zhang Z., Wang S., Wei L., Zhu X., Liu Y. (2024). Isolation, identification and pathogenicity analysis of Proteus mirabilis from rabbits. Chin. J. Prev. Vet. Med..

[89] Wang J., Xu J., Jin H., Hou S., Feng J. (2019). Isolation, Identification and Drug Resistance Analysis of Rabbit Proteus mirabilis. China Anim. Husb. Vet. Med..

[90] Qin S., He C., Li C., Zeng W., Ma L., Liu J., Bai A., Yang L., Wu J. (2023). Isolation, and identification and biological-charactistics of swine-sourced *Proteus mirabils* producing AmpC enzyme. Chin. J. Anim. Infect. Dis..

[91] Chen H., Ma L., Qin S., Chen Y., Song R., Sun Q., Qin S., Liu J., Chen F., Wu J. (2023). Isolation, identification and biological characteristics of swine-sourced Proteus mirabilis producing AmpC enzyme. Anim. Husb. Vet. Med..

[92] Saif Y.M. (2008). Diseases of Poultry. Other Bacterial Diseases: Blackwell Publishing Professional.

[93] Abdollahi M., Javan A.J., Shokrpoor S., Beidokhtinezhad M., Tamai I.A. (2022). Pyoderma Caused by Proteus Mirabilis in Sheep. Vet. Med. Sci..

[94] Najd Ghahremani A., Abdollahi M., Shokrpoor S., Ashrafi Tamai I. (2023). Pericarditis Caused by Proteus Mirabilis in Sheep. Vet. Med. Sci..

[95] Zhang S., Li Q., Wang M., Jia R., Chen S., Liu M., Zhu D., Zhao X., Wu Y., Yang Q. (2025). Genomic analysis of Proteus mirabilis: Unraveling global epidemiology and antimicrobial resistance dissemination—Emerging challenges for public health and biosecurity. Environ. Int..

[96] Costa T., Linhares I., Ferreira R., Neves J., Almeida A. (2018). Frequency and Antibiotic Resistance of Bacteria Implicated in Community Urinary Tract Infections in North Aveiro between 2011 and 2014. Microb. Drug Resist..

[97] Herout R., Khoddami S., Moskalev I., Reicherz A., Chew B.H., Armbruster C.E., Lange D. (2023). Role of Bacterial Surface Components in the Pathogenicity of *Proteus mirabilis* in a Murine Model of Catheter Associated Urinary Tract Infection. Pathogens.

[98] Danilo de Oliveira W., Lopes Barboza M., Faustino G., Yamanaka Inagaki W., Sanches M., Takayama Kobayashi R., Vespero E.C., Dejato Rocha S.P. (2021). Virulence, resistance and clonality of Proteus mirabilis isolated from patients with community-acquired urinary tract infection (CA-UTI) in Brazil. Microb. Pathog..

[99] Beynon L., Dumanski A., McLean R., MacLean L., Richards J., Perry M. (1992). Capsule structure of Proteus mirabilis (ATCC 49565). J. Bacteriol..

[100] Rozalski A., Torzewska A., Moryl M., Kwil I., Maszewska A., Ostrowska K., Drzewiecka D., Zablotni A., Palusak A., Siwinska M. (2012). *Proteus* sp. An opportunistic bacterial pathogen classification, swarming growth, clinical significance and virulence factors. Folia Biol. Oecologica.

[101] Kappaun K., Piovesan A., Carlini C., Ligabue-Braun R. (2018). Ureases: Historical aspects, catalytic, and non-catalytic properties—A review. J. Adv. Res..

[102] Wasfi R., Hamed S.M., Amer M.A., Fahmy L.I. (2020). Proteus mirabilis biofilm: Development and therapeutic strategies. Front. Cell. Infect. Microbiol..

[103] Chew R., Thomas S., Mantha M., Killen J., Cho Y., Baer R. (2012). Large urate cystolith associated with Proteus urinary tract infection. Kidney Int..

[104] Armbruster C., Smith S., Yep A., Mobley H. (2014). Increased incidence of urolithiasis and bacteremia during Proteus mirabilis and *Providencia stuartii* coinfection due to synergistic induction of urease activity. J. Infect. Dis..

[105] Follmer C. (2010). Ureases as a target for the treatment of gastric and urinary infections. J. Clin. Pathol..

[106] Al-Fahham H.R.A., Kareem K.R. (2022). Molecular study of urease ureR gene of Proteus mirabilis isolated from urinary tract infections, Najaf, Iraq. Arch. Razi Inst..

[107] Caldara M., Bolhuis H., Marmiroli M., Marmiroli N. (2025). Biofilm formation, modulation, and transcriptomic regulation under stress conditions in *halomicronema* sp. Int. J. Mol. Sci..

[108] Kynshi M.A.L., Kharkamni E., Borah V.V. (2025). Proteus mirabilis: Insights into biofilm formation, virulence mechanisms, and novel therapeutic strategies. Microbe.

[109] Tabatabaei A., Ahmadi K., Shabestari A.N., Khosravi N., Badamchi A. (2021). Virulence genes and antimicrobial resistance pattern in Proteus mirabilis strains isolated from patients attended with urinary infections to tertiary hospitals, in Iran. Afr. Health Sci..

[110] Mohammed G.J., Kadhim M.J., Hameed I.H. (2016). Proteus species: Characterization and herbal antibacterial: A review. Int. J. Pharmacogn. Phytochem. Res..

[111] Rocha S.P., Elias W.P., Cianciarullo A.M., Menezes M.A., Nara J.M., Piazza R.M., Silva M.R., Moreira C.G., Pelayo J.S. (2007). Aggregative adherence of uropathogenic Proteus mirabilis to cultured epithelial cells. FEMS Immunol. Med. Microbiol..

[112] Gmiter D., Kaca W. (2022). Into the understanding the multicellular lifestyle of Proteus mirabilis on solid surfaces. Front. Cell. Infect. Microbiol..

[113] Pearson M.M., Sebaihia M., Churcher C., Quail M.A., Seshasayee A.S., Luscombe N.M., Abdellah Z., Arrosmith C., Atkin B., Chillingworth T. (2008). Complete genome sequence of uropathogenic Proteus mirabilis, a master of both adherence and motility. J. Bacteriol..

[114] Kuan L., Schaffer J., Zouzias C., Pearson M. (2014). Characterization of 17 chaperone-usher fimbriae encoded by Proteus mirabilis reveals strong conservation. J. Med. Microbiol..

[115] Persat A., Inclan Y., Engel J., Stone H., Gitai Z. (2015). Type IV pili mechanochemically regulate virulence factors in Pseudomonas aeruginosa. Proc. Natl. Acad. Sci. USA.

[116] Gué M., Dupont V., Dufour A., Sire O. (2001). Bacterial swarming: A biochemical time-resolved FTIR-ATR study of Proteus mirabilis swarm-cell differentiation. Biochemistry.

[117] Fraser G., Claret L., Furness R., Gupta S., Hughes C. (2002). Swarming coupled expression of the Proteus mirabilis hpmBA haemolysin operon. Microbiology.

[118] Himpsl S.D., Lockatell C.V., Hebel J.R., Johnson D.E., Mobley H.L. (2008). Identification of virulence determinants in uropathogenic Proteus mirabilis using signature-tagged mutagenesis. J. Med. Microbiol..

[119] Prywer J., Olszynski M. (2017). Bacterially induced formation of infectious urinary stones: Recent developments and future challenges. Curr. Med. Chem..

[120] Khayyat A.N., Abbas H.A., Mohamed M.F.A., Asfour H.Z., Khayat M.T., Ibrahim T.S., Youns M., Khafagy E.-S., Abu Lila A.S., Safo M.K. (2021). Not only antimicrobial: Metronidazole mitigates the virulence of Proteus mirabilis isolated from macerated diabetic foot ulcer. Appl. Sci..

[121] Jones B.V., Young R., Mahenthiralingam E., Stickler D.J. (2004). Ultrastructure of Proteus mirabilis swarmer cell rafts and role of swarming in catheter-associated urinary tract infection. Infect. Immun..

[122] Cestari S., Ludovico M., Martins F., da Rocha S., Elias W., Pelayo J. (2013). Molecular detection of HpmA and HlyA hemolysin of uropathogenic Proteus mirabilis. Curr. Microbiol..

[123] Alamuri P., Löwer M., Hiss J., Himpsl S., Schneider G., Mobley H. (2010). Adhesion, invasion, and agglutination mediated by two trimeric autotransporters in the human uropathogen Proteus mirabilis. Infect. Immun..

[124] Belas R., Manos J., Suvanasuthi R. (2004). Proteus mirabilis ZapA metalloprotease degrades a broad spectrum of substrates, including antimicrobial peptides. Infect. Immun..

[125] Phan V., Belas R., Gilmore B., Ceri H. (2008). ZapA, a virulence factor in a rat model of Proteus mirabilis-induced acute and chronic prostatitis. Infect. Immun..

[126] Rather M.A., Gupta K., Mandal M. (2021). Microbial biofilm: Formation, architecture, antibiotic resistance, and control strategies. Braz. J. Microbiol..

[127] Chen H.H., Chang C.C., Yuan Y.H., Liaw S.J. (2020). A CpxR-regulated zapD gene involved in biofilm formation of uropathogenic Proteus mirabilis. Infect. Immun..

[128] Stankowska D., Kwinkowski M., Kaca W. (2008). Quantification of Proteus mirabilis virulence factors and modulation by acylated homoserine lactones. J. Microbiol. Immunol. Infect..

[129] Prywer J., Torzewska A., Cichomski M., Michałowski P.P. (2023). Insights into the physical and chemical properties of struvite crystal surfaces in terms of the effectiveness of bacterial adhesion. Sci. Rep..

[130] Schneider R., Lockatell C.V., Johnson D., Belas R. (2002). Detection and mutation of a luxS-encoded autoinducer in Proteus mirabilis. Microbiology.

[131] Parsek M.R., Greenberg E.P. (2005). Sociomicrobiology: The connections between quorum sensing and biofilms. Trends Microbiol..

[132] Lila A.S.A., Rajab A.A.H., Abdallah M.H., Rizvi S.M.D., Moin A., Khafagy E.S., Tabrez S., Hegazy W.A.H. (2023). Biofilm Lifestyle in Recurrent Urinary Tract Infections. Life.

[133] Rajab A., Hegazy W. (2023). What’s old is new again: Insights into diabetic foot microbiome. World J. Diabetes.

[134] Veisi M., Hosseini-Nave H., Tadjrobehkar O. (2025). Biofilm formation ability and swarming motility are associated with some virulence genes in Proteus mirabilis. BMC Microbiol..

[135] Jiang Y., Geng M., Bai L. (2020). Targeting Biofilms Therapy: Current Research Strategies and Development Hurdles. Microorganisms.

[136] Koo H., Allan R.N., Howlin R.P., Stoodley P., Hall-Stoodley L. (2017). Targeting Microbial Biofilms: Current and Prospective Therapeutic Strategies. Nat. Rev. Microbiol..

[137] Maggio F., Rossi C., Serio A., Chaves-Lopez C., Casaccia M., Paparella A. (2025). Anti-Biofilm Mechanisms of Action of Essential Oils by Targeting Genes Involved in Quorum Sensing, Motility, Adhesion, and Virulence: A Review. Int. J. Food Microbiol..

[138] Lu L., Hu W., Tian Z., Yuan D., Yi G., Zhou Y., Cheng Q., Zhu J., Li M. (2019). Developing Natural Products as Potential Anti-Biofilm Agents. Chin. Med..

[139] Soulaimani B. (2025). Comprehensive Review of the Combined Antimicrobial Activity of Essential Oil Mixtures and Synergism with Conventional Antimicrobials. Nat. Prod. Commun..

[140] Packiavathy I.A., Priya S., Pandian S.K., Ravi A.V. (2014). Inhibition of biofilm development of uropathogens by curcumin—An anti-quorum sensing agent from Curcuma longa. Food Chem..

[141] Wasfi R., Abd El-Rahman O.A., Mansour L.E., Hanora A.S., Hashem A.M., Ashour M.S. (2012). Antimicrobial activities against biofilm formed by Proteus mirabilis isolates from wound and urinary tract infections. Indian J. Med. Microbiol..

[142] Heymann D.L., Dar O.A. (2014). Prevention is better than cure for emerging infectious diseases. BMJ.

[143] Dharmarajan G., Li R., Chanda E., Dean K.R., Dirzo R., Jakobsen K.S., Khan I., Leirs H., Shi Z.-L., Wolfe N.D. (2022). The animal origin of major human infectious diseases: What can past epidemics teach us about preventing the next pandemic?. Zoonoses.

[144] Murray C.J., Ikuta K.S., Sharara F., Swetschinski L., Aguilar G.R., Gray A., Han G., Bisignano C., Rao P., Wool E. (2022). Global burden of bacterial antimicrobial resistance in 2019: A systematic analysis. Lancet.

[145] O’Neill J. (2016). Tackling Drug-Resistant Infections Globally: Final Report and Recommendations the Review on Antimicrobial Resistance.

[146] Tesema M.Y., Birhanu A.G. (2024). One health initiative to mitigate the challenge of antimicrobial resistance in the perspectives of developing countries. Bull. Natl. Res. Cent..

[147] Larsson D.G.J., Flach C.-F. (2022). Antibiotic resistance in the environment. Nat. Rev. Microbiol..

[148] Centers for Disease Control and Prevention (2019). 2019 Antibiotic Resistance Threats Report. CDC. https://stacks.cdc.gov/view/cdc/82532.

[149] Biswas R., Debnatha C., Bandyopadhyayb S., Samantac I. (2022). One Health approaches adapted in low resource settings to address antimicrobial resistance. Sci. One Health.

[150] Walsh T.R., Weeks J., Livermore D.M., Toleman M.A. (2011). Dissemination of NDM-1 positive bacteria in the New Delhi environment and its implications for human health: An environmental point prevalence study. Lancet Infect. Dis..

[151] Facciolà A., Gioffrè M.E., Chiera D., Ferlazzo M., Virga A., Laganà P. (2022). Evaluation of antibiotic resistance in *Proteus* spp: A growing trend that worries Public Health. Results of 10 Years of Analysis. New Microbiol..

[152] Vaughn V.M., Gandhi T.N., Petty L.A., Patel P.K., Prescott H.C., Malani A.N., Ratz D., McLaughlin E., Chopra V., Flanders S.A. (2021). Empiric antibacterial therapy and community-onset bacterial coinfection in patients hospitalized with coronavirus disease 2019 (COVID-19): A Multi-hospital Cohort Study. Clin. Infect. Dis..

[153] Jans C., Sarno E., Collineau L., Meile L., Stärk K.D.C., Stephan R. (2018). Consumer exposure to antimicrobial-resistant bacteria from food at Swiss retail level. Front. Microbiol..

[154] Massé D.I., Cata Saady N.M., Gilbert Y. (2014). Potential of biological processes to eliminate antibiotics in livestock manure: An overview. Animals.

[155] Kumar K., Gupta S.C., Chander Y., Singh A.K. (2005). Antibiotic use in agriculture and its impact on the terrestrial environment. Adv. Agron..

[156] Kümmerer K. (2009). Antibiotics in the aquatic environment—A review—Part I. Chemosphere.

[157] Grenni P., Ancona V., Caracciolo A.B. (2018). Ecological effects of antibiotics on natural ecosystems: A review. Microchem. J..

[158] Van Boeckel T.P., Brower C., Gilbert M., Grenfell B.T., Levin S.A., Robinson T.P., Teillant A., Laxminarayan R. (2015). Global trends in antimicrobial use in food animals. Proc. Natl. Acad. Sci. USA.

[159] Food and Agriculture Organization (FAO) (2016). Drivers, Dynamics and Epidemiology of Antimicrobial Resistance in Animal or Production. http://www.fao.org/3/ai6209e.pdf.

[160] Klein E.Y., Tseng K.K., Pant S., Laxminarayan R. (2019). Tracking global trends in the effectiveness of antibiotic therapy using the Drug Resistance Index. BMJ Glob. Health.

[161] Klein E.Y., Van Boeckel T.P., Martinez E.M., Pant S., Gandra S., Levin S.A., Goossens H., Laxminarayan R. (2018). Global increase and geographic convergence in antibiotic consumption between 2000 and 2015. Proc. Natl. Acad. Sci. USA.

[162] Chakraborty T., Barbuddhe S.B. (2021). Enabling one health solutions through genomics. Indian J. Med. Res..

[163] Van Boeckel T.P., Glennon E.E., Chen D., Gilbert M., Robinson T.P., Grenfell B.T., Levin S.A., Bonhoeffer S., Laxminarayan R. (2017). Reducing antimicrobial use in food animals. Science.

[164] OIE (2021). Annual Report on Antimicrobial Agents Intended for Use in Animals. https://www.flemingfund.org/publications/oie-publish-their-fourth-annual-report-on-antimicrobial-agents-intended-for-use-in-animals/.

[165] O’neill J.I. (2014). Antimicrobial resistance: Tackling a crisis for the health and wealth of nations. Rev. Antimicrob. Resist..

[166] OECD (2018). Stemming the Superbug Tide: Just a Few Dollars More.

[167] ECDC/EMEA Joint Technical Report. (2009). The Bacterial Challenge: Time to React.

[168] Wilke M.H. (2010). Multiresistant bacteria and current Therapy—The economical side of the story. Eur. J. Med. Res..

[169] Belay W.Y., Getachew M., Tegegne B.A., Teffera Z.H., Dagne A., Zeleke T.K., Wondm S.A., Abebe R.B., Gedif A.A., Fenta A. (2025). Antimicrobial resistance with a focus on antibacterial, antifungal, antimalarial, and antiviral drugs resistance, its threat, global priority pathogens, prevention, and control strategies: A review. Ther. Adv. Infect. Dis..

[170] Girlich D., Bonnin R.A., Dortet L., Naas T. (2020). Genetics of acquired antibiotic resistance genes in *Proteus* spp. Front Microbiol..

[171] Ejaz H., Younas S., Abosalif K.O.A., Junaid K., Alzahrani B., Alsrhani A., Abdalla A.E., Ullah M.I., Qamar M.U., Hamam S.S.M. (2021). Molecular analysis of blaSHV, blaTEM, and blaCTX-M in extended-spectrum β-lactamase producing Enterobacteriaceae recovered from fecal specimens of animals. PLoS ONE.

[172] Al-Saadi N., Alsallami D., Alsultan A., Al-hriahaw H. (2022). Whole-genomic sequence of multidrug resistance burkholderia cepacia associated with acute suppurative thyroiditis. J. Complement. Med Res..

[173] Hu R., Wang X., Muhamamd I., Wang Y., Dong W., Zhang H. (2020). Biological characteristics and genetic analysis of a highly pathogenic Proteus mirabilis strain isolated from dogs in China. Front. Vet. Sci..

[174] Bush K., Jacoby G.A. (2010). Updated functional classification of beta lactamases. Antimicrob. Agents Chemother..

[175] Pitout J.D., Nordmann P., Laupland K.B., Poirel L. (2005). Emergence of Enterobacteriaceae producing extended-spectrum β-lactamases (ESBLs) in the community. J. Antimicrob. Chemother..

[176] Adamus-Bialek W., Zajac E., Parniewski P., Kaca W. (2013). Comparison of antibiotic resistance patterns in collections of Escherichia coli and Proteus mirabilis uropathogenic strains. Mol. Biol. Rep..

[177] Ilbeigi K., Askari Badouei M., Vaezi H., Zaheri H., Aghasharif S., Kafshdouzan K. (2021). Molecular survey of mcr1 and mcr2 plasmid mediated colistin resistance genes in Escherichia coli isolates of animal origin in Iran. BMC Res. Notes.

[178] Yong D., Lim Y.S., Roh K.H., Choi Y.S., Park D.Y., Yum J.H., Kim J.M., Lee K., Chong Y. (2006). The first detection of CTX-M-14 extended-spectrum beta-lactamase among diverse beta-lactamase-producing Proteus mirabilis clinical isolates. Diagn. Microbiol. Infect. Dis..

[179] Lim E.J., Ho S.X., Cao D.Y., Lau Q.C., Koh T.H., Hsu L.Y. (2016). Extended-Spectrum Beta-Lactamase-Producingin Retail Chicken Meat in Singapore. Ann. Acad. Med. Singap..

[180] Mathers A.J., Peirano G., Pitout J.D. (2015). The role of epidemic resistance plasmids and international high-risk clones in the spread of multidrug-resistant Enterobacteriaceae. Clin. Microbiol. Rev..

[181] Partridge S.R., Kwong S.M., Firth N., Jensen S.O. (2018). Mobile genetic elements associated with antimicrobial resistance. Clin. Microbiol. Rev..

[182] Poirel L., Jayol A., Nordmann P. (2017). Polymyxins: Antibacterial activity, susceptibility testing, and resistance mechanisms encoded by plasmids or chromosomes. Clin. Microbiol. Rev..

[183] Hawser S.P., Bouchillon S.K., Hoban D.J., Badal R.E. (2011). Antibiotic susceptibility of intra-abdominal Gram-negative bacilli from Europe, North America and South America: SMART 2008. J. Antimicrob. Chemother..

[184] Trecarichi E.M., Tumbarello M. (2017). Antimicrobial-resistant Gram-negative bacteria in healthcare settings: A clinical perspective. Front. Med..

[185] Sanches M.S., Silva L.C., da Silva C.R., Montini V.H., de Oliva B.H.D., Guidone G.H.M., Nogueira M.C.L., Menck-Costa M.F., Kobayashi R.K.T., Vespero E.C. (2023). Prevalence of Antimicrobial Resistance and Clonal Relationship in ESBL/AmpC-Producing Proteus Mirabilis Isolated from Meat Products and Community-Acquired Urinary Tract Infection (UTI-CA) in Southern Brazil. Antibiotics.

[186] Launay A., Wu C.-J., Dulanto Chiang A., Youn J.-H., Khil P.P., Dekker J.P. (2021). In vivo evolution of an emerging zoonotic bacterial pathogen in an immunocompromised human host. Nat. Commun..

[187] Roberts M.C., Schwarz S., Aarts H.J. (2020). Environmental dissemination of antimicrobial resistance. Environ. Pollut..

[188] La Rosa M.C., Maugeri A., Favara G., La Mastra C., Magnano San Lio R., Barchitta M., Agodi A. (2025). The impact of wastewater on antimicrobial resistance: A scoping review of transmission pathways and contributing factors. Antibiotics.

[189] Ramanathan A.L., Sabarathinam C., Jonathan M.P., Prasanna M.V., Kumar P., Arriola F.M. (2021). Environmental Resilience and Transformation in Times of COVID-19.

[190] Bandyopadhyay S., Samanta I., Bhattacharyya D., Nanda P.K., Kar D., Chowdhury J., Dandapat P., Das A.K., Batul N., Mondal B. (2015). Co-infection of methicillin-resistant Staphylococcus epidermidis, methicillin-resistant Staphylococcus aureus and extended spectrum β-lactamase producing Escherichia coli in bovine mastitis–three cases reported from India. Vet. Q..

[191] Kar D., Bandyopadhyay S., Bhattacharyya D., Samanta I., Mahanti A., Nanda P.K., Mondal B., Dandapat P., Das A.K., Dutta T.K. (2015). Molecular and phylogenetic characterization of multidrug resistant extended spectrum beta-lactamase producing Escherichia coli isolated from poultry and cattle in Odisha, India. Infect. Genet. Evol..

[192] Koovapra S., Bandyopadhyay S., Das G., Bhattacharyya D., Banerjee J., Mahanti A., Samanta I., Nanda P.K., Kumar A., Mukherjee R. (2016). Molecular signature of extended spectrum β-lactamase producing Klebsiellapneumoniae isolated from bovine milk in eastern and north-eastern India Infect. Genet. Evol..

[193] Sarwar A., Aslam B., Mahmood S., Muzammil S., Siddique A.B., Sarwar F., Khurshid M., Rasool M.H., Sasanya J., Aljasir S.F. (2025). Distribution of multidrug-resistant *Proteus mirabilis* in poultry, livestock, fish, and the related environment: One Health heed. Vet. World.

[194] Bandyopadhyay S., Banerjee J., Bhattacharyya D., Samanta I., Mahanti A., Dutta T.K., Ghosh S., Nanda P.K., Dandapat P., Bandyopadhyay S. (2018). Genomic identity of fluoroquinolone-resistant bla CTX-M-15-type ESBL and pMAmpC β-lactamase producing Klebsiella pneumoniae from buffalo milk, India. Microb. Drug Resist..

[195] Koirala B., Bhattarai R., Maharjan R., Maharjan S., Shrestha S. (2020). Bacterial Assessment of Buffalo Meat in Kathmandu Valley. Nepal J. Sci. Technol..

[196] Mansour S.N., Youssef W., Hana Y., Elias R.S., Nagib H.E., Hakim A.S., Younis E.M., Dapgh A.N. (2023). Multidrug Resistance in Gram Negative Bacteria Isolated from Cases of Mastitis in Buffaloes. Egypt. J. Anim. Health.

[197] Samanta I., Joardar S.N., Mahanti A., Bandyopadhyay S., Sar T.K., Dutta T.K. (2015). Approaches to characterize extended spectrum beta-lactamase/beta-lactamase producing *Escherichia coli* in healthy organized vis-a-vis backyard farmed pigs in India. Infect. Genet. Evol..

[198] Samanta A., Mahanti A., Chatterjee S., Joardar S.N., Bandyopadhyay S., Sar T.K., Mandal G.P., Dutta T.K., Samanta I. (2018). Pig farm environment as a source of beta lactamase or AmpC-producing Klebsiella pneumoniae and Escherichia coli. Ann. Microbiol..

[199] Mahanti A., Ghosh P., Samanta I., Joardar S.N., Bandyopadhyay S., Bhattacharyya D., Banerjee J., Batabyal S., Sar T.K., Dutta T.K. (2018). Prevalence of CTX M-producing Klebsiella spp. in broiler, kuroiler, and indigenous poultry in West Bengal state, India. Microb. Drug Resist..

[200] El-Demerdash A.S., Aggour M.G., El-Azzouny M.M., Abou-Khadra S.H. (2018). Molecular analysis of integron gene cassette arrays associated multi-drug resistant Enterobacteriaceae isolates from poultry. Cell. Mol. Biol..

[201] Ishaq K., Ahmad A., Rafique A., Aslam R., Ali S., Shahid M.A., Sarwar N., Aslam M.A.., Aslam B., Arshad M.I. (2022). Occurrence and antimicrobial susceptibility of *Proteus mirabilis* from chicken carcass. Pak. Vet. J..

[202] Banerjee A., Bardhan R., Chowdhury M., Joardar S.N., Isore D.P., Batabyal K., Dey S., Sar T.K., Bandyopadhyay S., Dutta T.K. (2019). Characterization of beta-lactamase and biofilm producing Enterobacteriaceae isolated from organized and backyard farm ducks. Lett. Appl. Microbiol..

[203] Kyung S.M., Lee J.H., Lee E.S., Xiang X.R., Yoo H.S. (2024). Emergence and genomic characterization of Proteus mirabilis harboring bla NDM-1 in Korean companion dogs. Vet. Res..

[204] El-Tarabili R.M., Ahmed E.M., Alharbi N.K., Alharbi M.A., AlRokban A.H., Naguib D., Alhag S.K., El Feky T.M., Ahmed A.E., Mahmoud A.E. (2022). Prevalence, Antibiotic Profile, Virulence Determinants, ESBLs, and Non-β-Lactam Encoding Genes of MDR *Proteus* spp. Isolated from Infected Dogs. Front. Genet..

[205] Aziz R.K., Breitbart M., Edwards R.A. (2010). Transposases are the most abundant, most ubiquitous genes in nature. Nucleic Acids Res..

[206] Potter R.F., Zhang K., Reimler B., Marino J., Muenks C.E., Alvarado K., Wallace M.A., Westblade L.F., McElvania E., Yarbrough M.L. (2023). Uncharacterized and lineage-specific accessory genes within the Proteus mirabilis pan-genome landscape. mSystems.

[207] Booton R.D., Meeyai A., Alhusein N., Buller H., Feil E., Lambert H., Mongkolsuk S., Pitchforth E., Reyher K.K., Sakcamduang W. (2021). One Health drivers of antibacterial resistance: Quantifying the relative impacts of human, animal and environmental use and transmission. One Health.

[208] Manyi-Loh C., Mamphweli S., Meyer E., Okoh A. (2018). Antibiotic Use in Agriculture and Its Consequential Resistance in Environ mental Sources: Potential Public Health Implications. Molecules.

[209] Kapoor G., Saigal S., Elongavan A. (2017). Action and resistance mechanisms of antibiotics: A guide for clinicians. J. Anaesthesiol. Clin. Pharmacol..

[210] Laws M., Shaaban A., Rahman K.M. (2019). Antibiotic resistance breakers: Current approaches and future directions. FEMS Microbiol. Rev..

[211] Zhu Y., Huang W.E., Yang Q. (2022). Clinical perspective of antimicrobial resistance in bacteria. Infect. Drug Resist..

[212] Reygaert W.C. (2018). An overview of the antimicrobial resistance mechanisms of bacteria. AIMS Microbiol..

[213] Marti E., Variatza E., Balcazar J.L. (2014). The role of aquatic ecosystems as reservoirs of antibiotic resistance. Trends Microbiol..

[214] Jiang Z., Li P., Qiu K., Liao Y., Chen X., Xuan J., Wang F., Ma H., Wang Y., Zhu M. (2025). Proteus Mirabilis Exacerbates Ulcerative Colitis by Inhibiting Mucin Production. Front. Microbiol..

[215] Leverstein-van Hall M.A., Blok H.E.M., Donders A.R.T., Paauw A., Fluit A.C., Verhoef J. (2003). Multidrug resistance among Enterobacteriaceae is strongly associated with the presence of integrons and is independent of species or isolate origin. J. Infect. Dis..

[216] Baker S., Thomson N., Weill F.-X., Holt K.E. (2017). Genomic insights into the emergence and spread of antimicrobial-resistant pathogens. Science.

[217] Poole K. (2012). Bacterial stress response as determinants of antimicrobial resistance. J. Antimicrob. Chemother..

[218] Pal C., Asiani K., Arya S., Rensing C., Stekel D.J., Larsson D.G.K., Hobman J.L. (2017). Metal resistance and its association with antibiotic resistance. Adv. Microb. Physiol..

[219] Urban-Chmiel R., Marek A., Stępień-Pyśniak D., Wieczorek K., Dec M., Nowaczek A., Osek J. (2022). Antibiotic resistance in bacteria—A review. Antibiotics.

[220] Fritzenwanker M., Falgenhauer J., Hain T., Imirzalioglu C., Chakraborty T., Yao Y. (2025). The Detection of Extensively Drug-Resistant Proteus Mirabilis Strains Harboring Both VIM-4 and VIM-75 Metallo-β-Lactamases from Patients in Germany. Microorganisms.

[221] Aslam B., Khurshid M., Arshad M.I., Muzammil S., Rasool M., Yasmeen N., Shah T., Chaudhry T.H., Rasool M.H., Shahid A. (2021). Antibiotic resistance: One health one world outlook. Front. Cell Infect. Microbiol..

[222] Ragan M.A., Beiko R.G. (2009). Lateral genetic transfer: Open issues. Philos. Trans. R Soc. Lond. B Biol. Sci..

[223] Ochman H., Lawrence J.G., Groisman E.A. (2000). Lateral gene transfer and the nature of bacterial innovation. Nature.

[224] Falagas M.E., Bliziotis I.A. (2007). Pandrug-resistant Gram-negative bacteria: The dawn of the post-antibiotic era?. Int. J. Antimicrob. Ag..

[225] Salyers A., Shoemaker N.B. (2006). Reservoirs of antibiotic resistance genes. Anim. Biotechnol..

[226] Biggel M., Boss S., Uea-Anuwong T., Lugsomya K., Magouras I., Stephan R. (2023). Complete Genome Sequence of the Extensively Drug-Resistant Extended-Spectrum β-Lactamase-Producing Proteus mirabilis Isolate HK294, Obtained from Poultry Feces in Hong Kong. Microbiol. Resour. Announc..

[227] He J., Sun L., Zhang L., Leptihn S., Yu Y., Hua X. (2021). A Novel SXT/R391 Integrative and Conjugative Element Carries Two Copies of the bla(NDM-1) Gene in Proteus mirabilis. mSphere.

[228] Peng K., Li Y., Wang Q., Yang P., Wang Z., Li R. (2023). Integrative conjugative elements mediate the high prevalence of tmexCD3-toprJ1b in Proteus spp. of animal source. mSystems.

[229] Zhou K., Xue C.X., Xu T., Shen P., Wei S., Wyres K.L., Lam M.M.C., Liu J., Lin H., Chen Y. (2023). A point mutation in recC associated with subclonal replacement of carbapenem-resistant Klebsiella pneumoniae ST11 in China. Nat. Commun..

[230] Demarre G., Frumerie C., Gopaul D.N., Mazel D. (2007). Identification of key structural determinants of the IntI1 integron integrase that influence attC attI1 recombination efficiency. Nucleic Acids Res..

[231] Jove T., Da Re S., Tabesse A., Gassama-Sow A., Ploy M.C. (2017). Gene expression in class 2 integrons is SOS-independent and involves two Pc promoters. Front. Microbiol..

[232] Richard E., Darracq B., Littner E., Vit C., Whiteway C., Bos J., Fournes F., Garriss G., Conte V., Lapaillerie D. (2024). Cassette recombination dynamics within chromosomal integrons are regulated by toxin-antitoxin systems. Sci. Adv..

[233] Mazel D. (2006). Integrons: Agents of bacterial evolution. Nat. Rev. Microbiol..

[234] Boucher Y., Labbate M., Koenig J.E., Stokes H.W. (2007). Integrons: Mobilizable platforms that promote genetic diversity in bacteria. Trends Microbiol..

[235] Cambray G., Guerout A.M., Mazel D. (2010). Integrons. Annu. Rev. Genet..

[236] Mantengoli E., Rossolini G.M. (2005). Tn 5393 d, a complex Tn 5393 derivative carrying the PER-1 extended-spectrum β-lactamase gene and other resistance determinants. Antimicrob Agents Chemother..

[237] Kargar M., Mohammadalipour Z., Doosti A., Lorzadeh S., Japoni-Nejad A. (2014). High prevalence of class 1 to 3 integrons among multidrug-resistant diarrheagenic Escherichia coli in southwest of Iran. Osong Public Health Res Perspect..

[238] Stokes H.W., Gillings M.R. (2011). Gene flow, mobile genetic elements and the recruitment of antibiotic resistance genes into Gram-negative pathogens. FEMS Microbiol. Rev..

[239] Sundstrom L., Radstrom P., Swedberg G., Skold O. (1988). Site-specific recombination promotes linkage between trimethoprim- and sulfonamide resistance genes. Sequence characterization of dhfrVand sulI and a recombination active locus of Tn21. Mol. Gen. Genet..

[240] Alqurashi E., Elbanna K., Ahmad I., Abulreesh H.H. (2022). Antibiotic Resistance in Proteus mirabilis: Mechanism, Status, and Public Health Significance. J. Pure Appl. Microbiol..

[241] Szabo O., Gulyas D., Szabo N., Kristof K., Kocsis B., Szabo D. (2018). Plasmid-mediated quinolone resistance determinants in Enterobacteriaceae from urine clinical samples. Acta Microbiol. Immunol. Hung..

[242] Liao H., Liu C., Zhou S., Liu C., Eldridge D.J., Ai C., Wilhelm S.W., Singh B.K., Liang X., Radosevich M. (2024). Prophage-encoded antibiotic resistance genes are enriched in human-impacted environments. Nat. Commun..

[243] Chegini Z., Khoshbayan A., Vesal S., Moradabadi A., Hashemi A., Shariati A. (2021). Bacteriophage therapy for inhibition of multidrug-resistant uropathogenic bacteria: A narrative review. Ann. Clin. Microbiol. Antimicrob..

[244] Valencia-Toxqui G., Sugumar S., Ramsey J. (2025). Isolation and characterization of biofilm-disrupting proteus phage Premi. Sci. Rep..

[245] Rozales F.P., Ribeiro V.B., Magagnin C.M., Pagano M., Lutz L., Falci D.R., Barth A.L. (2014). Emergence of NDM-1-producing Enterobacteriaceae in Porto Alegre, Brazil. Int. J. Infect. Dis..

[246] Kakoullis L., Papachristodoulou E., Chra P., Panos G. (2021). Mechanisms of Antibiotic Resistance in Important Gram-Positive and Gram-Negative Pathogens and Novel Antibiotic Solutions. Antibiotics.

[247] De Gaetano G.V., Lentini G., Famà A., Coppolino F., Beninati C. (2023). Antimicrobial Resistance: Two-Component Regulatory Systems and Multidrug Efflux Pumps. Antibiotics.

[248] Onanuga A., Oyi A.R., Olayinka B.O., Onaolapo J.A. (2012). Prevalence and susceptibility pattern of methicillin-resistant Staphylococcus aureus isolates among healthy women in Zaria, Nigeria. Afr. J. Biotechnol..

[249] Nikaido H. (2018). RNDTransporters in the Living World. Res. Microbiol..

[250] Yamasaki S., Zwama M., Yoneda T., Hayashi-Nishino M., Nishino K. (2023). Drug Resistance and Physiological Roles of RND Multidrug Efflux Pumps in *Salmonella enterica*, Escherichia coli and Pseudomonas aeruginosa: This Article Is Part of the Antimicrobial Efflux Collection. Microbiology.

[251] Lyu M., Ayala J.C., Chirakos I., Su C.-C., Shafer W.M., Yu E.W. (2022). Structural Basis of Peptide-Based Antimicrobial Inhibition of a Resistance-Nodulation-Cell Division Multidrug Efflux Pump. Microbiol. Spectr..

[252] Elshobary M.E., Badawy N.K., Ashraf Y., Zatioun A.A., Masriya H.H., Ammar M.M., Mohamed N.A., Mourad S., Assy A.M. (2025). Combating Antibiotic Resistance: Mechanisms, Multidrug-Resistant Pathogens, and Novel Therapeutic Approaches: An Updated Review. Pharmaceuticals.

[253] Zakaria N.F.S., Yahya M.F.Z.R., Jamil N.M. (2023). Multiple Bacterial Strategies to Survive Antibiotic Pressure: A Review. Preprints.

[254] Belay W.Y., Getachew M., Tegegne B.A., Teffera Z.H., Dagne A., Zeleke T.K., Abebe R.B., Gedif A.A., Fenta A., Yirdaw G. (2024). Mechanism of Antibacterial Resistance, Strategies and Next-Generation Antimicrobials to Contain Antimicrobial Resistance: A Review. Front. Pharmacol..

[255] Henderson R.K., Fendler K., Poolman B. (2019). Coupling Efficiency of Secondary Active Transporters. Curr. Opin. Biotechnol..

[256] Li D., Ge Y., Wang N., Shi Y., Guo G., Zou Q., Liu Q. (2023). Identification and Characterization of a Novel Major Facilitator Superfamily Efflux Pump, SA09310, Mediating Tetracycline Resistance in Staphylococcus aureus. Antimicrob. Agents Chemother..

[257] Vrancianu C.O., Gheorghe I., Czobor I.B., Chifiriuc M.C. (2020). Antibiotic Resistance Profiles, Molecular Mechanisms and Innovative Treatment Strategies of Acinetobacter baumannii. Microorganisms.

[258] Miyauchi H., Moriyama S., Kusakizako T., Kumazaki K., Nakane T., Yamashita K., Hirata K., Dohmae N., Nishizawa T., Ito K. (2017). Structural Basis for Xenobiotic Extrusion by Eukaryotic MATE Transporter. Nat. Commun..

[259] Hassan K.A., Liu Q., Elbourne L.D.H., Ahmad I., Sharples D., Naidu V., Chan C.L., Li L., Harborne S.P.D., Pokhrel A. (2018). Pacing across the Membrane: The Novel PACE Family of Efflux Pumps Is Widespread in Gram-Negative Pathogens. Res. Microbiol..

[260] Garneau-Tsodikova S., Labby K.J. (2016). Mechanisms of Resistance to Aminoglycoside Antibiotics: Overview and Perspectives. MedChemComm.

[261] Javed W. (2020). Study of the Conformational States of a Bacterial Multidrug ABC Transporter BmrA. Ph.D. Thesis.

[262] Mohammed E.A., Hassooni H.R., Khalaf N.M. (2024). Genomic Insights Into *Proteus mirabilis* and Antimicrobial Resistance. Cuмвoл нayкu.

[263] Boudjemaa H., Allem R., Fonkou M.D.M., el Houda Khennouchi N.C., Kerkoud M. (2019). Molecular drivers of emerging multidrug resistance in Proteus mirabilis clinical isolates from Algeria. J. Glob. Antimicrob. Resist..

[264] Castanheira M., Mills J.C., Costello S.E., Jones R.N., Sader H.S. (2015). Ceftazidime-avibactam activity tested against Enterobacteriaceae isolates from U.S. hospitals (2011 to 2013) and characterization of-lactamase-producing strains. Antimicrob. Agents Chemother..

[265] Ku Y.H., Lee M.F., Chuang Y.C., Yu W.L. (2019). Detection of plasmid mediated β-lactamase genes and emergence of a novel AmpC (CMH-1) in Enterobacter cloacae at a medical center in Southern Taiwan. J. Clin. Med..

[266] Ye Q., Wu Q., Zhang S., Zhang J., Yang G., Wang J., Xue L., Chen M. (2018). Characterization of extended spectrum β-lactamase-producing Enterobacteriaceae from retail food in China. Front. Microbiol..

[267] Kilic A., Bedir O., Kocak A., Bayram A., Balci I. (2017). Emergence of OXA-48-like carbapenemaseproducing Proteus mirabilis in Turkey. J. Infect. Dev. Ctries..

[268] Yang J.H., Sheng W.H., Hsueh P.R., SMART Program (2020). Antimicrobial susceptibility and distribution of extended-spectrum βlactamases, AmpC β-lactamases and carbapenemases among Proteus, Providencia and Morganella isolated from global hospitalised patients with intra-abdominal and urinary tract infections: Results of the Study for Monitoring Antimicrobial Resistance Trends (SMART), 2008–2011. J. Glob. Antimicrob. Resist..

[269] Smet A., Martel A., Persoons D., Dewulf J., Heyndrickx M., Herman L., Haesebrouck F., Butaye P. (2009). Broad-spectrum b-lactamases among Enterobacteriaceae of animal origin: Molecular aspects, mobility and impact on public health. FEMS Microbiol. Rev..

[270] Poirel L., Naas T., Nordmann P. (2010). Diversity, epidemiology, and genetics of class D b-lactamases. Antimicrob. Agents Chemother..

[271] Uzunovic S., Ibrahimagic A., Hodzic D., Bedenic B. (2016). Molecular epidemiology and antimicrobial susceptibility of AmpC-and/or extended-spectrum (ESBL) b-lactamase-producing *Proteus* spp. clinical isolates in Zenica-Doboj Canton, Bosnia and Herzegovina. Med. Glas..

[272] Han P., Luo L., Li M., Wang S., Han B., Gao J. (2020). Prevalence, Genetic Diversity and Antimicrobial Resistance of Proteus mirabilis Isolated from Dogs Hospitalized in Beijing. Pak. Vet. J..

[273] Cicek A.C., Duzgun A.O., Saral A., Sandalli C. (2014). Determination of a novel integron-located variant (blaOXA-320) of Class D β-lactamase in Proteus mirabilis. J. Basic. Microbiol..

[274] Perilli M., Dell’Amico E., Segatore B., de Massis M.R., Bianchi C., Luzzaro F., Rossolini G.M., Toniolo A., Nicoletti G., Amicosante G. (2002). Molecular characterization of extended-spectrum beta-lactamases produced by nosocomial isolates of Enterobacteriaceae from an Italian nationwide survey. J. Clin. Microbiol..

[275] Perilli M., Segatore B., Mugnaioli C., Celenza G., Rossolini G.M., Stefani S., Luzzaro F., Pini B., Amicosante G. (2011). Persistence of TEM-52/TEM-92 and SHV-12 extended-spectrum β-lactamases in clinical isolates of Enterobacteriaceae in Italy. Microb. Drug Resist..

[276] Bedenić B., Pospišil M., Nađ M., Bandić Pavlović D. (2025). Evolution of β-Lactam Antibiotic Resistance in Proteus Species: From Extended-Spectrum and Plasmid-Mediated AmpC β-Lactamases to Carbapenemases. Microorganisms.

[277] Nakama R., Shingaki A., Miyazato H., Higa R., Nagamoto C., Hamamoto K., Ueda S., Hachiman T., Touma Y., Miyagi K. (2016). Current status of extended spectrum β-lactamase-producing Escherichia coli, Klebsiella pneumoniae and Proteus mirabilis in Okinawa prefecture, Japan. J. Infect. Chemother..

[278] Karpenko A., Shelenkov A., Petrova L., Gusarov V., Zamyatin M., Mikhaylova Y., Akimin V. (2024). Two multidrug-resistant Proteus mirabilis clones carrying extended spectrum beta-lactamases revealed in a single hospital department by whole genome sequencing. Heliyon.

[279] Tian G.B., Jiang Y.Q., Huang Y.M., Qin Y., Feng L.Q., Zhang X.F., Li H.Y., Zhong L.L., Zeng K.J., Patil S. (2016). Characterization of CTX-M-140, a Variant of CTX-M-14 Extended-Spectrum β-Lactamase with Decreased Cephalosporin Hydrolytic Activity, from Cephalosporin-Resistant Proteus mirabilis. Antimicrob. Agents Chemother..

[280] Tseng B.S., Zhang W., Harrison J.J., Quach T.P., Song J.L., Penterman J., Singh P.K., Chopp D.L., Packman A.I., Parsek M.R. (2013). The extracellular matrix protects Pseudomonas aeruginosa biofilms by limiting the penetration of tobramycin. Environ. Microbiol..

[281] Bonnin R.A., Girlich D., Jousset A.B., Gauthier L., Cuzon G., Bogaerts P. (2020). A single Proteus mirabilis lineage from human and animal sources: A hidden reservoir of OXA-23 or OXA-58 carbapenemases in Enterobacterales. Sci. Rep..

[282] Versporten A., Zarb P., Caniaux I., Gros M.F., Drapier N., Miller M., Jarlier V., Nathwani D., Goossens H., Global-PPS network (2018). Antimicrobial consumption and resistance in adult hospital inpatients in 53 countries: Results of an internetbased global point prevalence survey. Lancet Glob. Health.

[283] Khademi F., Maarofi K., Arzanlou M., Peeri-Dogaheh H., Sahebkar A. (2021). Which missense mutations associated with DNA gyrase and topoisomerase IV are involved in Pseudomonas aeruginosa clinical isolates resistance to ciprofloxacin in Ardabil?. Gene Rep..

[284] Varughese L.R., Rajpoot M., Goyal S., Mehra R., Chhokar V., Beniwal V. (2018). Analytical profiling of mutations in quinolone resistance determining region of gyrA gene among UPEC. PLoS ONE.

[285] Nakano R., Nakano A., Abe M., Nagano N., Asahara M., Furukawa T., Ono Y., Yano H., Okamoto R. (2019). Prevalence and mechanism of fluoroquinolone resistance in clinical isolates of Proteus mirabilis in Japan. Heliyon.

[286] Zhang S., Sun J., Liao X.P., Hu Q.J., Liu B.T., Fang L.X., Deng H., Ma J., Xiao X., Zhu H.Q. (2013). Prevalence and plasmid characterization of the qnrD determinant in Enterobacteriaceae isolated from animals, retail meat products, and humans. Microb. Drug Resist..

[287] Alabi O.S., Mendonça N., Adeleke O.E., da Silva G.J. (2017). Molecular screening of antibiotic-resistant determinants among multidrug-resistant clinical isolates of Proteus mirabilis from SouthWest Nigeria. Afr. Health Sci..

[288] Jayol A., Janvier F., Guillard T., Chau F., Mérens A., Robert J., Fantin B., Berçot B., Cambau E. (2016). qnrA6 genetic environment and quinolone resistance conferred on Proteus mirabilis. J. Antimicrob. Chemother..

[289] Wang M., Guo Q., Xu X., Wang X., Ye X., Wu S., Hooper D.C., Wang M. (2009). New plasmid mediated quinolone resistance gene, qnrC, found in a clinical isolate of Proteus mirabilis. Antimicrob. Agents Chemother..

[290] Ogbolu D.O., Daini O.A., Ogunledun A., Alli A.O., Webber M.A. (2011). High levels of multidrug resistance in clinical isolates of Gram-negative pathogens from Nigeria. Int. J. Antimicrob. Agents.

[291] Mokracka J., Gruszczy´nska B., Kaznowski A. (2012). Integrons, β-lactamase and qnr genes in multidrug resistant clinical isolates of *Proteus mirabilis* and *P. vulgaris*. APMIS.

[292] Grossman T.H. (2016). Tetracycline antibiotics and resistance. Cold Spring Harb. Perspect. Med..

[293] Wang S., Gao X., Gao Y., Li Y., Cao M., Xi Z., Zhao L., Feng Z. (2017). Tetracycline resistance genes identified from distinct soil environments in China by functional metagenomics. Front. Microbiol..

[294] Kuleshov K.V., Pavlova A.S., Shedko E.D., Mikhaylova Y.V., Margos G., Hepner S., Chebotar I.V., Korneenko E.V., Podkolzin A.T., Akimkin V.G. (2021). Mobile colistin resistance genetic determinants of nontyphoid *Salmonella enterica* isolates from Russia. Microorganisms.

[295] Mirzaei A., Esfahani B.N., Raz A., Ghanadian M., Moghim S. (2021). From the Urinary Catheter to the Prevalence of Three Classes of Integrons, β-Lactamase Genes, and Differences in Antimicrobial Susceptibility of Proteus mirabilis and Clonal Relatedness with Rep-PCR. BioMed Res. Int..

[296] Zhang R., Dong N., Shen Z., Zeng Y., Lu J., Liu C., Zhou H., Hu Y., Sun Q., Cheng Q. (2020). Epidemiological and phylogenetic analysis reveals Flavobacteriaceae as potential ancestral source of tigecycline resistance gene tet (X). Nat. Commun..

[297] Singh S., Dubal Z.B., Kumar G.R., Tamta S., Saini S., Dosar S., Kr J., Rajendran V.O., Bhilegaonkar K.N., Holmes M. (2025). Genomic Constellation of Foodborne Proteus Mirabilis Isolates Harboring AMR, Virulence Genes and Comparative WGS Analysis. Curr. Microbiol..

[298] Castanheira M., Deshpande L.M., Woosley L.N., Serio A.W., Krause K.M., Flamm R.K. (2018). Activity of plazomicin compared with other aminoglycosides against isolates from European and adjacent countries, including Enterobacteriaceae molecularly characterized for aminoglycoside-modifying enzymes and other resistance mechanisms. J. Antimicrob. Chemother..

[299] Goodlet K.J., Benhalima F.Z., Nailor M.D. (2019). A systematic review of single-dose aminoglycoside therapy for urinary tract infection: Is it time to resurrect an old strategy?. Antimicrob. Agents Chemother..

[300] Kawai A., Suzuki M., Tsukamoto K., Minato Y., Doi Y. (2021). Functional and structural characterization of acquired 16s rRNA methyltransferase npmb1 conferring pan-aminoglycoside resistance. Antimicrob. Agents Chemother..

[301] Zarate S.G., De la Cruz Claure M.L., Benito-Arenas R., Revuelta J., Santana A.G., Bastida A. (2018). Overcoming aminoglycoside enzymatic resistance: Design of novel antibiotics and inhibitors. Molecules.

[302] Leulmi Z., Kandouli C., Mihoubi I., Benlabed K., Lezzar A., Rolain J.-M. (2019). First report of blaOXA-24 carbapenemase gene, armA methyltransferase and aac(6’)-Ib-cr among multidrug-resistant clinical isolates of Proteus mirabilis in Algeria. J. Glob. Antimicrob. Resist..

[303] Doi Y., Wachino J.I., Arakawa Y. (2016). Aminoglycoside resistance: The emergence of acquired 16S ribosomal RNA methyltransferases. Infect. Dis. Clin..

[304] Peterson E., Kaur P. (2018). Antibiotic resistance mechanisms in bacteria: Relationships between resistance determinants of antibiotic producers, environmental bacteria, and clinical pathogens. Front. Microbiol..

[305] Jacoby G.A. (2009). AmpC beta-lactamases. Clin. Microbiol. Rev..

[306] Li Z., Peng C., Zhang G., Shen Y., Zhang Y., Liu C., Liu M., Wang F. (2022). Prevalence and Characteristics of Multidrug-Resistant Proteus Mirabilis from Broiler Farms in Shandong Province, China. Poult. Sci..

[307] Sun L., He J., Shi X., Hu L., Yin Y., Yu Y., Hua X. (2023). Genotypic characterization of a Proteus mirabilis strain harboring blaKPC-2 on the IncN plasmid isolated from a patient with bloodstream infection in China. J. Infect. Public Health.

[308] Girlich D., Dortet L., Poirel L., Nordmann P. (2015). Integration of the blaNDM-1 carbapenemase gene into Proteus genomic island 1 (PGI1-PmPEL) in a Proteus mirabilis clinical isolate. J. Antimicrob. Chemother..

[309] Datta P., Gupta V., Arora S., Garg S., Chander J. (2014). Epidemiology of extended-spectrum-lactamase, AmpC, and carbapenemase production in Proteus mirabilis. Jpn. J. Infect. Dis..

[310] Aminov R.I., Mackie R.I. (2007). Evolution and ecology of antibiotic resistance genes. FEMS Microbiol. Lett..

[311] World Health Organization (2014). Antimicrobial Resistance: Global Report on Surveillance.

[312] Franklin A.M., Weller D.L., Durso L.M., Bagley M., Davis B.C., Frye J.G., Grim C.J., Ibekwe A.M., Jahne M.A., Keely S.P. (2024). A one health approach for monitoring antimicrobial resistance: Developing a national freshwater pilot effort. Front. Water.

[313] Summers A.O. (2002). Generally overlooked fundamentals of bacterial genetics and ecology. Clin. Infect. Dis..

[314] Nesse R.M., Stearns S.C. (2008). The great opportunity: Evolutionary applications to medicine and public health. Evol. Appl..

[315] Tacconelli E., Carrara E., Savoldi A. (2018). Discovery, research, and development of new antibiotics: The WHO priority list of antibiotic-resistant bacteria and tuberculosis. Lancet Infect. Dis..

[316] Bordier M., Binot A., Pauchard Q., Nguyen D.T., Trung T.N., Fortané N., Goutard F.L. (2018). Antibiotic resistance in Vietnam: Moving towards a One Health surveillance system. BMC Public Health.

[317] Shrestha K., Acharya K.P., Shrestha S. (2018). One Health: The interface between veterinary and human health. Int. J. One Health.

[318] Collignon P.J., McEwen S.A. (2019). One Health—Its importance in helping to better control antimicrobial resistance. Trop. Med. Infect. Dis..

[319] Kimani T., Kiambi S., Eckford S., Njuguna J., Makonnen Y., Rugalema G., Morzaria S.P., Lubroth J., Fasina F.O. (2019). Expanding beyond zoonoses: The benefits of a national One Health coordination mechanism to address antimicrobial resistance and other shared health threats at the human-animal-environment interface in Kenya. Rev. Sci. Tech..

[320] Landers T.F., Cohen B., Wittum T.E., Larson E.L. (2012). A Review of Antibiotic Use in Food Animals: Perspective, Policy, and Potential. Public Health Rep..

[321] Scott H.M., Acuff G., Bergeron G., Bourassa M.W., Gill J., Graham D.W., Kahn L.H., Morley P.S., Salois M.J., Simjee S. (2019). Critically important antibiotics: Criteria and approaches for measuring and reducing their use in food animal agriculture. Ann. N. Y. Acad. Sci..

[322] McEwen S.A., Collignon P.J. (2018). Antimicrobial Resistance: A One Health Perspective. Microbiol. Spectr..

[323] Cella E., Giovanetti M., Benedetti F., Scarpa F., Johnston C., Borsetti A., Ceccarelli G., Azarian T., Zella D., Ciccozzi M. (2023). Joining Forces against Antibiotic Resistance: The OneHealth Solution. Pathogens.

[324] Gongal G., Ofrin R.H., de Balogh K., Oh Y., Kugita H., Dukpa K. (2020). Operation alization of One Health and tripartite collaboration in the Asia-Pacific region. WHO South-East Asia J. Public Health.

[325] World Health Organization (2015). Global Action Plan on Antimicrobial Resistance.

[326] Mackenzie J.S., Jeggo M. (2019). The One Health Approach—Why Is It So Important?. Trop. Med. Infect. Dis..

[327] Buschhardt T., Günther T., Skjerdal T., Torpdahl M., Gethmann J., Filippitzi M.-E., Maassen C., Jore S., Ellis-Iversen J., Filter M. (2021). Aonehealth glossary to support communication and information exchange between the human health, animal health and food safety sectors. One Health.

[328] U.S. National Action Plan for Combating Antibiotic-Resistant Bacteria Progress Report: Year 5|ASPE. https://aspe.hhs.gov/reports/carb-year-5-report.

[329] WHO (2022). Strategic Framework for Collaboration on Antimicrobial Resistance—Together for One Health.

[330] Velazquez-Meza M.E., Galarde-López M., Carrillo-Quiróz B., Alpuche-Aranda C.M. (2022). Antimicrobial resistance: One Health approach. Vet. World.

[331] House W. (2015). National Action Plan for Combating Antibiotic-Resistant Bacteria.

[332] OPGA/WHO/FAO/OIE 2016 High-Level Meeting on Antimicrobial Resistance. https://www.un.org/pga/71/2016/09/21/press-release-hl-meeting-on-antimicrobial-resistance/.

[333] FAO/OIE/WHO 2017 The Tripartite’s Commitment—Providing Multi-Sectoral, Collaborative Leadership in Addressing Health Challenges. https://www.woah.org/app/uploads/2018/05/tripartite-2017.pdf.

[334] White A., Hughes J.M. (2019). Critical Importance of a One Health Approach to Antimicrobial Resistance. EcoHealth.

[335] Uchil R.R., Kohli G.S., Katekhaye V.M., Swami O.C. (2014). Strategies to combat antimicrobial resistance. J. Clin. Diagn. Res..

[336] Jacobsen S.M., Stickler D.J., Mobley H.L., Shirtliff M.E. (2008). Complicated catheter-associated urinary tract infections due to Escherichia coli and Proteus mirabilis. Clin. Microbiol. Rev..

[337] Fernández-Martínez M., Miró E., Ortega A., Bou G., González-López J.J., Oliver A., Pascual A., Cercenado E., Oteo J., Martínez-Martínez L. (2015). Molecular identification of integrons and antibiotic resistance genes in clinical isolates of Acinetobacter baumannii from 12 Spanish hospitals. Antimicrob. Agents Chemother..

[338] Holt K.E., Wertheim H., Zadoks R.N., Baker S., Whitehouse C.A., Dance D., Jenney A., Connor T.R., Hsu L.Y., Severin J. (2015). Genomic analysis of diversity, population structure, virulence, and antimicrobial resistance in Klebsiella pneumoniae, an urgent threat to public health. Proc. Natl. Acad. Sci. USA.

[339] Mahon C.R., Lehman D.C., Manuselis G. (2018). Textbook of Diagnostic Microbiology-E-Book.

[340] Bui T., Preuss C.V. (2022). Cephalosporin.

[341] Keam S.J. (2024). Cefepime/Enmetazobactam: First Approval. Drugs.

[342] Ronald A. (2002). The etiology of urinary tract infection: Traditional and emerging pathogens. Am. J. Med..

[343] Gupta R., Sharma S. (2022). Role of alternatives to antibiotics in mitigating the antimicrobial resistance crisis. Indian J. Med. Res..

[344] Bakken J.S., Borody T., Brandt L.J., Brill J.V., Demarco D.C., Franzos M.A., Kelly C., Khoruts A., Louie T., Martinelli L.P. (2011). Fecal Microbiota Transplantation Workgroup. Treating Clostridium difficile infection with fecal microbiota transplantation. Clin. Gastroenterol. Hepatol..

[345] Erriah P., Puan S.L., Yahaya N.M., Wan Ahmad Kamil W.N.I., Amin Nordin S., Muhamad A., Sabri S. (2025). Harnessing bacterial antimicrobial peptides: A comprehensive review on properties, mechanisms, applications, and challenges in combating antimicrobial resistance. J. Appl. Microbiol..

[346] Hazam P.K., Goyal R., Ramakrishnan V. (2019). Peptide based antimcrobials: Design strategies and therapeutic potential. Prog. Biophys Mol. Biol..

[347] Puan S.L., Erriah P., Baharudin M.M.A., Yahaya N.M., Kamil W.N.I.W.A., Ali M.S.M., Ahmad S.A., Oslan S.N., Lim S., Sabri S. (2023). Antimicrobial peptides from Bacillus spp. and strategies to enhance their yield. Appl. Microbiol. Biotechnol..

[348] Tincho M.B., Morris T., Meyer M., Pretorius A. (2020). Antibacterial activity of rationally designed antimicrobial peptides. Int. J. Microbiol..

[349] Mirkovic N., Polovic N., Vukotic G., Jovcic B., Miljkovic M., Radulovic Z., Diep D.B., Kojic M. (2016). Lactococcus lactis LMG2081 produces two bacteriocins, a nonlantibiotic and a novel lantibiotic. Appl. Environ. Microb..

[350] Maldonado-Barragán A., Alegría-Carrasco E., Blanco M.D.M., Vela A.I., Fernández-Garayzábal J.F., Rodríguez J.M., Gibello A. (2022). Garvicins AG1 and AG2: Two novel class iid bacteriocins of Lactococcus garvieae Lg-Granada. Int. J. Mol. Sci..

[351] Rahman M.S., Choi Y.H., Choi Y.S., Yoo J.C. (2017). Glycin-rich antimicrobial peptide YD1 from B. amyloliquefaciens, induced morphological alteration in and showed affinity for plasmid DNA of *E. coli*. AMB Expr..

[352] Cheung-Lee W.L., Parry M.E., Jaramillo Cartagena A., Darst S.A., Link A.J. (2019). Discovery and structure of the antimicrobial lasso peptide citrocin. J. Biol. Chem..

[353] Singh S.S., Sharma D., Singh C., Kumar S., Singh P., Sharma A., Das D.K., Pinnaka A.K., Thakur K.G., Ringe R.P. (2023). Brevicillin a novel lanthipeptide from the genus Brevibacillus with antimicrobial antifungal and antiviral activity. J. Appl. Microbiol..

[354] Xin B., Zheng J., Liu H., Li J., Ruan L., Peng D., Sajid M., Sun M. (2016). Thusin a novel two-component lantibiotic with potent antimicrobial activity against several Gram-positive pathogens. Front. Microbiol..

[355] Teng T., Li X., Zhang L., Li Y. (2020). Identification and characterization of pantocin wh-1, a novel cyclic polypeptide produced by *Pantoea dispersa* W18. Molecules.

[356] Moravej H., Moravej Z., Yazdanparast M., Heiat M., Mirhosseini A., Moosazadeh Moghaddam M., Mirnejad R. (2018). Antimicrobial peptides: Features action and their resistance mechanisms in bacteria. Microb. Drug Resist..

[357] Al-Ouqaili M.T., Ahmad A., Jwair N.A., Al-Marzooq F. (2025). Harnessing bacterial immunity: CRISPR-Cas system as a versatile tool in combating pathogens and revolutionizing medicine. Front. Cell. Infect. Microbiol..

[358] Agha A.S.A., Al-Samydai A., Aburjai T. (2025). New frontiers in CRISPR: Addressing antimicrobial resistance with Cas9, Cas12, Cas13, and Cas14. Heliyon.

[359] Dangi A.K., Sinha R., Dwivedi S., Gupta S.K., Shukla P. (2018). Cell line techniques and gene editing tools for antibody production: A review. Front. Pharmacol..

[360] Araya D.P., Palmer K.L., Duerkop B.A. (2021). CRISPR-based antimicrobials to obstruct antibiotic-resistant and pathogenic bacteria. PLoS Pathog..

[361] Yin Y., Wang P., Wang X., Wen J. (2023). Construction of Bacillus subtilis for efficient production of fengycin from xylose through CRISPR-Cas9. Front. Microbiol..

[362] Zhao K., Qiu L., Tao X., Zhang Z., Wei H. (2024). Genome analysis for cholesterol-lowing action and bacteriocin production of *Lactiplantibacillus plantarum* WLPL21 and ZDY04 from traditional Chinese fermented foods. Microorganisms.

[363] Sun Q., Kawuribi V., Xie Y., Xu H., Zheng S. (2026). CRISPR-Based Antimicrobials and Nanomotor Technologies for Drug-Resistant Biofilms. J. Drug Deliv. Sci. Technol..

[364] Gai Z., Samodelov S.L., Kullak-Ublick G.A., Visentin M. (2019). Molecular mechanisms of colistin-induced nephrotoxicity. Molecules.

[365] Voronko O.E., Khotina V.A., Kashirskikh D.A., Lee A.A., Gasanov V.A.O. (2025). Antimicrobial peptides of the cathelicidin family: Focus on LL-37 and its modifications. Int. J. Mol. Sci..

[366] Pulingam T., Parumasivam T., Gazzali A.M., Sulaiman A.M., Chee J.Y., Lakshmanan M., Chin C.F., Sudesh K. (2022). Antimicrobial resistance: Prevalence, economic burden, mechanisms of resistance and strategies to overcome. Eur. J. Pharm. Sci..

[367] Mahamoud A., Chevalier J., Alibert-Franco S., Kern W.V., Pagès J.M. (2007). Antibiotic efflux pumps in Gram-negative bacteria: The inhibitor response strategy. J. Antimicrob. Chemother..

[368] Kvist M., Hancock V., Klemm P. (2008). Inactivation of efflux pumps abolishes bacterial biofilm formation. Appl. Environ. Microbiol..

[369] Akturk E., Oliveira H., Santos S.B., Costa S., Kuyumcu S., Melo L.D., Azeredo J. (2019). Synergistic action of phage and antibiotics: Parameters to enhance the killing efficacy against mono and dual-species biofilms. Antibiotics.

[370] Kumar M., Sarma D.K., Shubham S., Kumawat M., Verma V., Nina P.B., Jp D., Kumar S., Singh B., Tiwari R.R. (2021). Futuristic non-antibiotic therapies to combat antibiotic resistance: A review. Front. Microbiol..

[371] Devi N.S., Mythili R., Cherian T., Dineshkumar R., Sivaraman G.K., Jayakumar R., Prathaban M., Duraimurugan M., Chandrasekar V., Peijnenburg W.J. (2024). Overview of antimicrobial resistance and mechanisms: The relative status of the past and current. Microbe.

[372] Pang X., Liu X., Cheng Y., Zhang C., Ren E., Liu C., Zhang Y., Zhu J., Chen X., Liu G. (2019). Sono-immunotherapeutic Nanocapturer to Combat Multidrug-resistant Bacterial Infections. Adv. Mater..

[373] Szczerbiec D., Bednarska-Szczepaniak K., Torzewska A. (2024). Antibacterial properties and urease suppression ability of Lactobacillus inhibit the development of infectious urinary stones caused by Proteus mirabilis. Sci. Rep..

[374] Chamundeeswari M., Jeslin J., Verma M.L. (2019). Nanocarriers for drug delivery applications. Environ. Chem. Lett..

[375] Alabdali A.Y.M., Kzar M.S., Chinnappan S., Mani R.R., Selvaraja M., Wen K.J., Sally L., Kuang F.W. (2022). Application of nanoantibiotics approach against anti-bacterial resistance. Int. J. Appl. Pharm..

[376] Rello J., Valenzuela-Sánchez F., Ruiz-Rodriguez M., Moyano S. (2017). Sepsis: A review of advances in management. Adv. Ther..

[377] Seymour C.W., Kahn J.M., Martin-Gill C., Callaway C.W., Yealy D.M., Scales D., Angus D.C. (2017). Delays from first medical contact to antibiotic administration for sepsis. Crit. Care Med..

[378] De Waele J.J., Akova M., Antonelli M., Canton R., Carlet J., De Backer D., Dimopoulos G., Garnacho-Montero J., Kesecioglu J., Lipman J. (2018). Antimicrobial resistance and antibiotic stewardship programs in the ICU: Insistence and persistence in the fight against resistance. A position statement from ESICM/ESCMID/ WAAAR round table on multi-drug resistance. Intensive Care Med..

[379] Diaz M.I., Cooper L.N., Hanna J.J., Beauchamp A.M., Ingle T.A., Wakene A.D., Most Z., Perl T., Katterpalli C., Keller T. (2025). Integrating socioeconomic deprivation indices and electronic health record data to predict antimicrobial resistance. Npj Antimicrob. Resist..

[380] Corbin C.K., Medford R.J., Osei K., Chen J.H. (2020). Personalized antibiograms: Machine learning for precision selection of empiric antibiotics. AMIA Jt. Summits Transl. Sci. Proc..

[381] Sherry N.L., Lee J.Y., Giulieri S.G., Connor C.H., Horan K., Lacey J.A., Lane C.R., Carter G.P., Seemann T., Egli A. (2025). Genomics for antimicrobial resistance—Progress and future directions. Antimicrob. Agents Chemother..

[382] Ajayi O.O., Adetunji C.O., Sharifi-Rad J. (2024). Chapter 19—Toxicity and Safety of Essential Oil. Applications of Essential Oils in the Food Industry.

[383] Abd-ElGawad A.M., El Gendy A.E.-N.G., Assaeed A.M., Al-Rowaily S.L., Alharthi A.S., Mohamed T.A., Nassar M.I., Dewir Y.H., Elshamy A.I. (2021). Phytotoxic Effects of Plant Essential Oils: A Systematic Review and Structure-Activity Relationship Based on Chemometric Analyses. Plants.

[384] Shoueir K.R., El-Desouky N., Rashad M.M., Ahmed M.K., Janowska I., El-Kemary M. (2021). Chitosan based-nanoparticles and nanocapsules: Overview, physicochemical features, applications of a nanofibrous scaffold, and bioprinting. Int. J. Biol. Macromol..

[385] Qi L., Xu Z., Jiang X., Hu C., Zou X. (2004). Preparation and antibacterial activity of chitosan nanoparticles. Carbohydr. Res..

[386] Hasan S.A. (2025). Evaluation of Trimethoprim Nanoemulsion for Combating Antibiotic-Resistant Proteus mirabilis in Urinary Tract Infections. Iran. J. Med. Microbiol..

[387] Nissanka N.M.C., Priyadarshana G., Dilhari K.A.A., Munasinghe J.A., Dilshani M., Weerasekera M.M. (2025). Curcumin-Modified Silver Nanoparticles’ Bioactivities Against Biofilm Forming, Multidrug-Resistant, Uropathogenic Proteus mirabilis. Bionanoscience.

[388] Ahmed M.E., Hamza Faiq N., Almutairi H.H., Alam M.W. (2025). Biosynthesized ZnO-CuO Nanocomposite for Biofilm Formation of Proteus mirabilis upon LuxS Gene Expression. Inorganics.

[389] Torabi S., Keshavarzi F. (2025). Agonistic and Antagonistic Effects of both Aqueous and Alcoholic Extracts of Plants containing Copper and Silver Nanoparticles on *Escherichia coli* and *Proteus mirabilis*. Curr. Res. Green. Sustain. Chem..

[390] Gurkok S., Ozdal M., Cakici T., Kurbanoglu E.B. (2025). Antimicrobial, antibiofilm, and antiurease activities of green-synthesized Zn, Se, and ZnSe nanoparticles against *Streptococcus salivarius* and *Proteus mirabilis*. Bioprocess. Biosyst. Eng..

[391] Elshikiby L.A., Baka Z.A., El-Zahed M.M. (2025). Biological activities of optimized biosynthesized selenium nanoparticles using Proteus mirabilis PQ350419 alone or combined with chitosan and ampicillin against common multidrug-resistant bacteria. Microb. Cell Fact..

[392] Xie M., Gao M., Yun Y., Malmsten M., Rotello V.M., Zboril R., Akhavan O., Kraskouski A., Amalraj J., Cai X. (2023). Antibacterial Nanomaterials: Mechanisms, Impacts on Antimicrobial Resistance and Design Principles. Angew. Chem. Int. Ed. Engl..

[393] Price L., Gozdzielewska L., Young M., Smith F., MacDonald J., McParland J., Williams L., Langdridge D., Davis M., Flowers P. (2018). Effectiveness of interventions to improve the public’s antimicrobial resistance awareness and behaviours associated with prudent use of antimicrobials: A systematic review. J. Antimicrob. Chemother..

[394] Chang Q., Wang W., Regev-Yochay G., Lipsitch M., Hanage W.P. (2015). Antibiotics in agriculture and the risk to human health: How worried should we be?. Evol. Appl..

[395] Engberg J., Aarestrup F.M., Taylor D.E., Gerner-Smidt P., Nachamkin I. (2001). Quinolone and macrolide resistance in Campylobacter jejuni and C. coli: Resistance mechanisms and trends in human isolates. Emerg. Infect. Dis..

[396] Gupta A., Nelson J.M., Barrett T.J., Tauxe R.V., Rossiter S.P., Friedman C.R., Joyce K.W., Smith K.E., Jones T.F., Haw-kins M.A. (2004). Antimicrobial Resistance among Campylobacter Strains, United States, 1997–2001. Emerg. Infect. Dis..

[397] GBD 2021 Antimicrobial Resistance Collaborators (2024). Global burden of bacterial antimicrobial resistance 1990–2021: A systematic analysis with forecasts to 2050. Lancet.

[398] Malani A.N., Sharland M., Clancy C.J., Skov R. (2024). A global call to action to fight antimicrobial resistance: IDSA and ESCMID joint white paper. Open Forum Infect. Dis..

[399] Al-Jedai A.H., Almogbel Y., Eljaaly K., Alqahtani N.M., Almudaiheem H.Y., Awad N., Alissa A.D., Assiri A., Alaama T. (2022). Restriction on antimicrobial dispensing without prescription on a national level: Impact on the overall antimicrobial utilization in the community pharmacies in Saudi Arabia. PLoS ONE.

[400] Bhandary S. (2024). Kerala Takes Pioneering Step Against Antimicrobial Resistance. https://www.medboundtimes.com/medbound-blog/kerala-takes-pioneering-step-against-antimicrobial-resistance.

[401] Gautam S., Das D.K., Kaur J., Kumar A., Ubaidullah M., Hasan M., Yadav K.K., Gupta R.K. (2023). Transition metal-based nanoparticles as potential antimicrobial agents: Recent advancements, mechanistic, challenges, and future prospects. Discov. Nano.

